# Supplementary figures and images for: TERRA R-loops trigger a switch in telomere maintenance towards break-induced replication and PRIMPOL-dependent repair
Source: EMBO J. 2025 Jul 7;44(16):4525–51. doi: 10.1038/s44318-025-00502-4 (PMC12361433; doi:10.1038/s44318-025-00502-4)

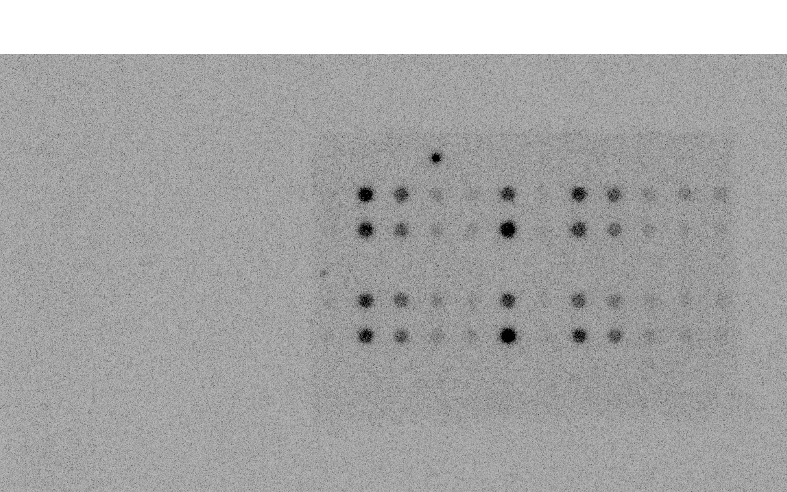

Supplement: Supplementary file 2 — Source data Fig. 1 [file 44318_2025_502_MOESM2_ESM.zip › Figure 1/Fig 1D/Fig 1D.jpg]

**Fig 1D**

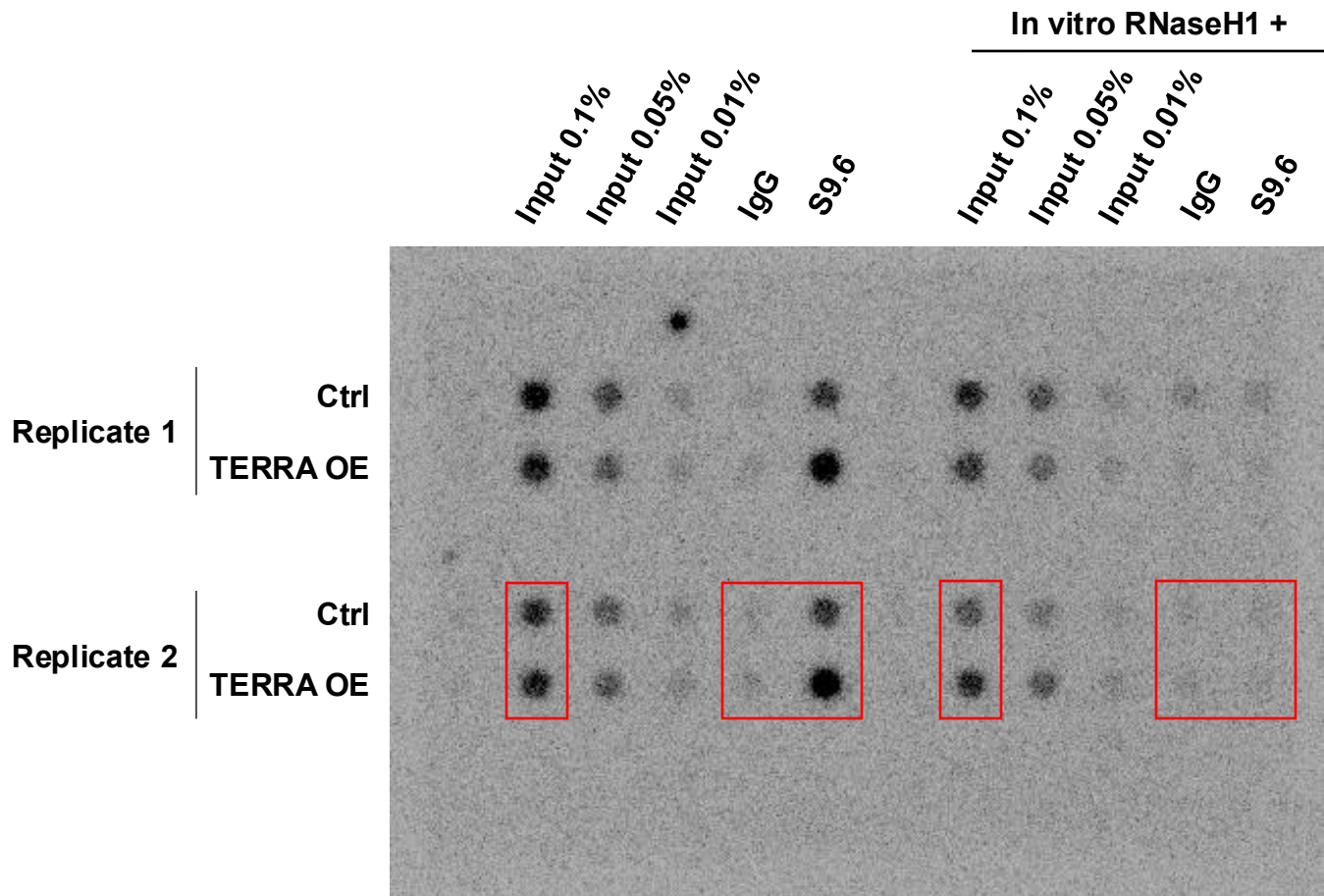

Supplement: Supplementary file 2 — Source data Fig. 1 [file 44318_2025_502_MOESM2_ESM.zip › Figure 1/Fig 1D/Fig 1D.pdf]

Fig 1B

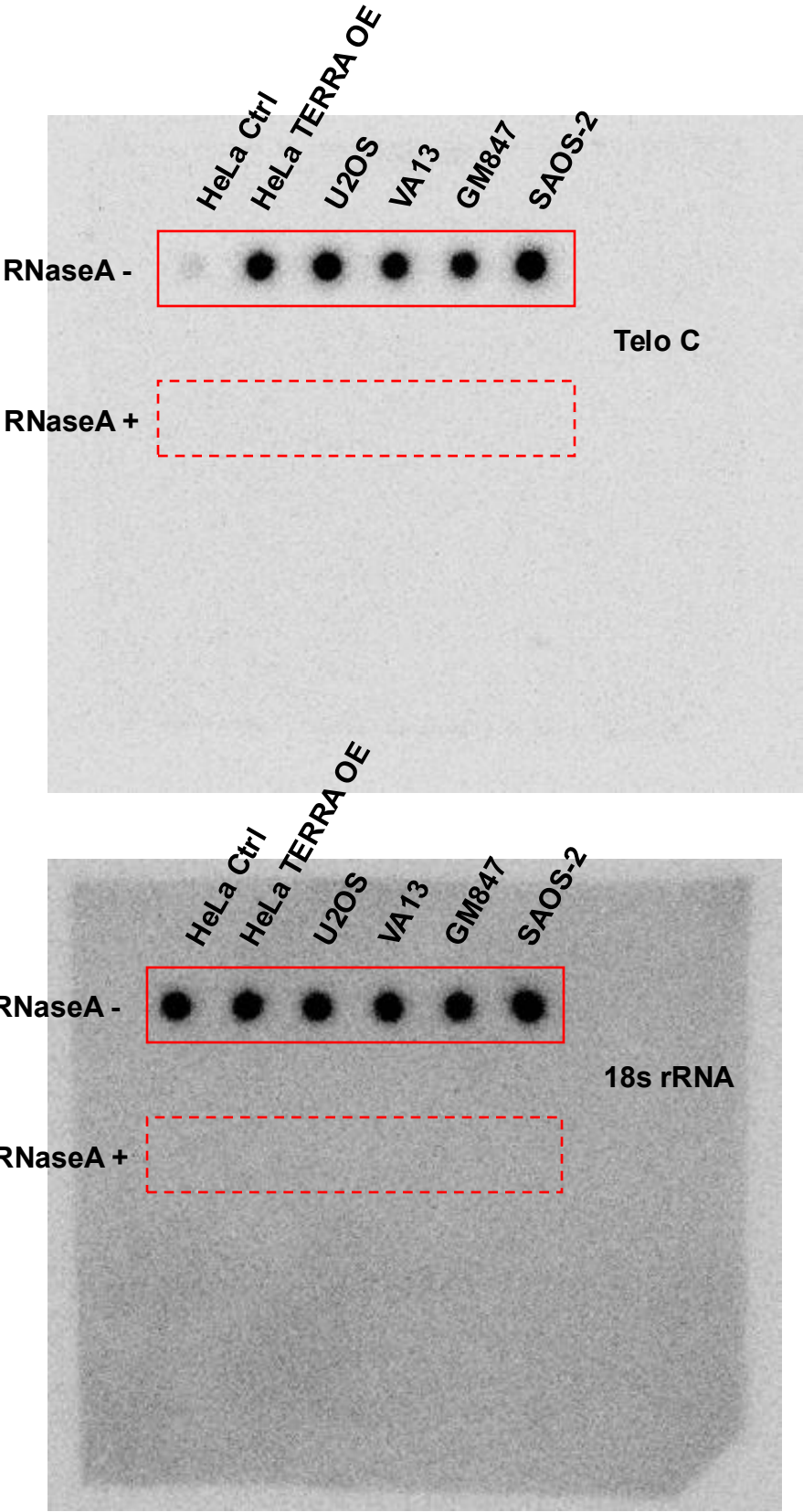

Supplement: Supplementary file 2 — Source data Fig. 1 [file 44318_2025_502_MOESM2_ESM.zip › Figure 1/Fig 1B/Fig 1B.pdf]

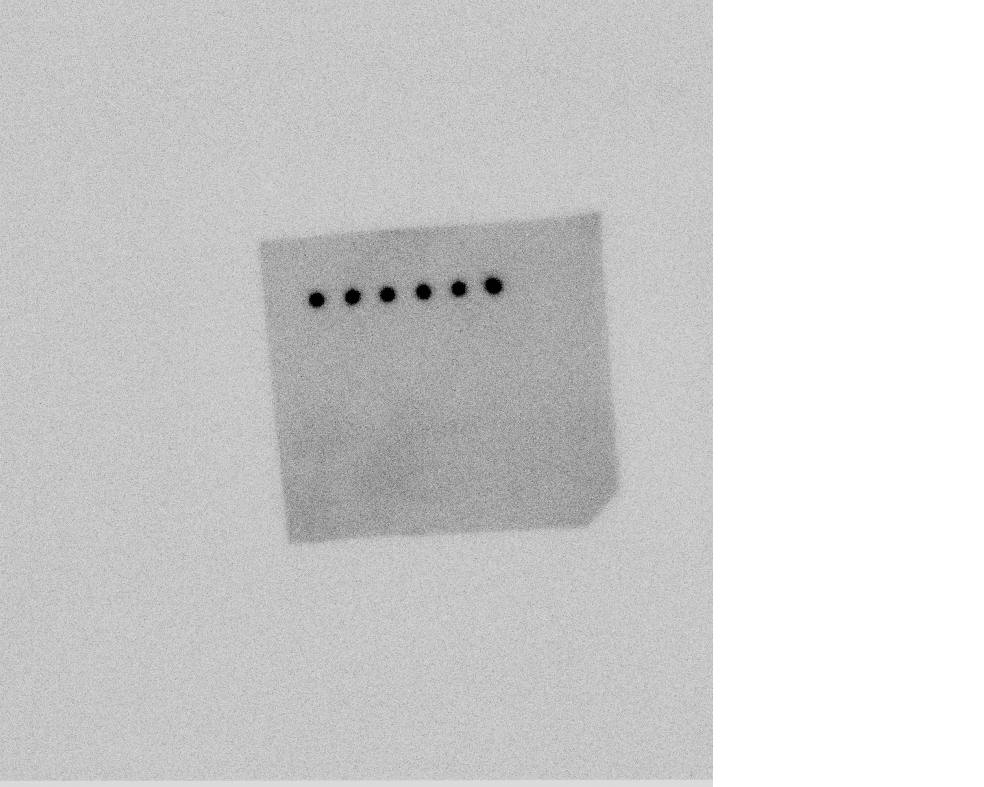

Supplement: Supplementary file 2 — Source data Fig. 1 [file 44318_2025_502_MOESM2_ESM.zip › Figure 1/Fig 1B/Fig 1B-18s rRNA.jpg]

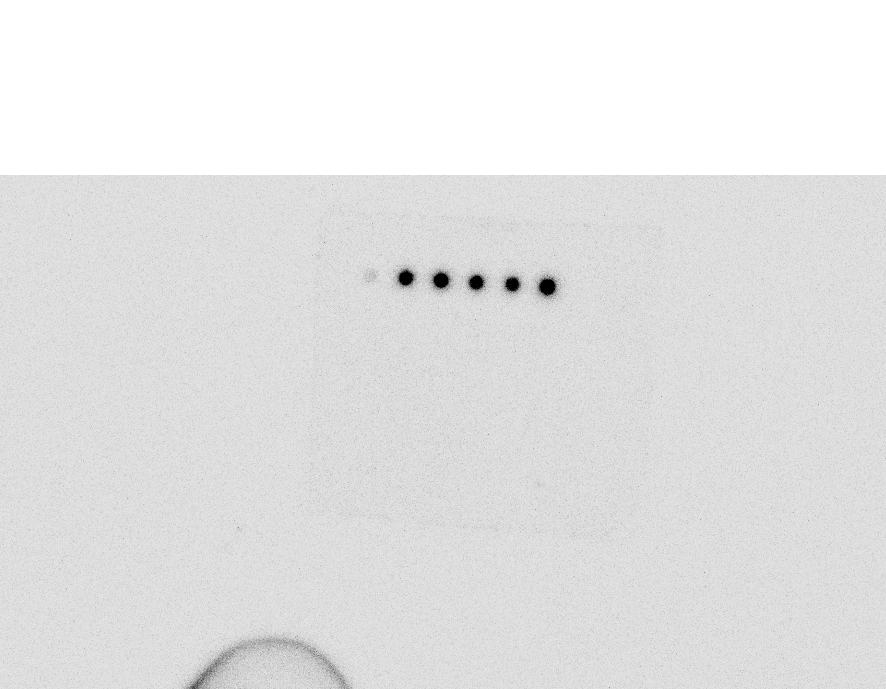

Supplement: Supplementary file 2 — Source data Fig. 1 [file 44318_2025_502_MOESM2_ESM.zip › Figure 1/Fig 1B/Fig 1B-Telo C.jpg]

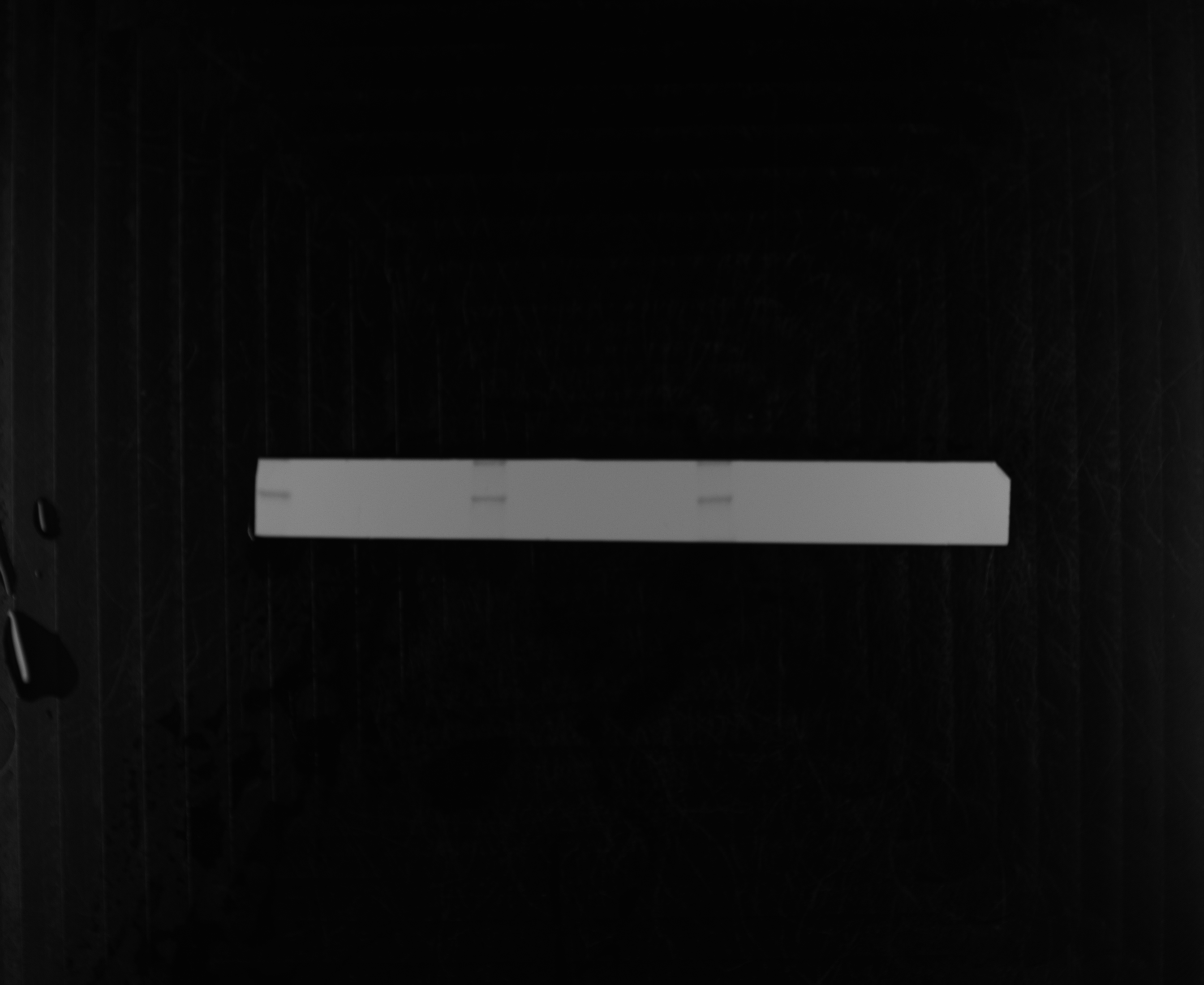

Supplement: Supplementary file 3 — Source data Fig. 2 [file 44318_2025_502_MOESM3_ESM.zip › Figure 2/Fig 2A/Vinculin - marker.Tif]

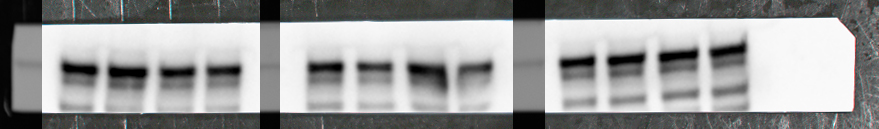

Supplement: Supplementary file 3 — Source data Fig. 2 [file 44318_2025_502_MOESM3_ESM.zip › Figure 2/Fig 2A/Vinculin - merge.jpg]

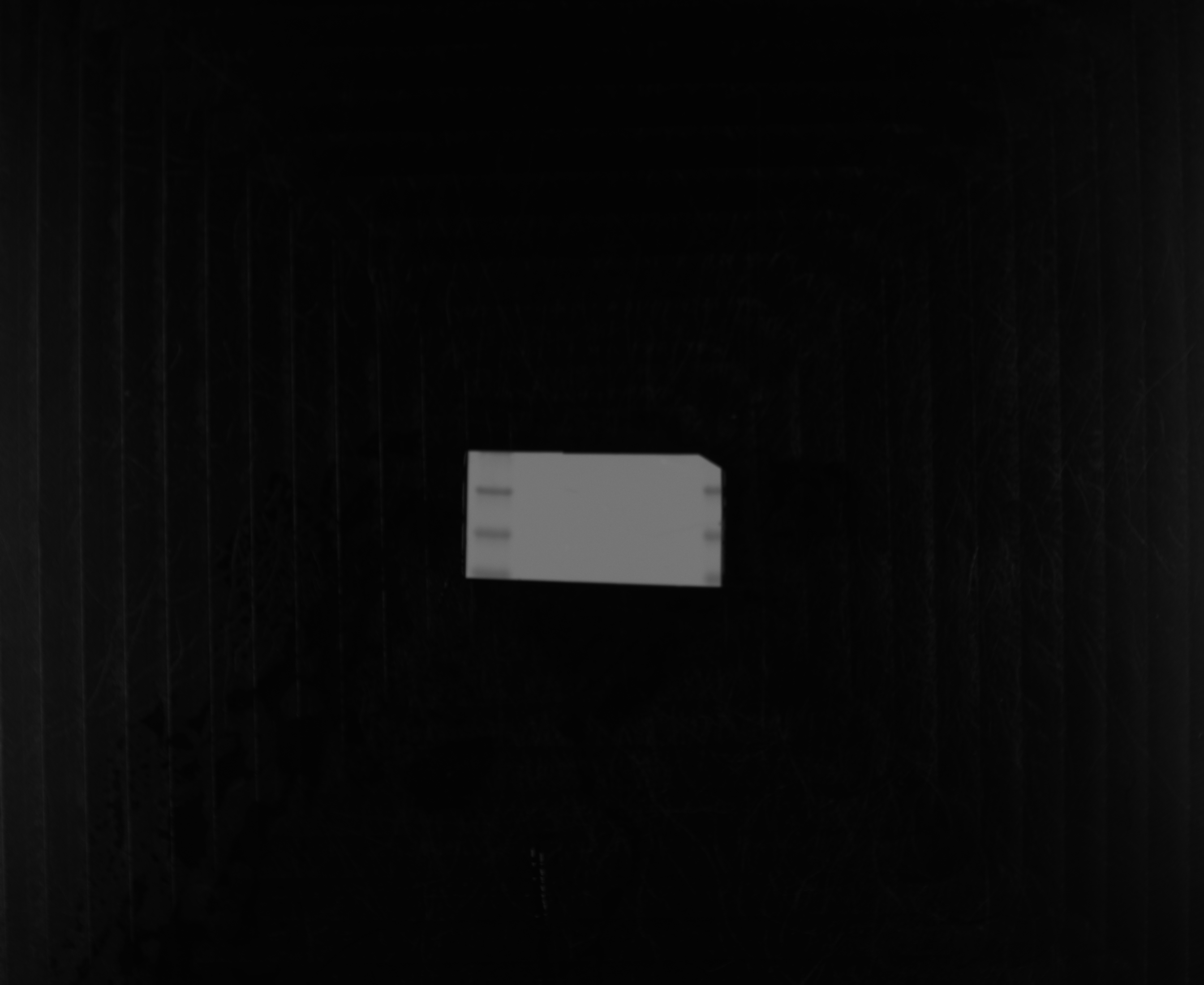

Supplement: Supplementary file 3 — Source data Fig. 2 [file 44318_2025_502_MOESM3_ESM.zip › Figure 2/Fig 2A/RAD51AP1 - marker.Tif]

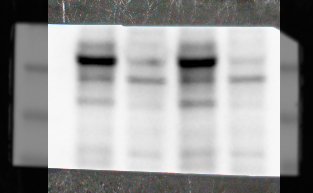

Supplement: Supplementary file 3 — Source data Fig. 2 [file 44318_2025_502_MOESM3_ESM.zip › Figure 2/Fig 2A/RAD51AP1 - merge.jpg]

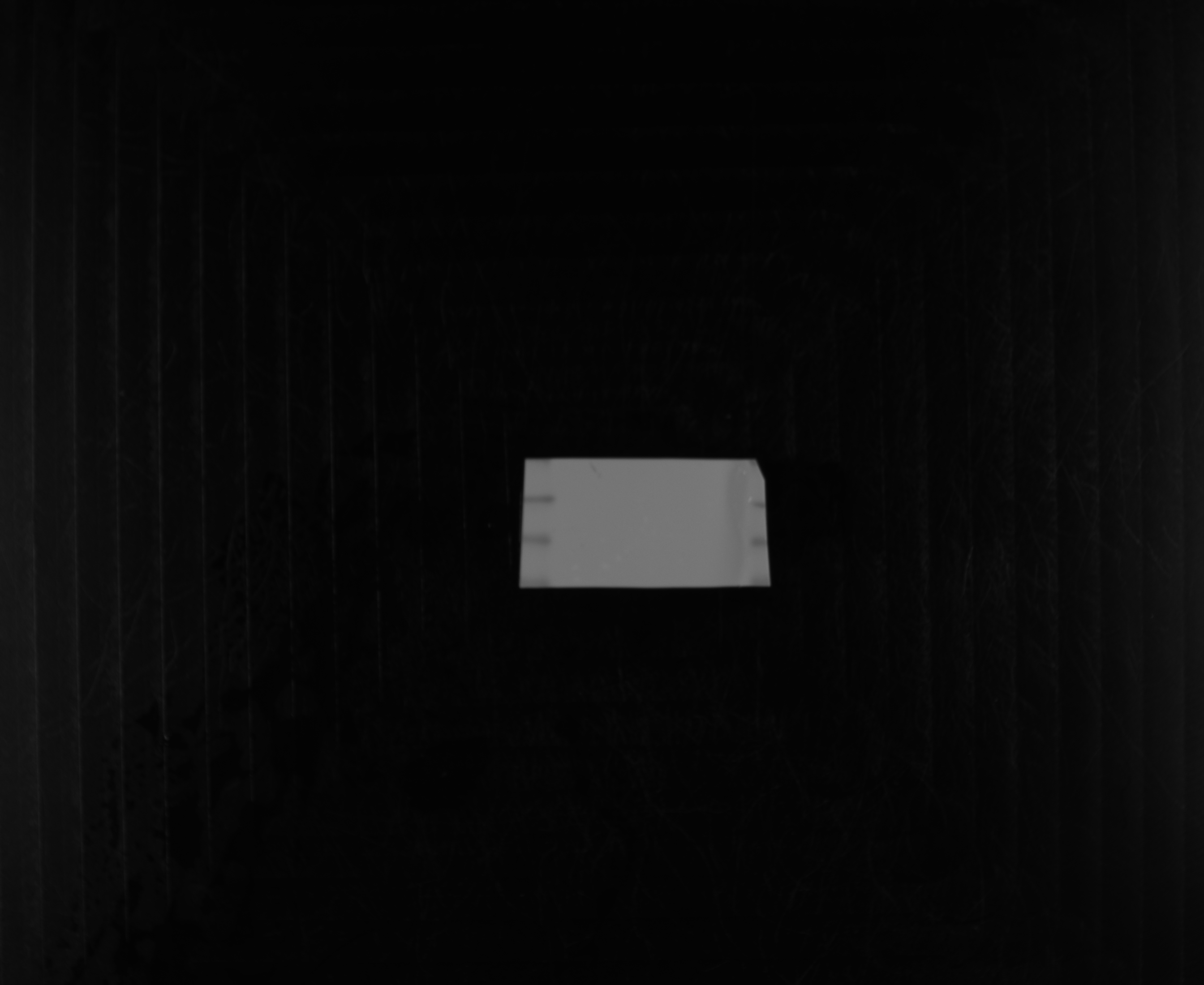

Supplement: Supplementary file 3 — Source data Fig. 2 [file 44318_2025_502_MOESM3_ESM.zip › Figure 2/Fig 2A/RAD51 - marker.Tif]

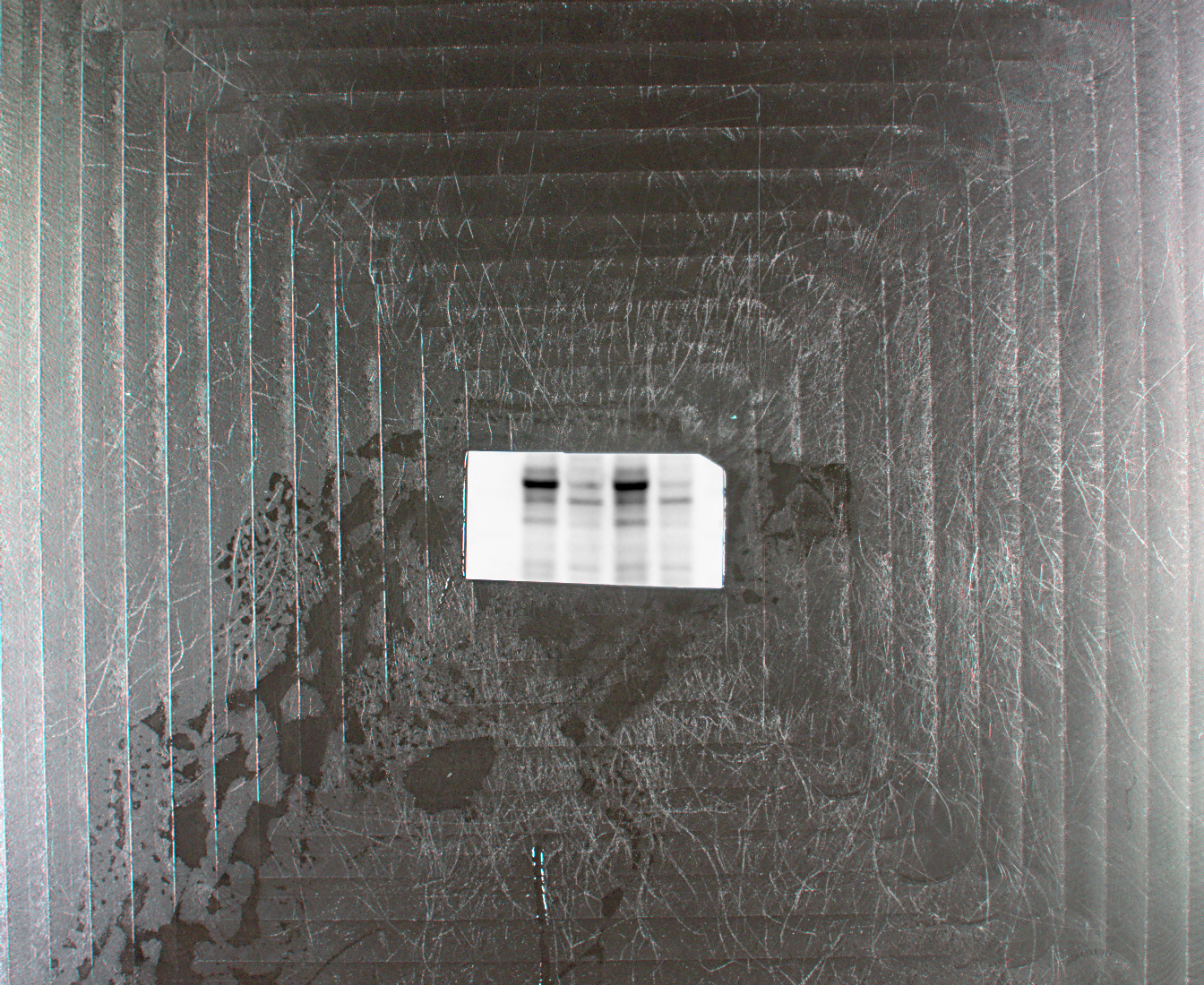

Supplement: Supplementary file 3 — Source data Fig. 2 [file 44318_2025_502_MOESM3_ESM.zip › Figure 2/Fig 2A/RAD51AP1.Tif]

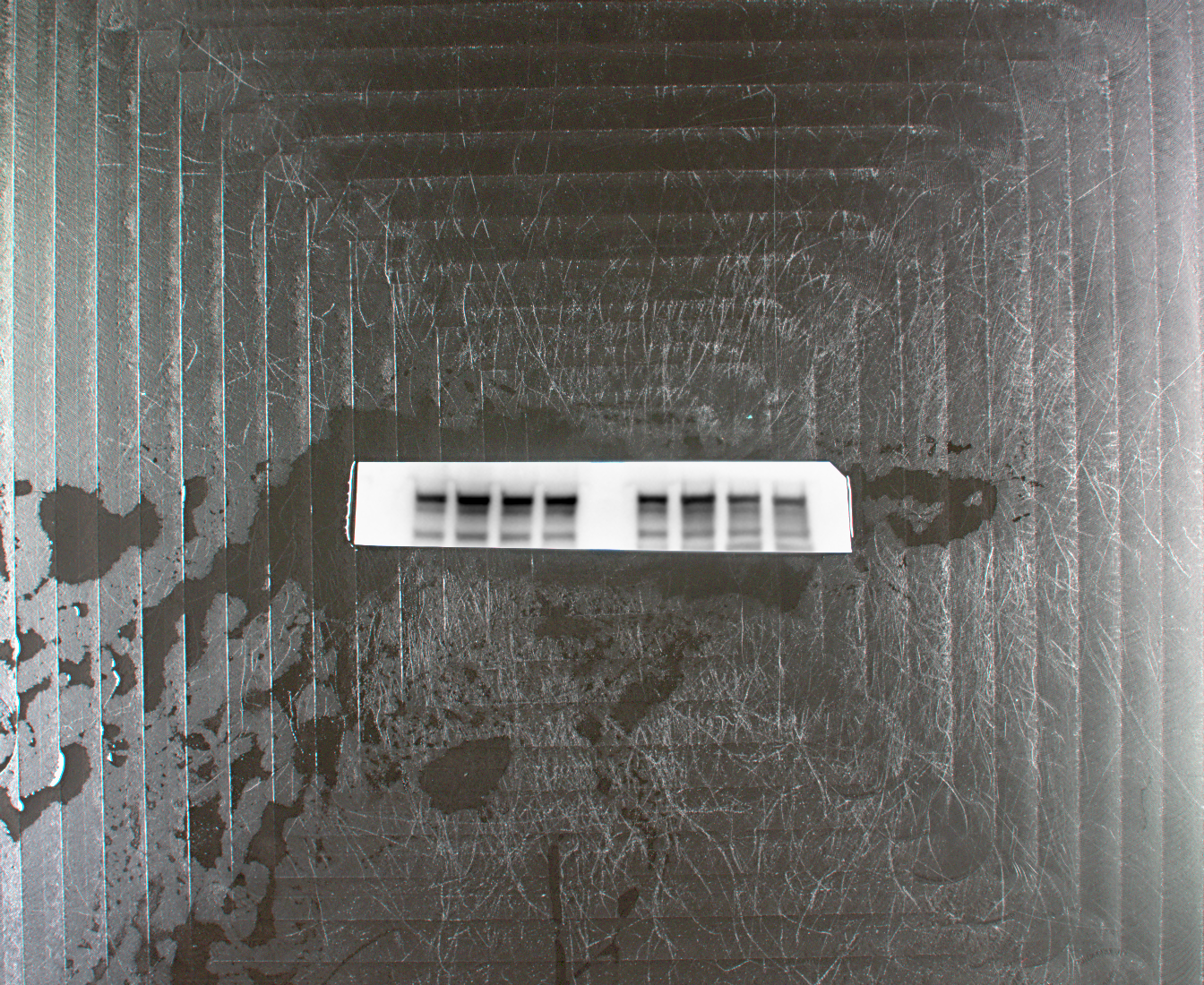

Supplement: Supplementary file 3 — Source data Fig. 2 [file 44318_2025_502_MOESM3_ESM.zip › Figure 2/Fig 2A/Vinculin (2).Tif]

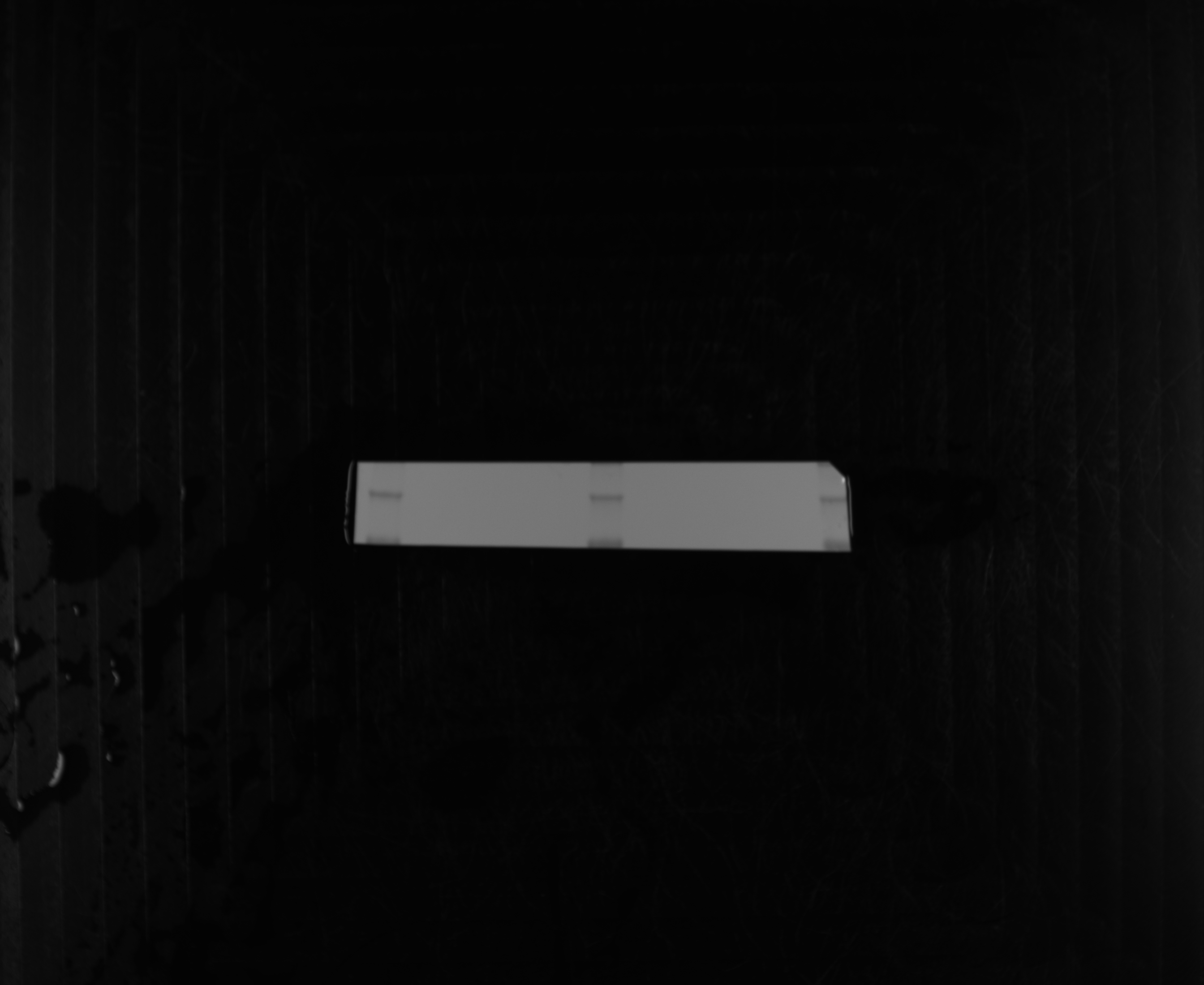

Supplement: Supplementary file 3 — Source data Fig. 2 [file 44318_2025_502_MOESM3_ESM.zip › Figure 2/Fig 2A/Vinculin (2) - marker.Tif]

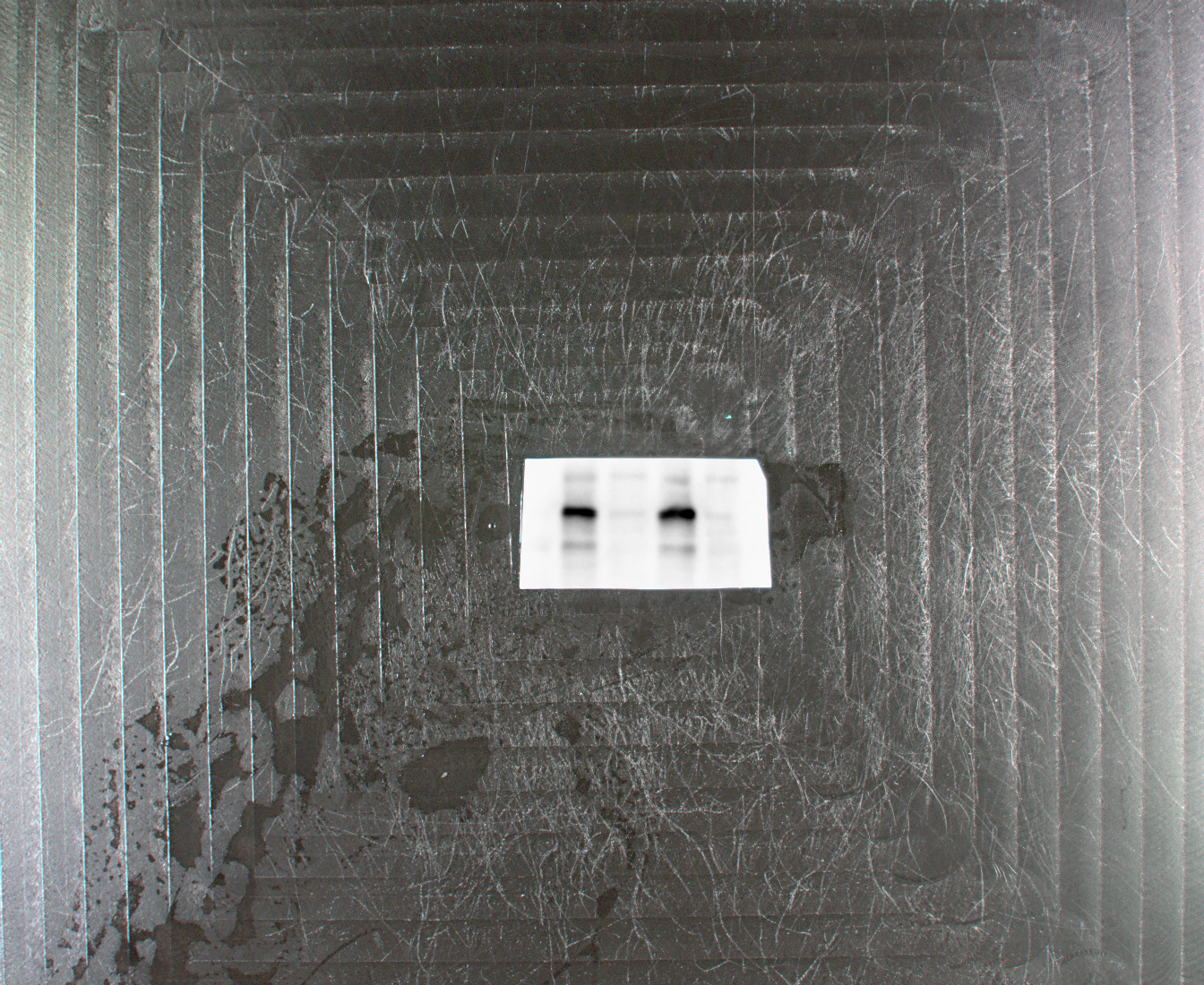

Supplement: Supplementary file 3 — Source data Fig. 2 [file 44318_2025_502_MOESM3_ESM.zip › Figure 2/Fig 2A/RAD51.Tif]

Fig 2A

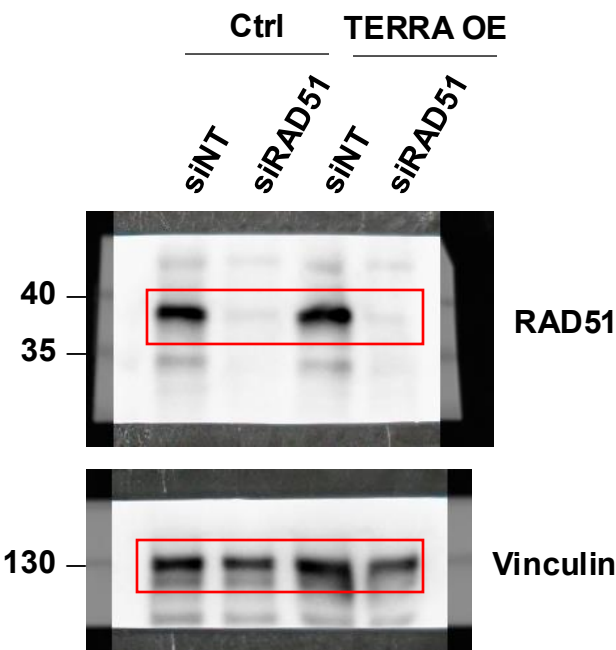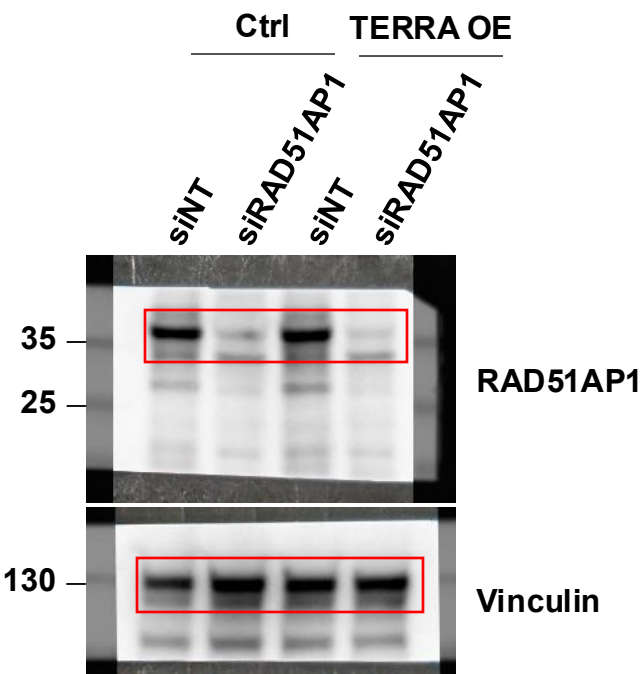

Supplement: Supplementary file 3 — Source data Fig. 2 [file 44318_2025_502_MOESM3_ESM.zip › Figure 2/Fig 2A/Fig 2A.pdf]

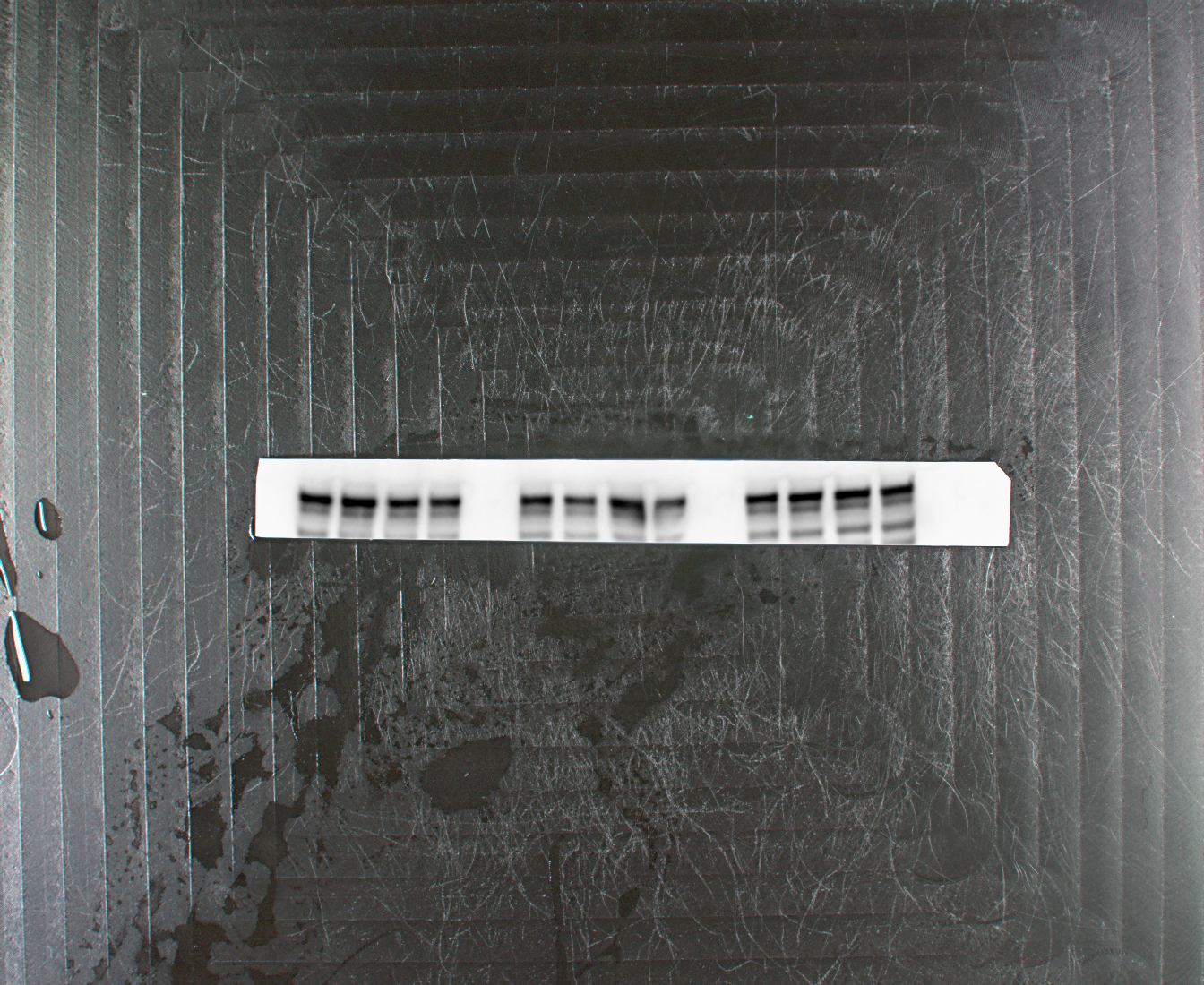

Supplement: Supplementary file 3 — Source data Fig. 2 [file 44318_2025_502_MOESM3_ESM.zip › Figure 2/Fig 2A/Vinculin.Tif]

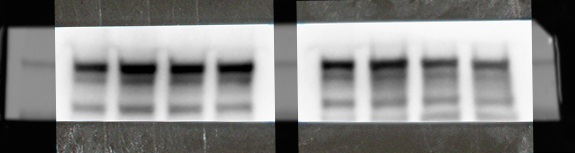

Supplement: Supplementary file 3 — Source data Fig. 2 [file 44318_2025_502_MOESM3_ESM.zip › Figure 2/Fig 2A/Vinculin (2) - merge.jpg]

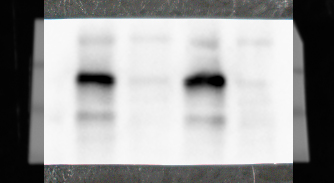

Supplement: Supplementary file 3 — Source data Fig. 2 [file 44318_2025_502_MOESM3_ESM.zip › Figure 2/Fig 2A/RAD51 - merge.jpg]

Fig 2B

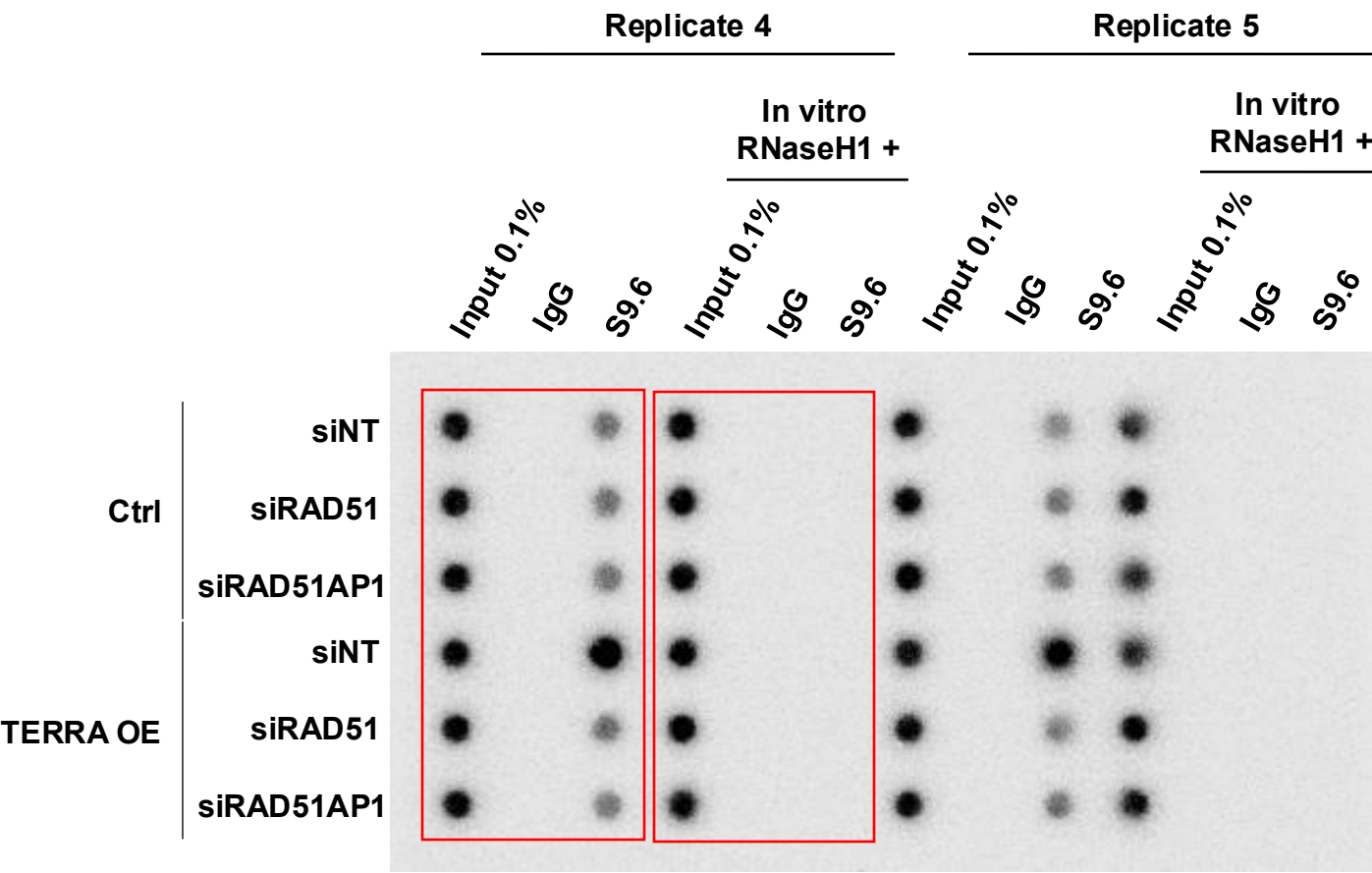

Supplement: Supplementary file 3 — Source data Fig. 2 [file 44318_2025_502_MOESM3_ESM.zip › Figure 2/Fig 2B/Fig 2B.pdf]

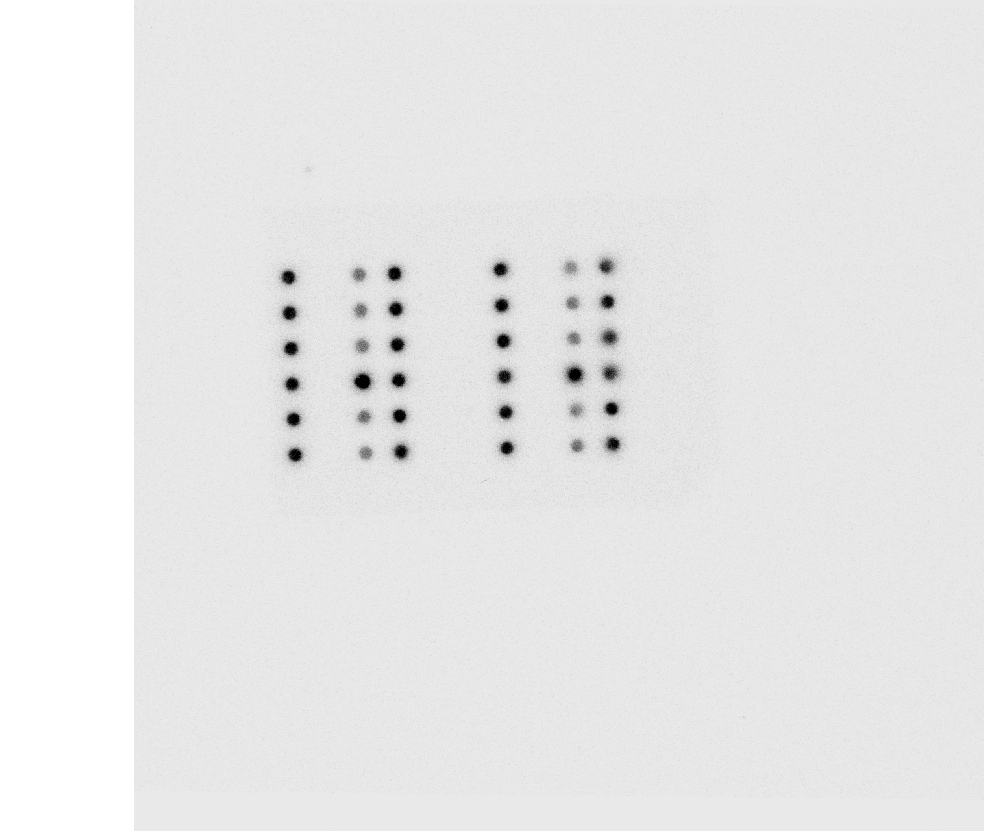

Supplement: Supplementary file 3 — Source data Fig. 2 [file 44318_2025_502_MOESM3_ESM.zip › Figure 2/Fig 2B/Fig 2B.jpg]

Fig 3E

Ctrl

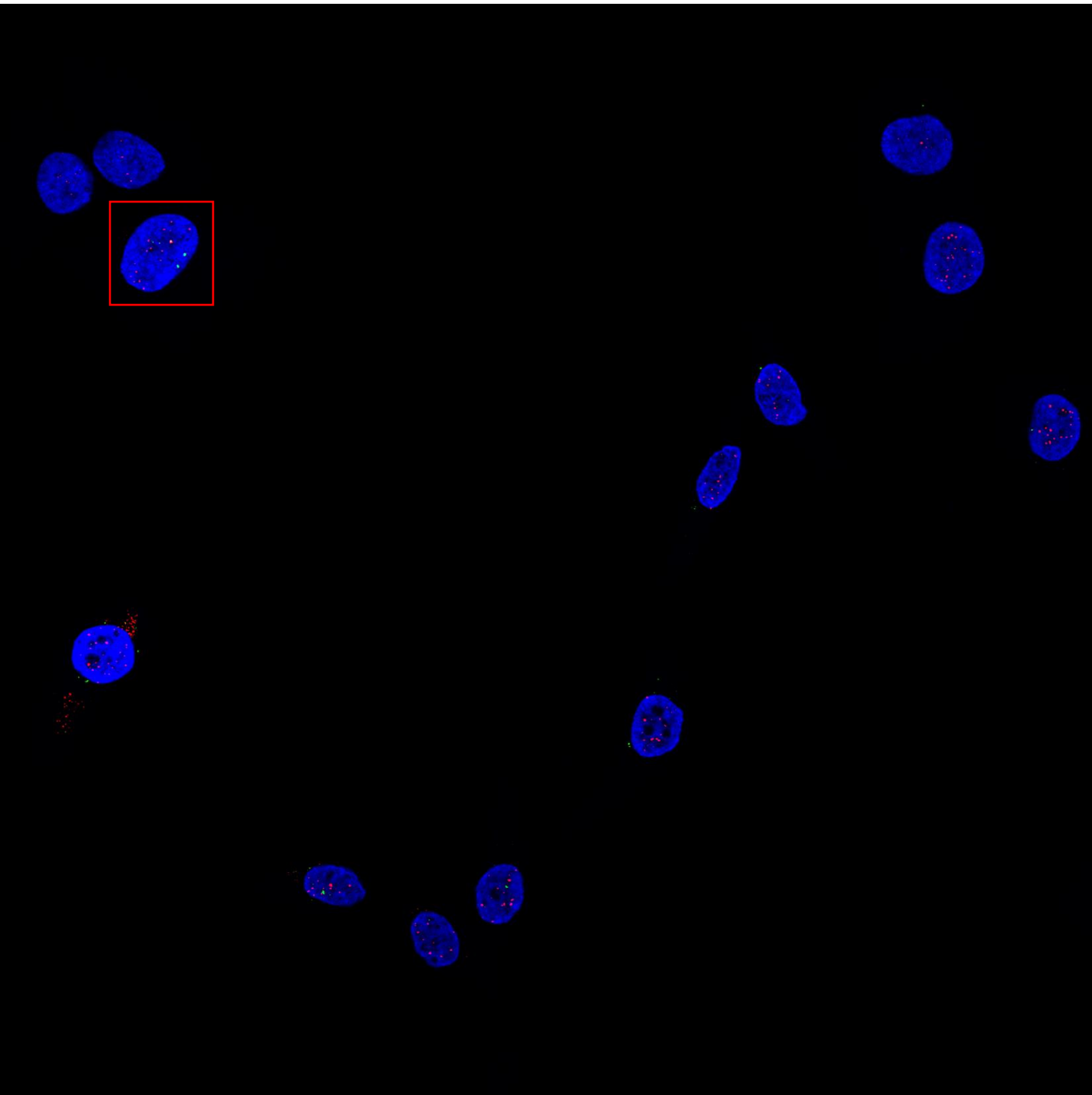

Fig 3E

TERRA OE

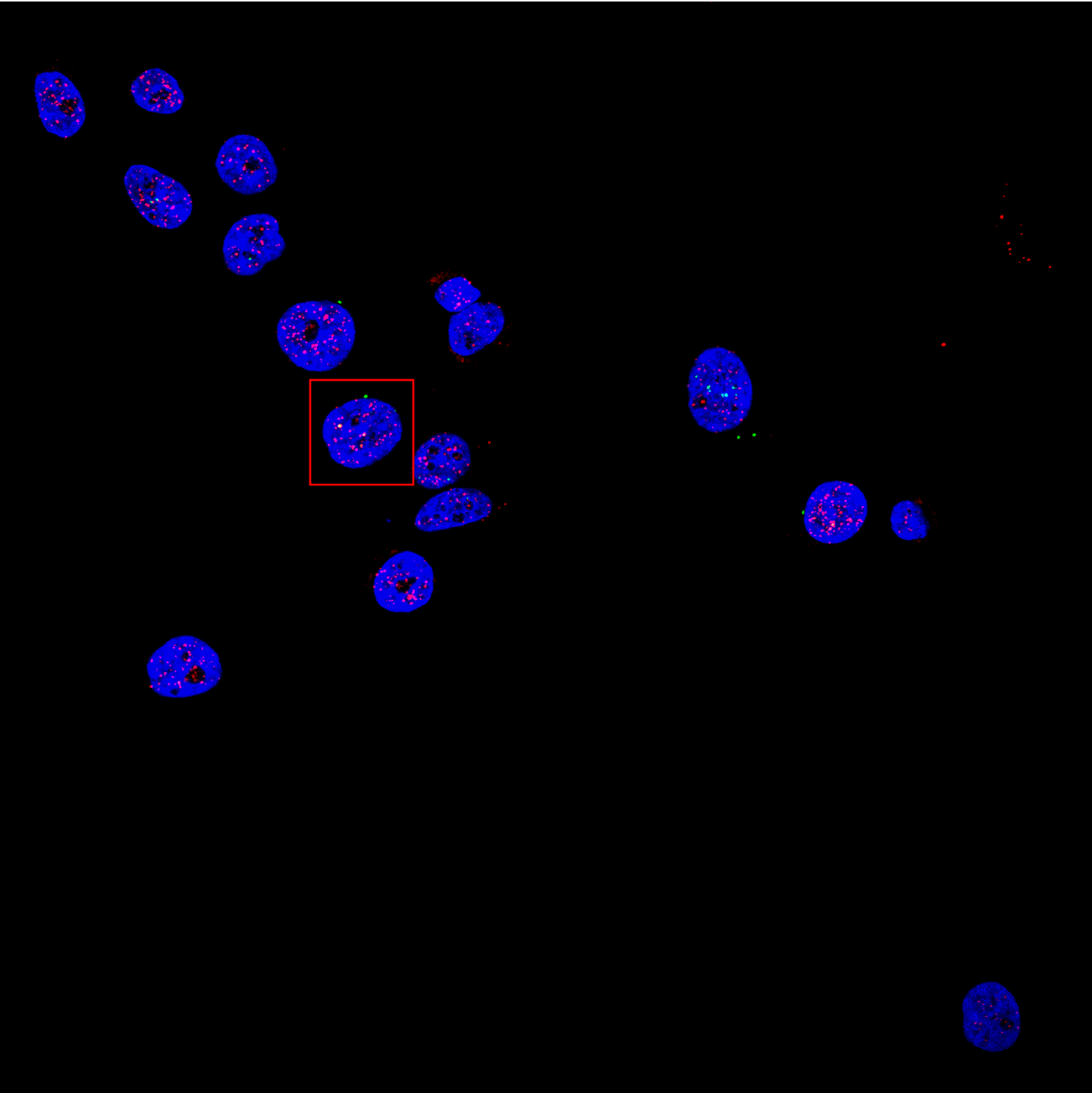

Fig 3E

U2OS

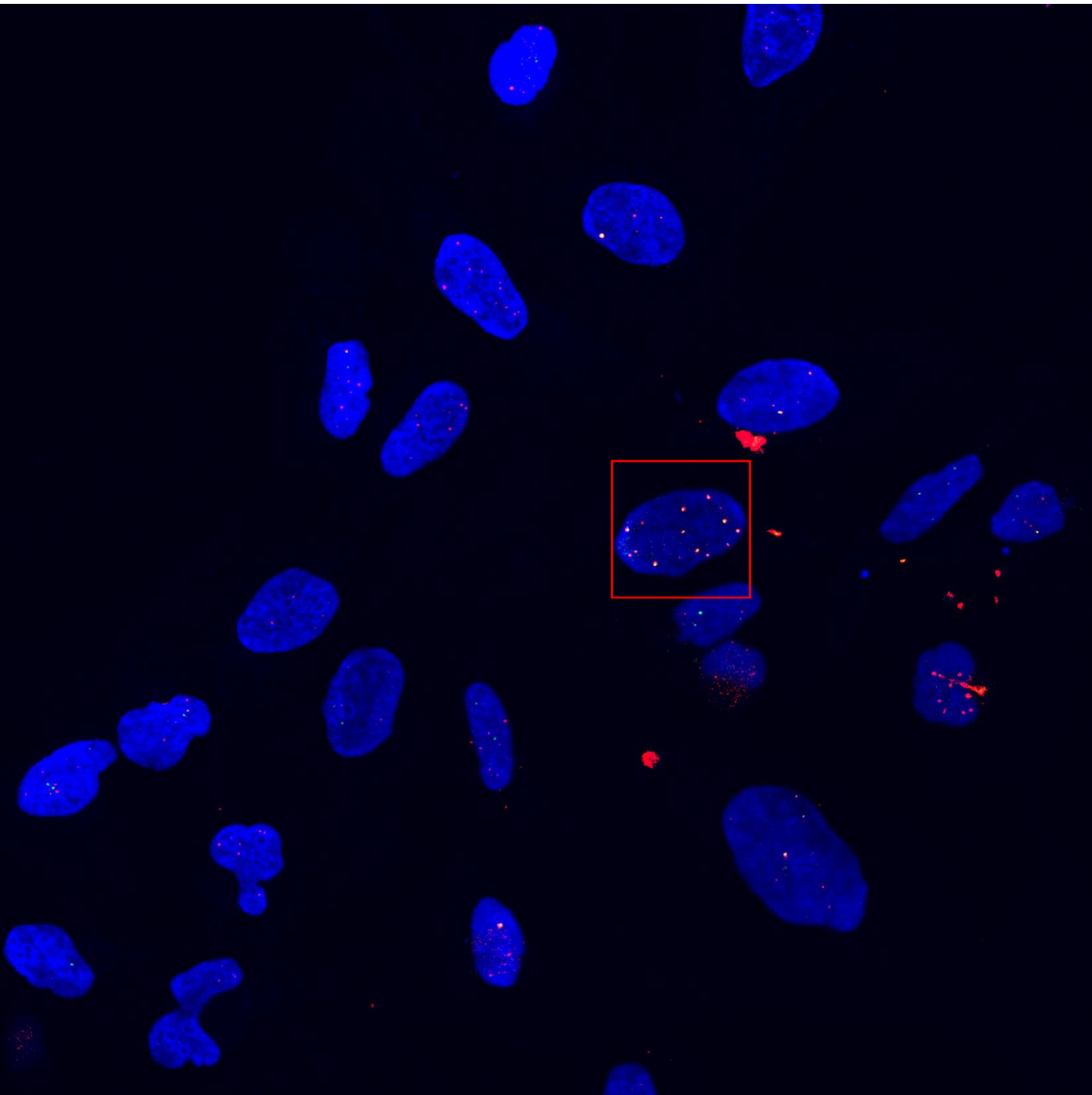

Supplement: Supplementary file 4 — Source data Fig. 3 [file 44318_2025_502_MOESM4_ESM.zip › Figure 3/Fig 3E/Fig 3E.pdf]

Fig 3A

Ctrl, Untreated

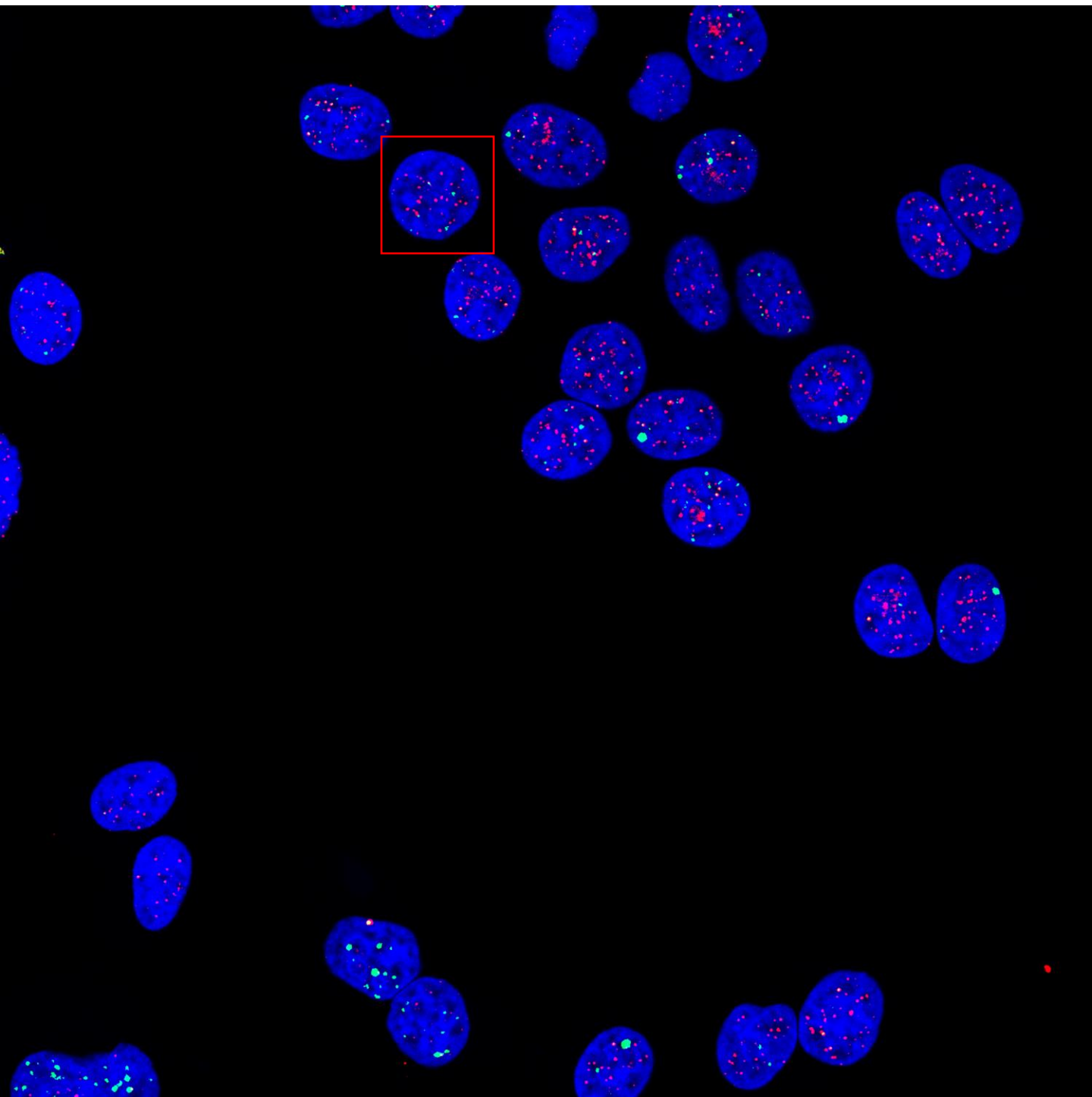

Fig 3A

Ctrl, Zeocin

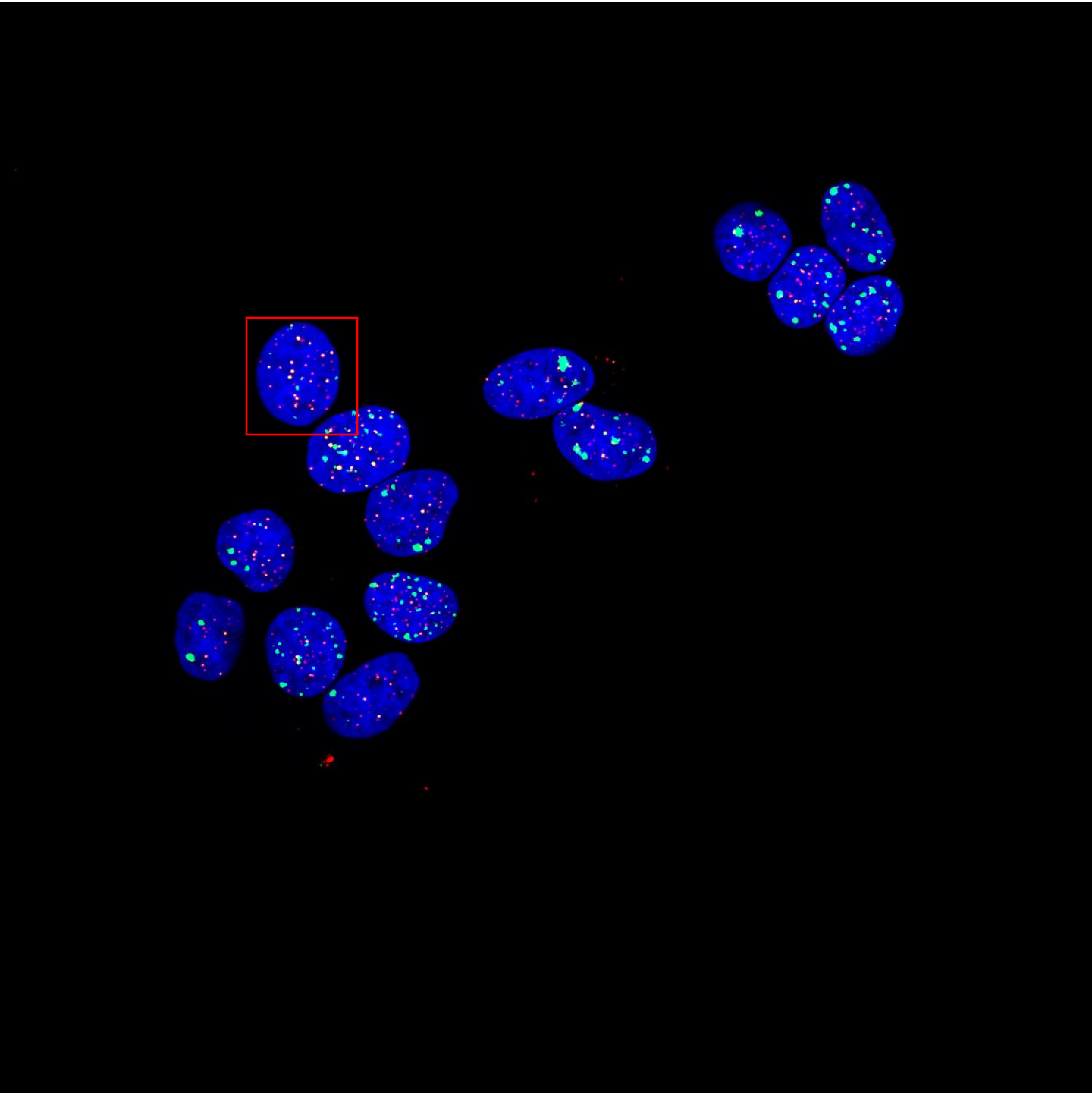

Fig 3A

TERRA OE, Untreated

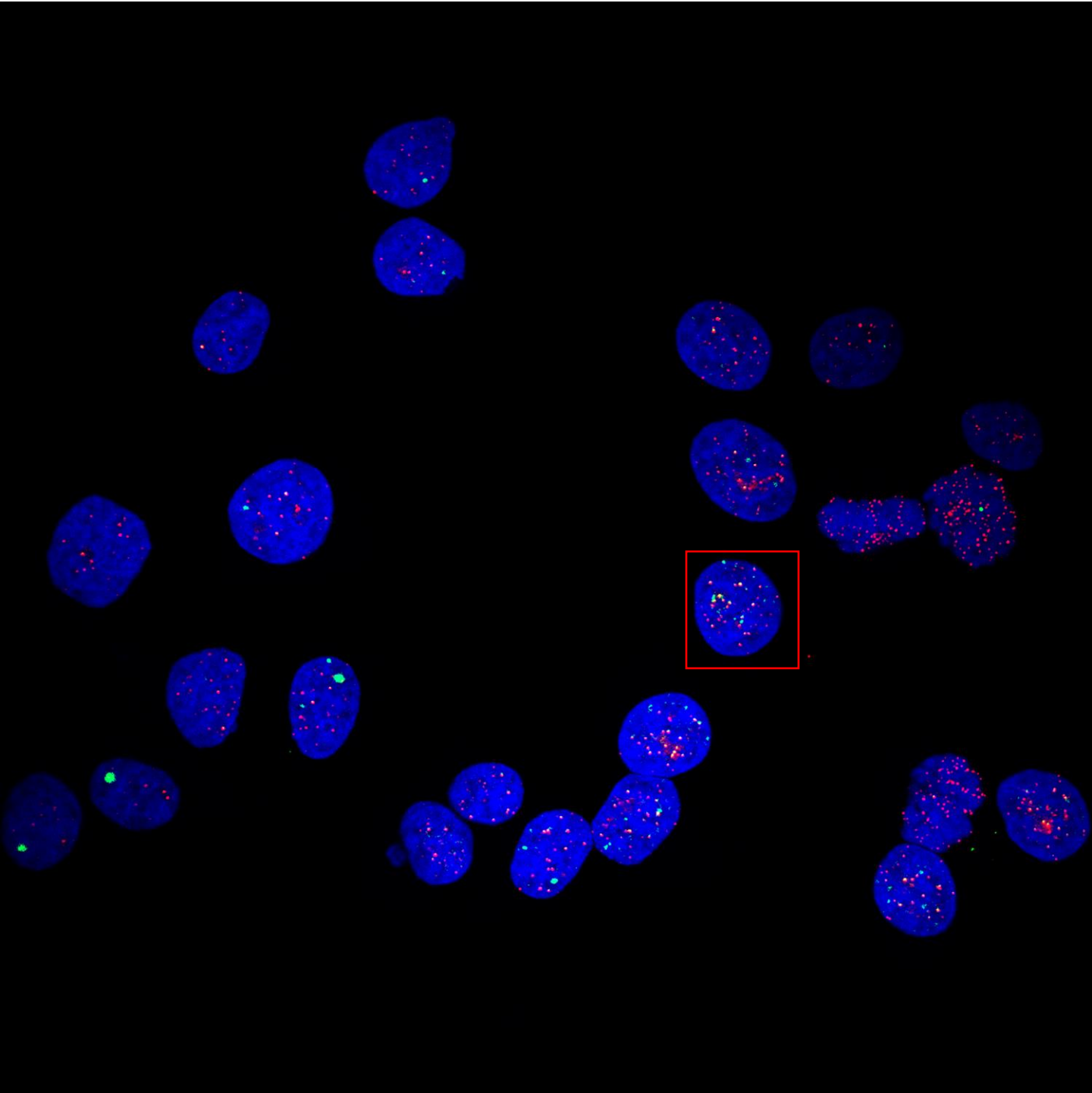

Fig 3A

TERRA OE, Zeocin

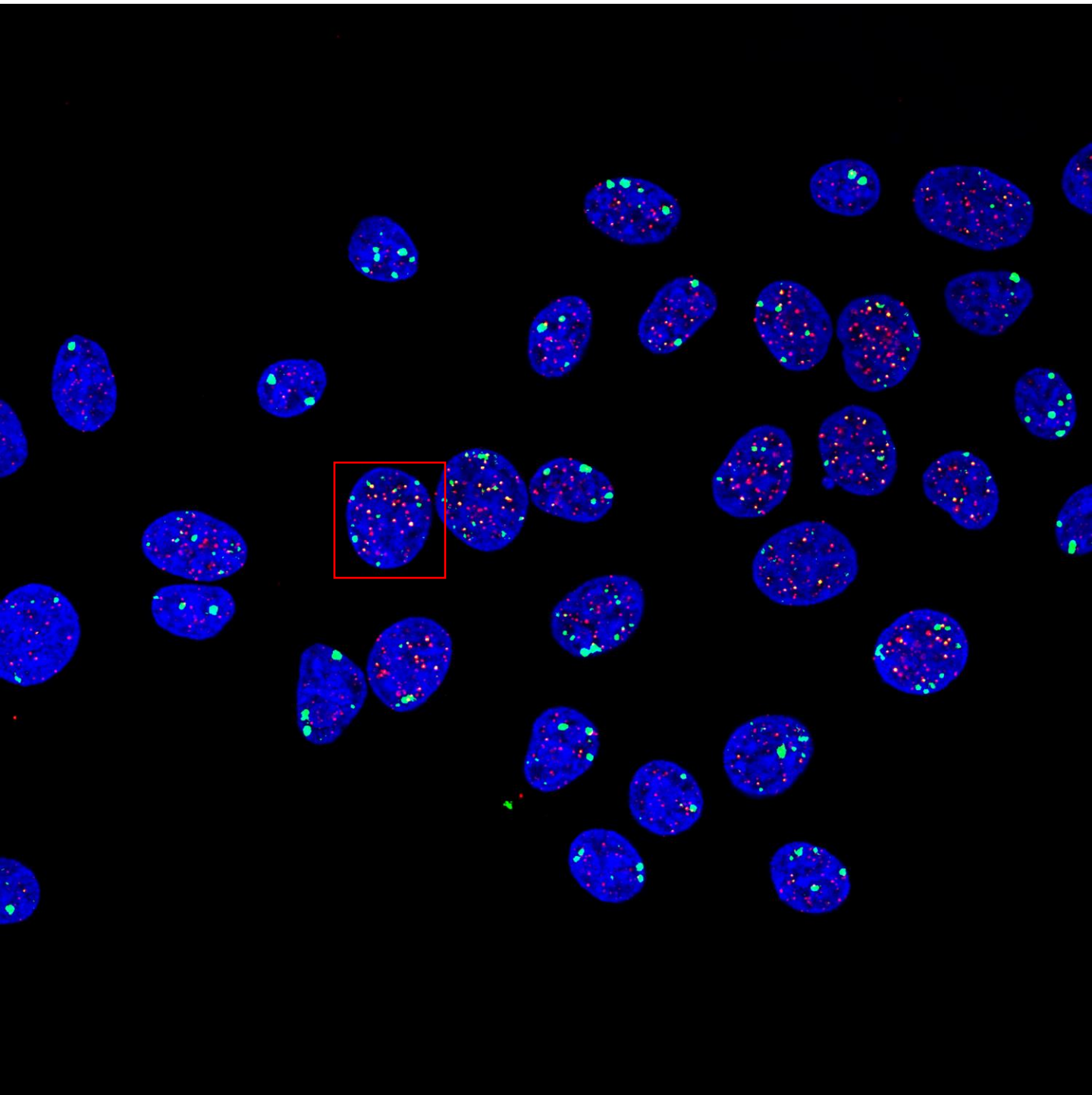

Supplement: Supplementary file 4 — Source data Fig. 3 [file 44318_2025_502_MOESM4_ESM.zip › Figure 3/Fig 3A/Fig 3A.pdf]

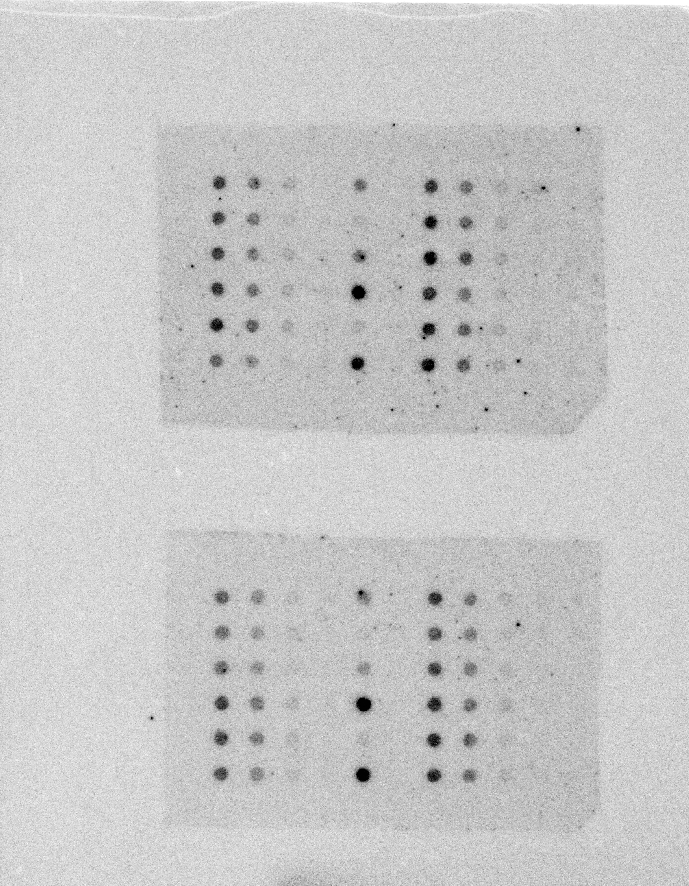

Supplement: Supplementary file 5 — Source data Fig. 4 [file 44318_2025_502_MOESM5_ESM.zip › Figure 4/Fig 4C/Fig 4C.jpg]

Fig 4C

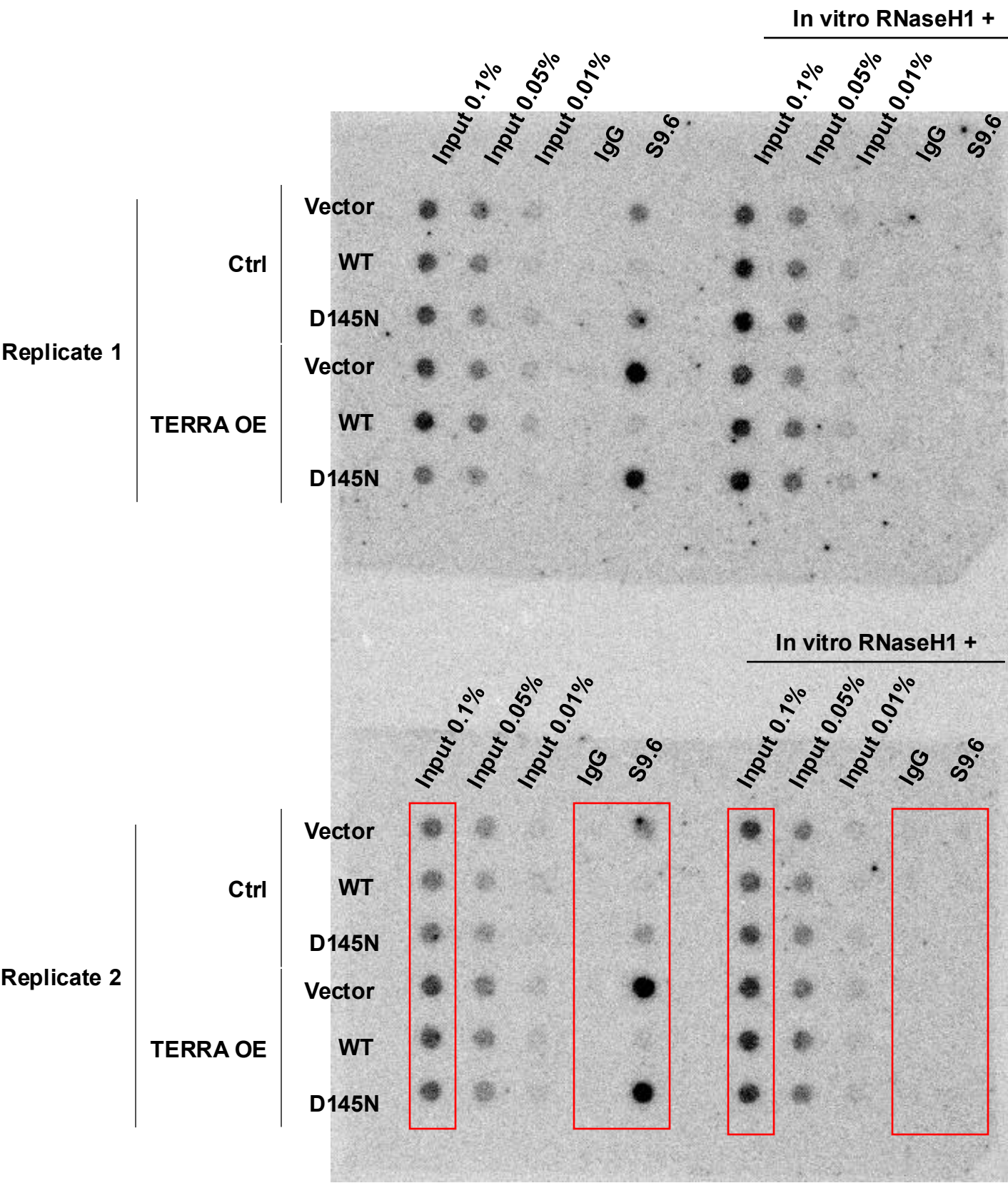

Supplement: Supplementary file 5 — Source data Fig. 4 [file 44318_2025_502_MOESM5_ESM.zip › Figure 4/Fig 4C/Fig 4C.pdf]

Fig 4A

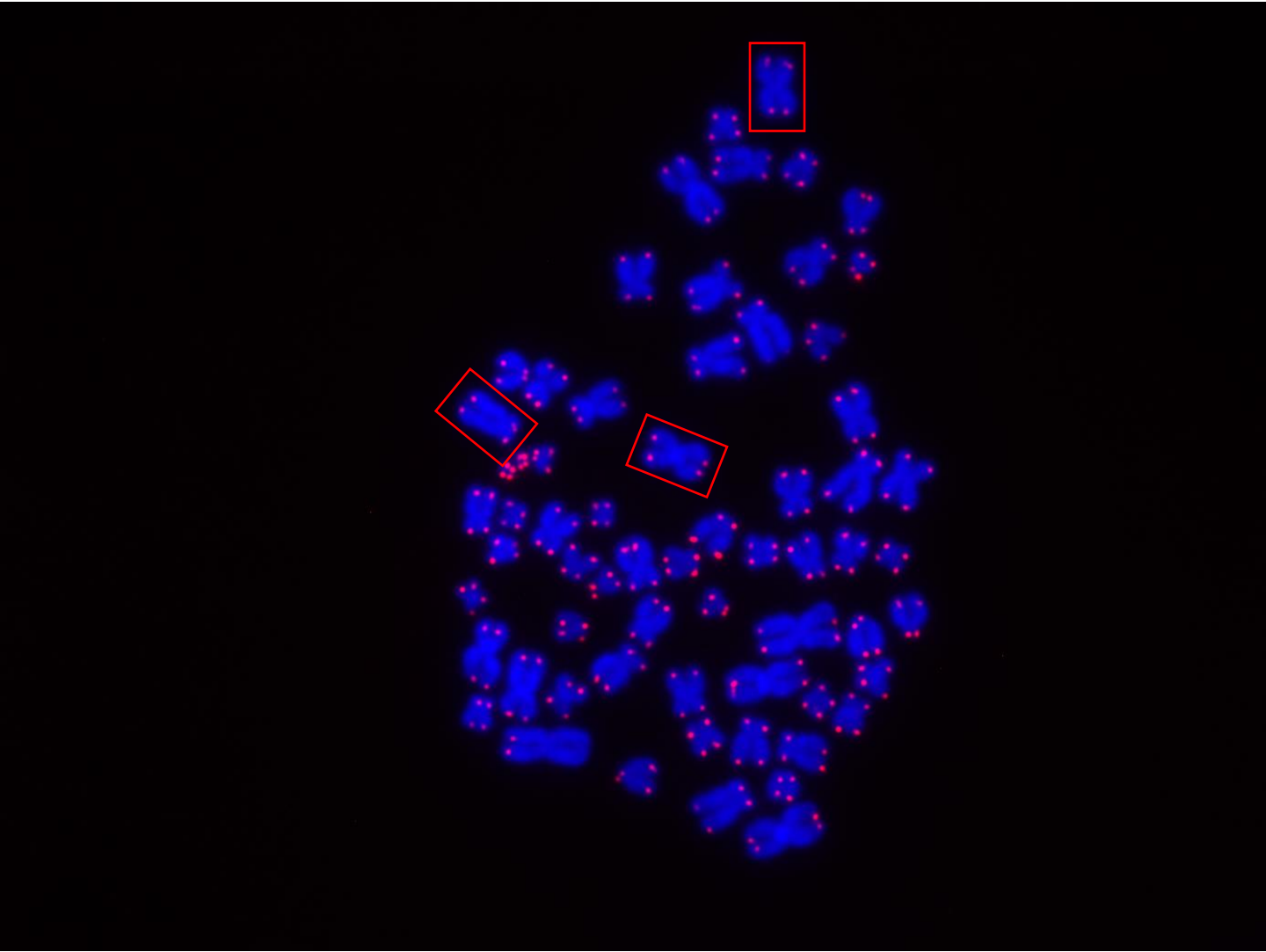

Supplement: Supplementary file 5 — Source data Fig. 4 [file 44318_2025_502_MOESM5_ESM.zip › Figure 4/Fig 4A/Fig 4A.pdf]

Fig 4F

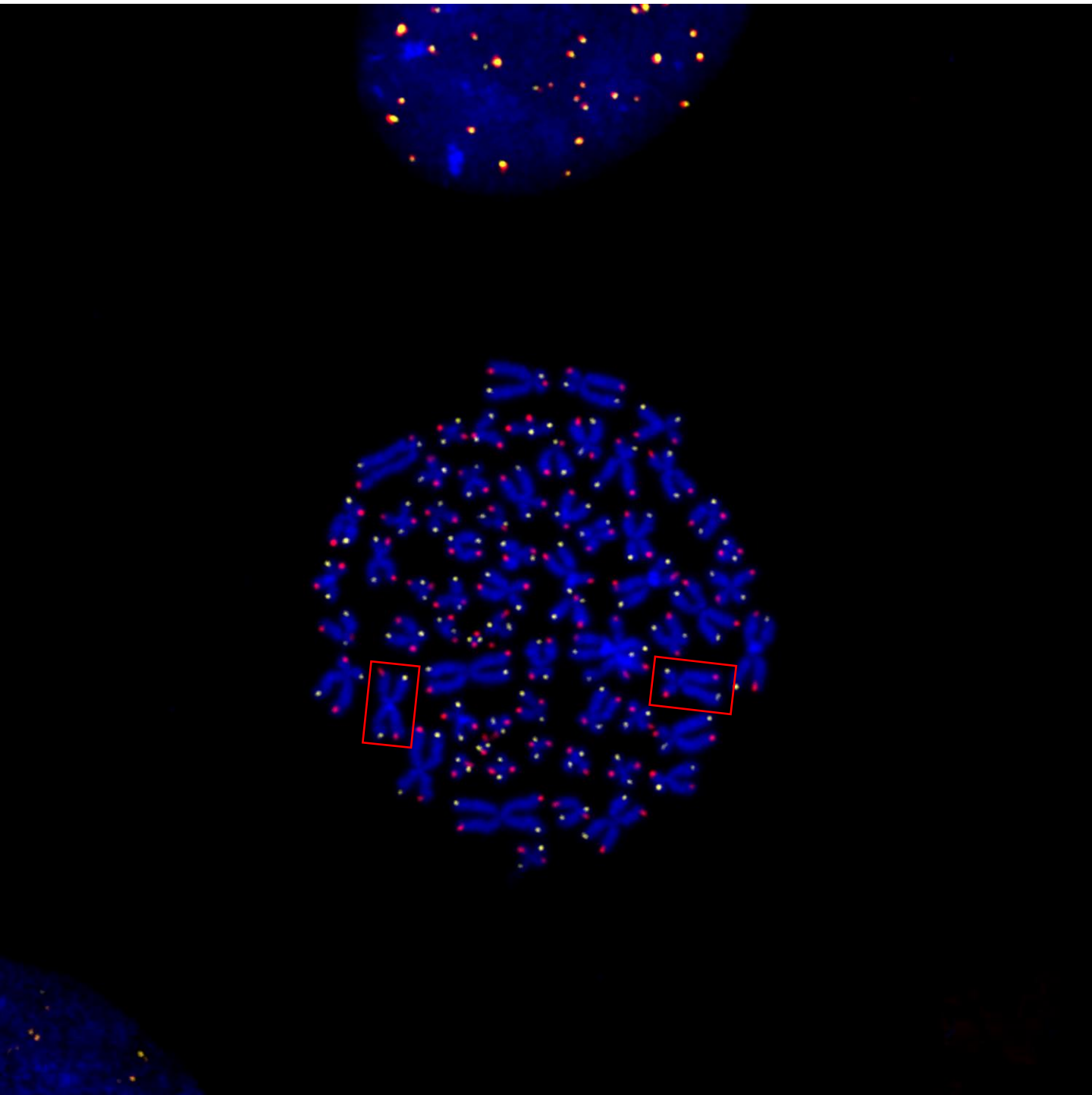

Supplement: Supplementary file 5 — Source data Fig. 4 [file 44318_2025_502_MOESM5_ESM.zip › Figure 4/Fig 4F/Fig 4F.pdf]

Fig 6D

Ctrl

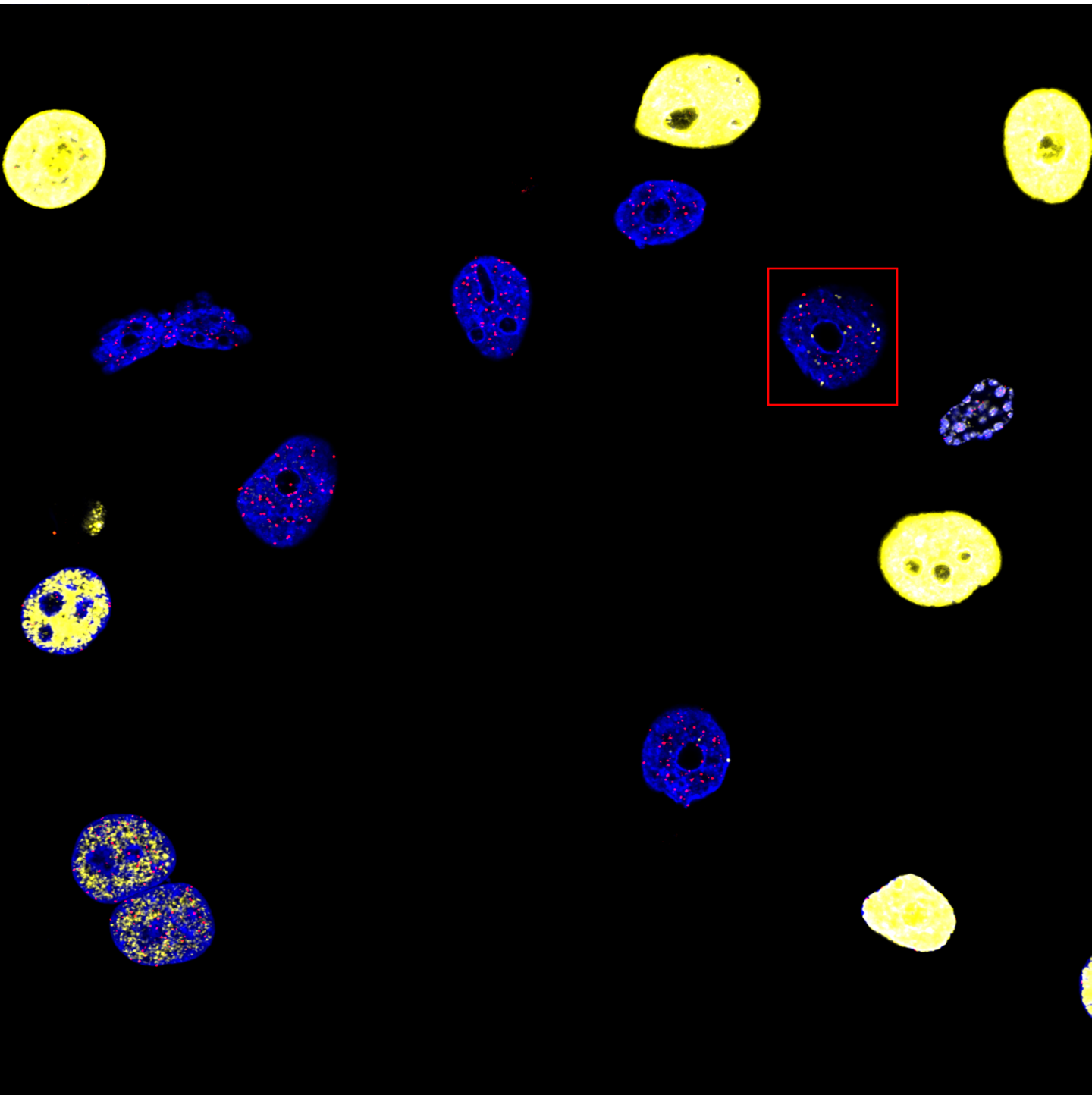

Fig 6D

TERRA OE

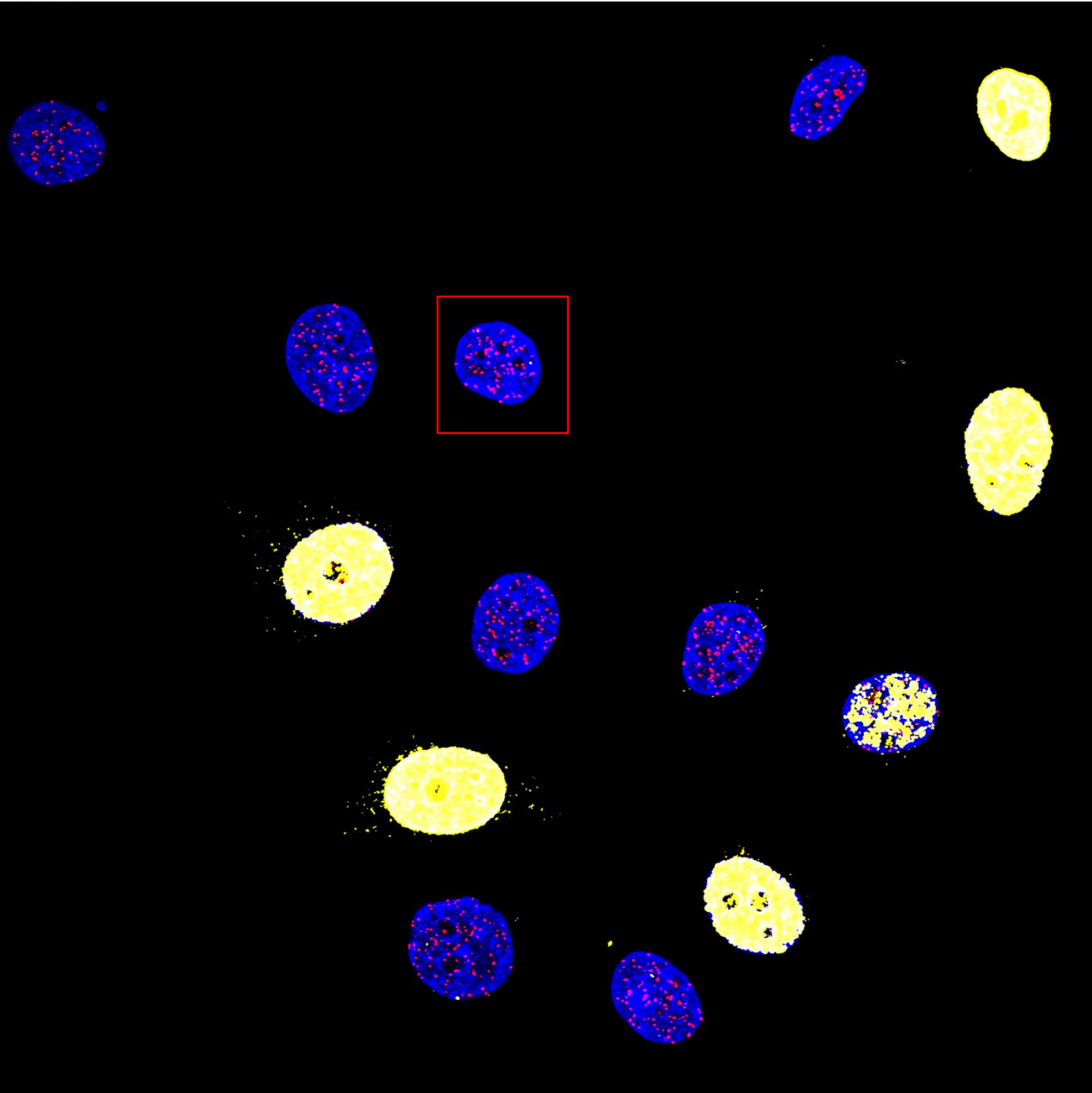

Fig 6D

U2OS

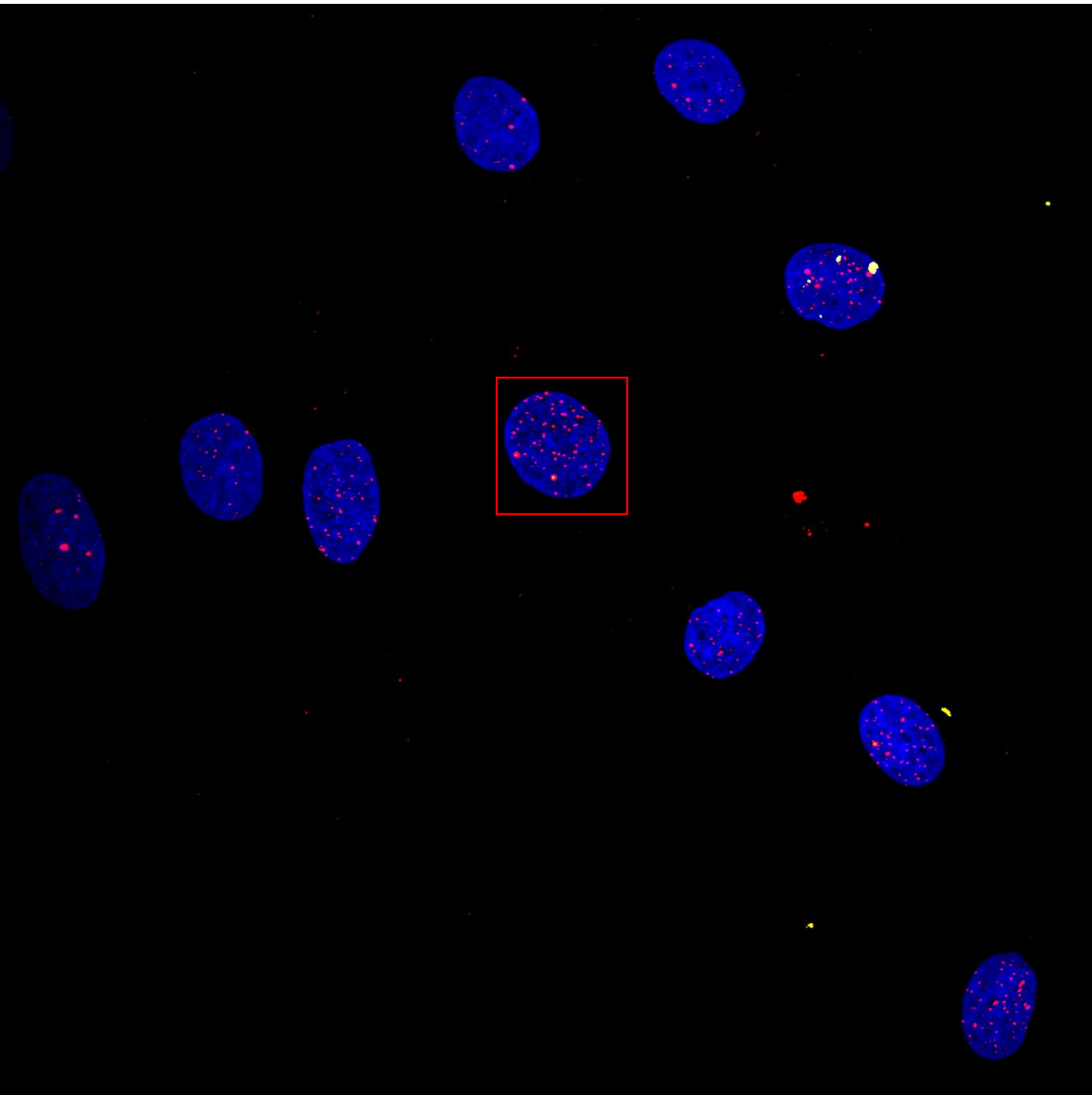

Supplement: Supplementary file 7 — Source data Fig. 6 [file 44318_2025_502_MOESM7_ESM.zip › Figure 6/Fig 6D/Fig 6D.pdf]

Fig 6B (Left)

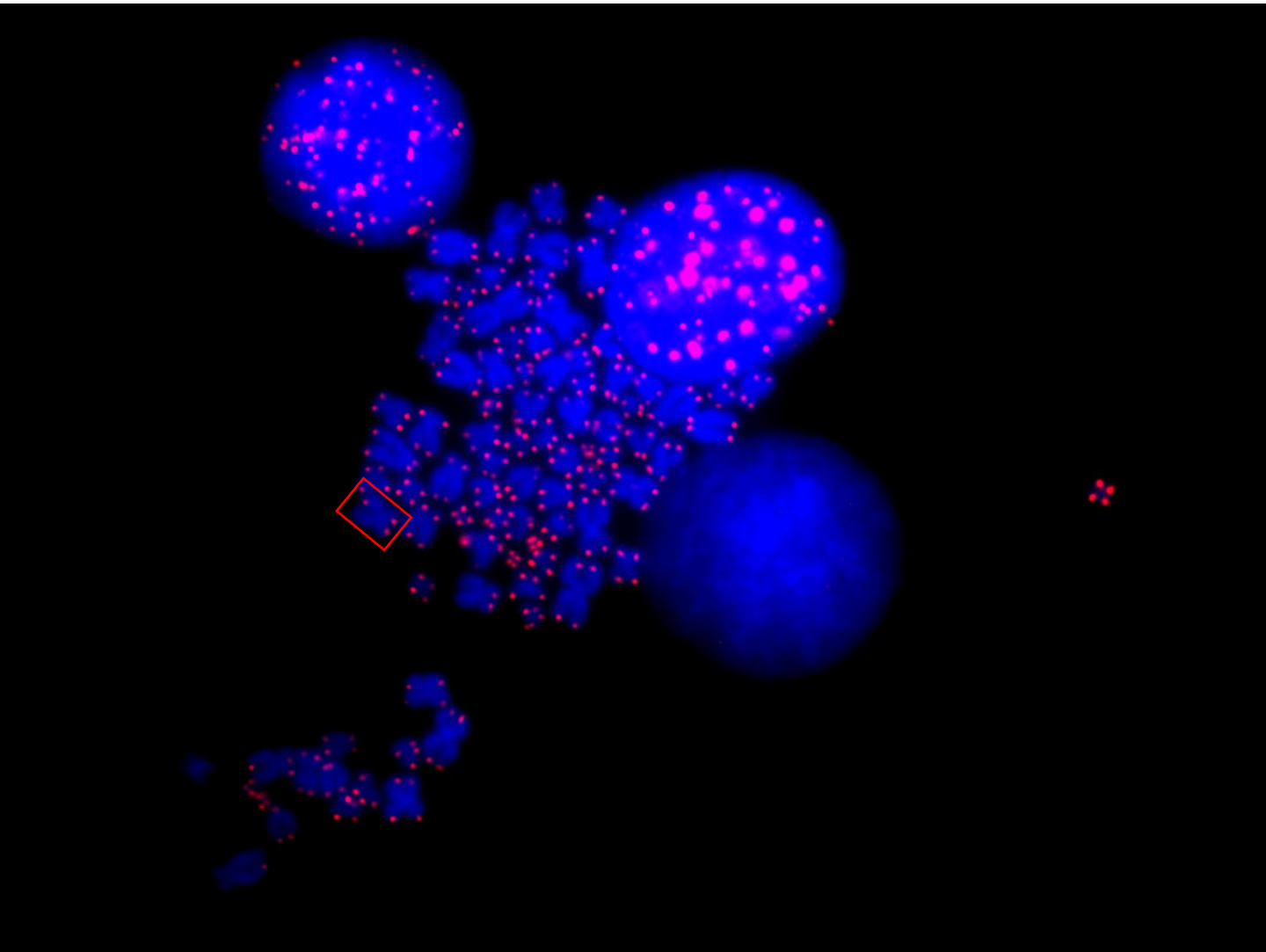

Fig 6B (Middle)

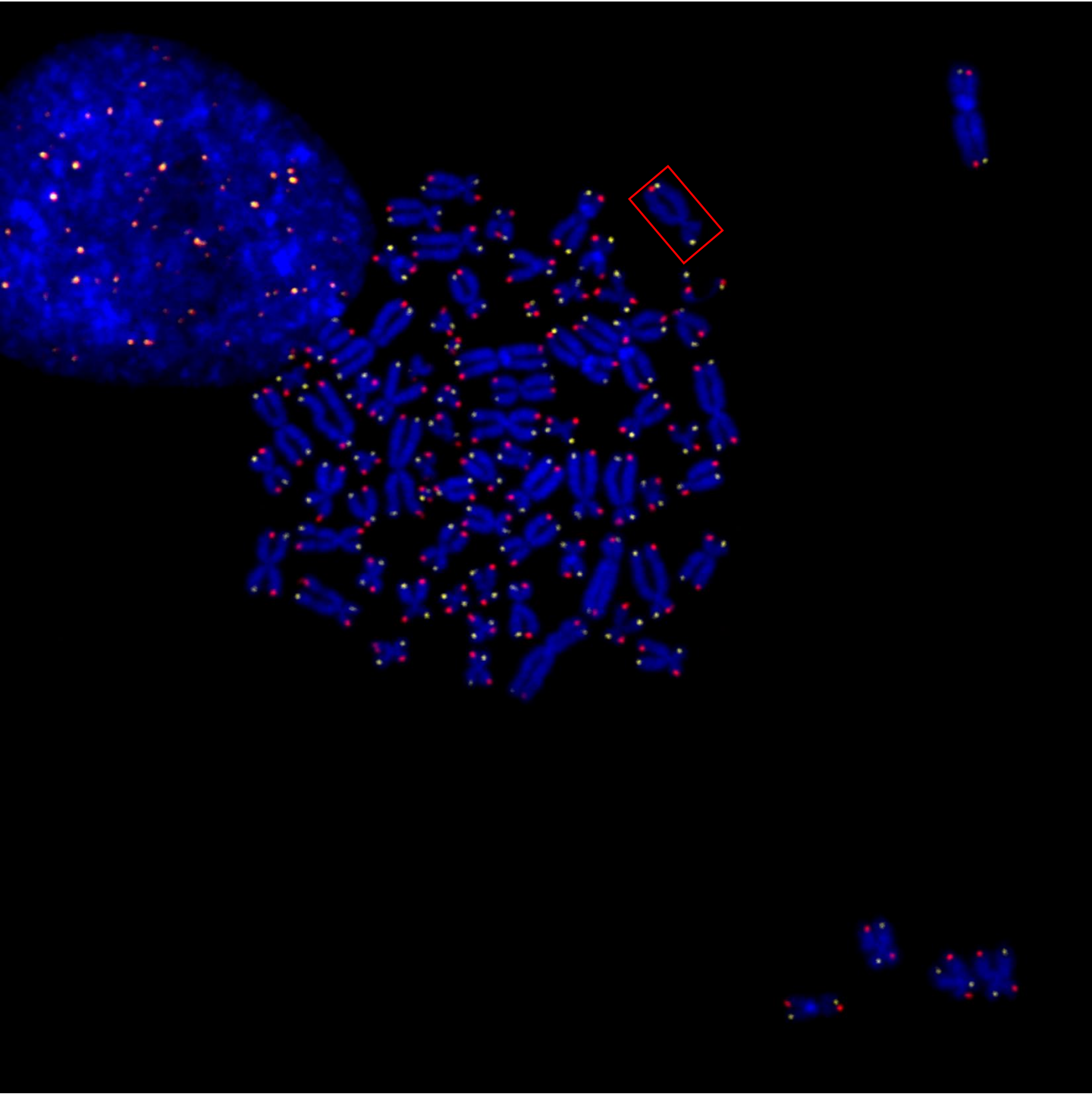

Fig 6B (Right)

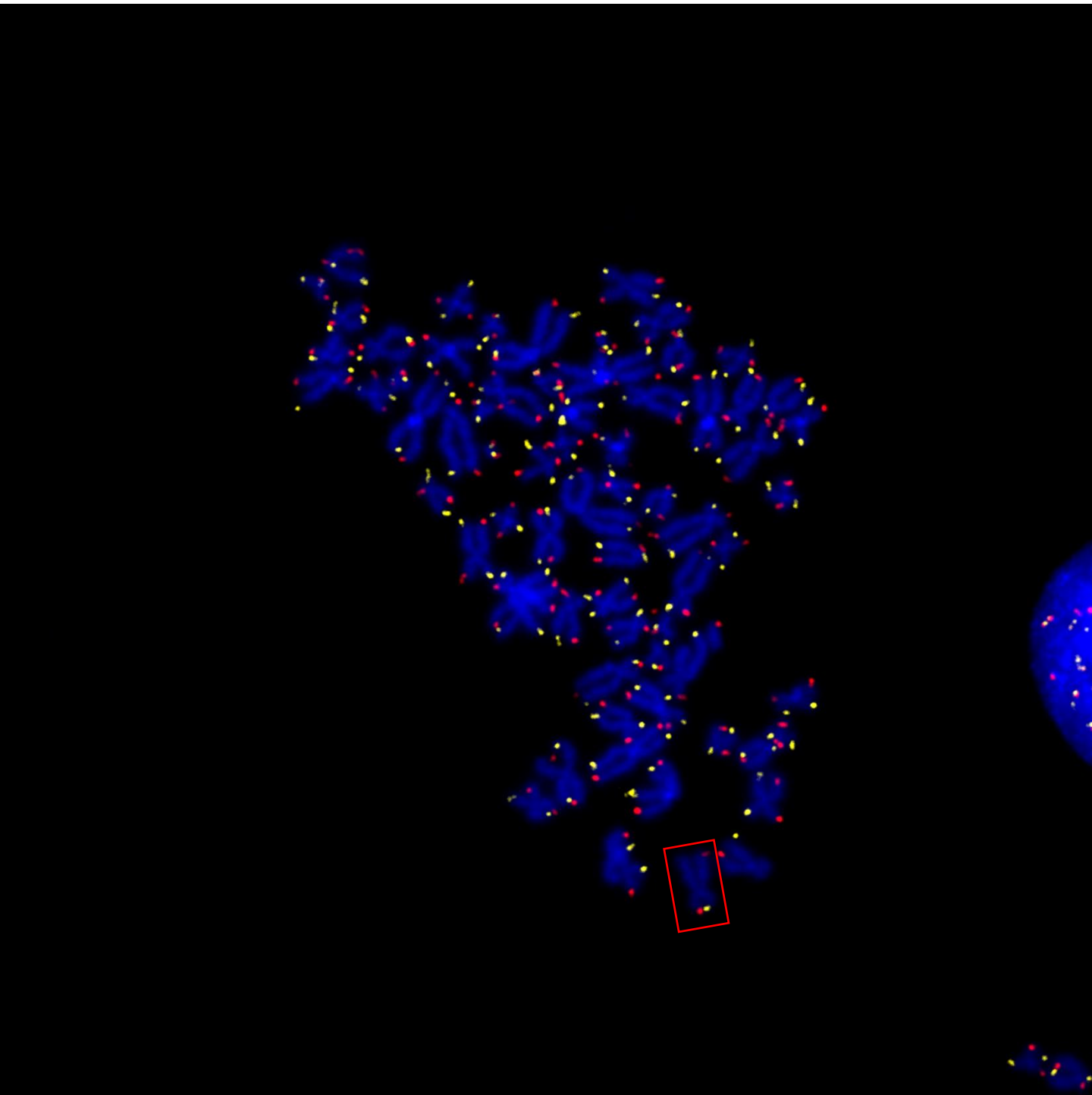

Supplement: Supplementary file 7 — Source data Fig. 6 [file 44318_2025_502_MOESM7_ESM.zip › Figure 6/Fig 6B/Fig 6B.pdf]

Fig EV1B

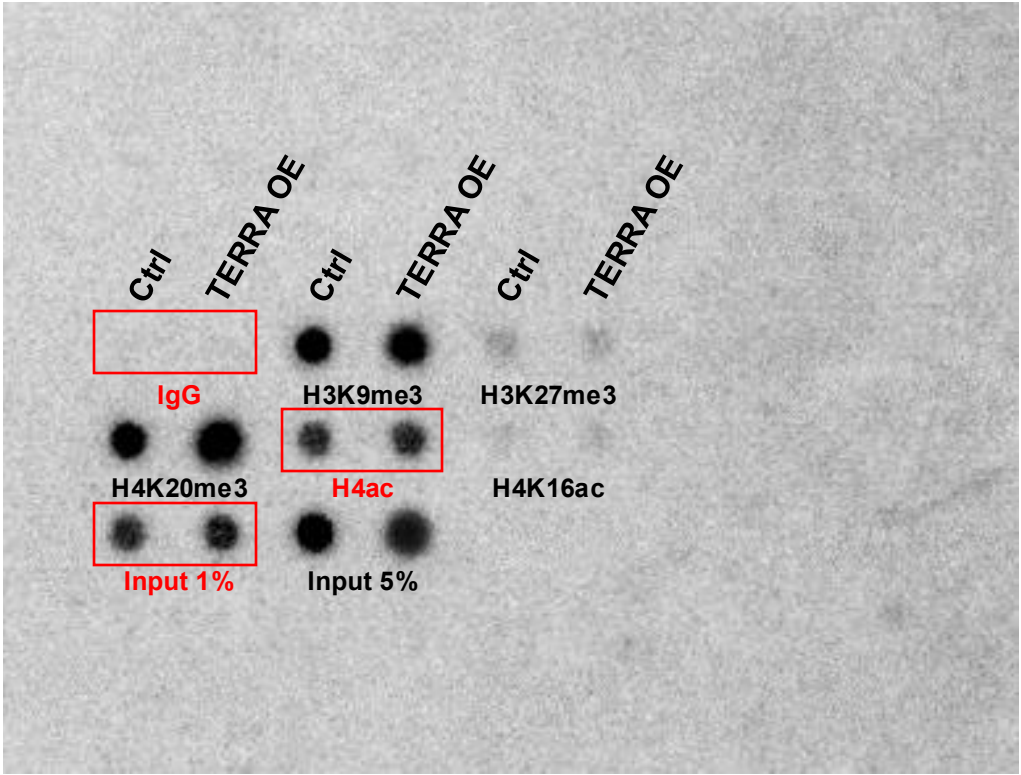

Supplement: Supplementary file 9 — Figure EV1 Source Data [file 44318_2025_502_MOESM9_ESM.zip › Figure EV1/Fig EV1B/Fig EV1B.pdf]

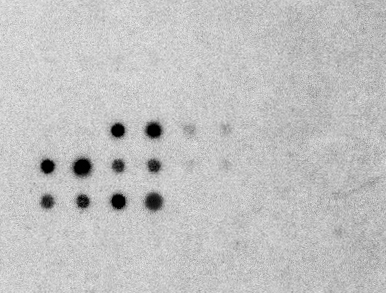

Supplement: Supplementary file 9 — Figure EV1 Source Data [file 44318_2025_502_MOESM9_ESM.zip › Figure EV1/Fig EV1B/Fig EV1B.jpg]

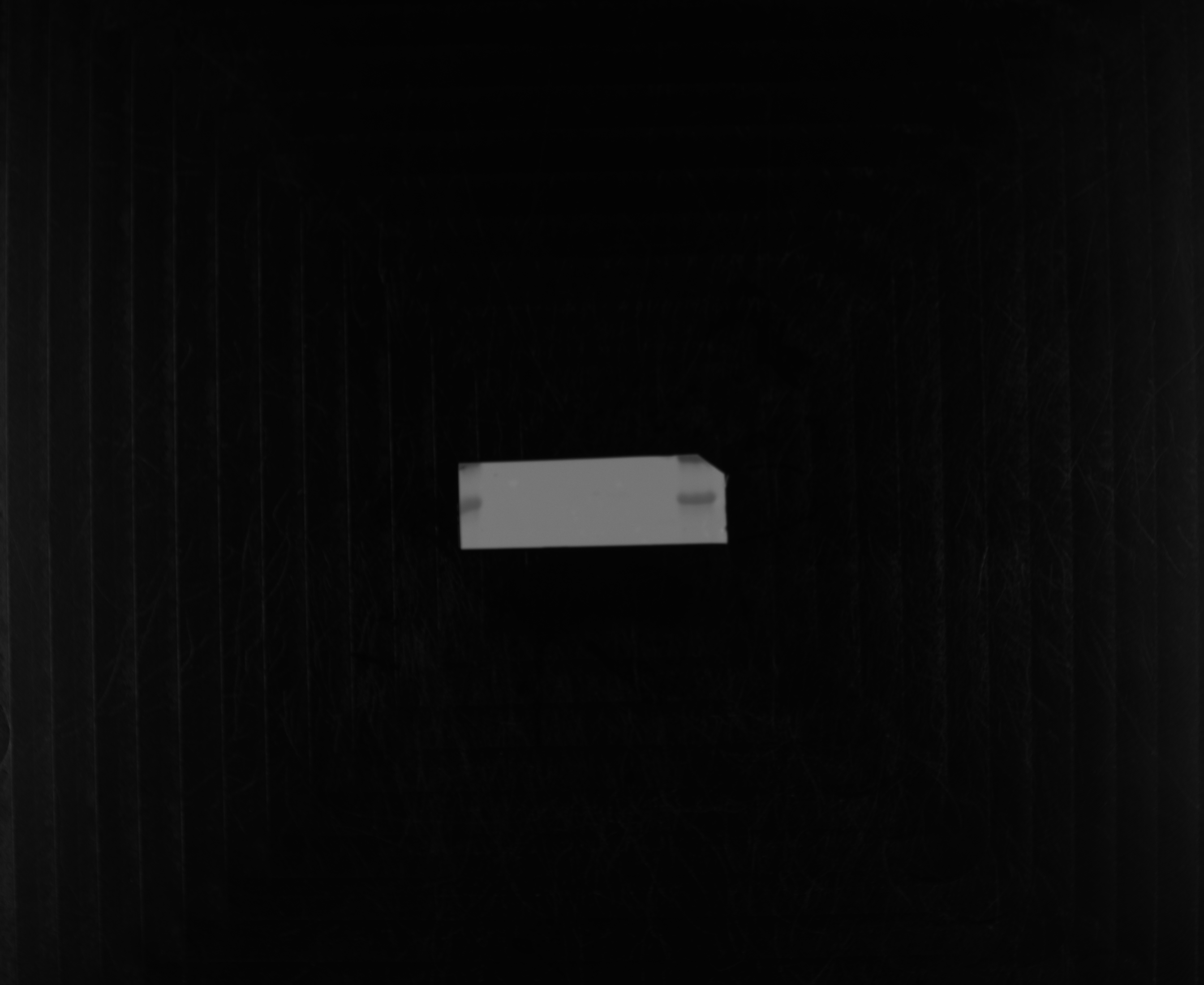

Supplement: Supplementary file 10 — Figure EV2 Source Data [file 44318_2025_502_MOESM10_ESM.zip › Figure EV2/Fig EV2C/Tubulin - marker.Tif]

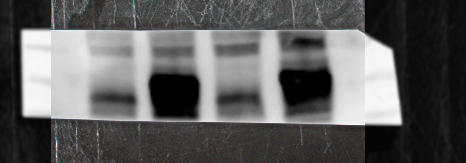

Supplement: Supplementary file 10 — Figure EV2 Source Data [file 44318_2025_502_MOESM10_ESM.zip › Figure EV2/Fig EV2C/RNaseH1 - merge.jpg]

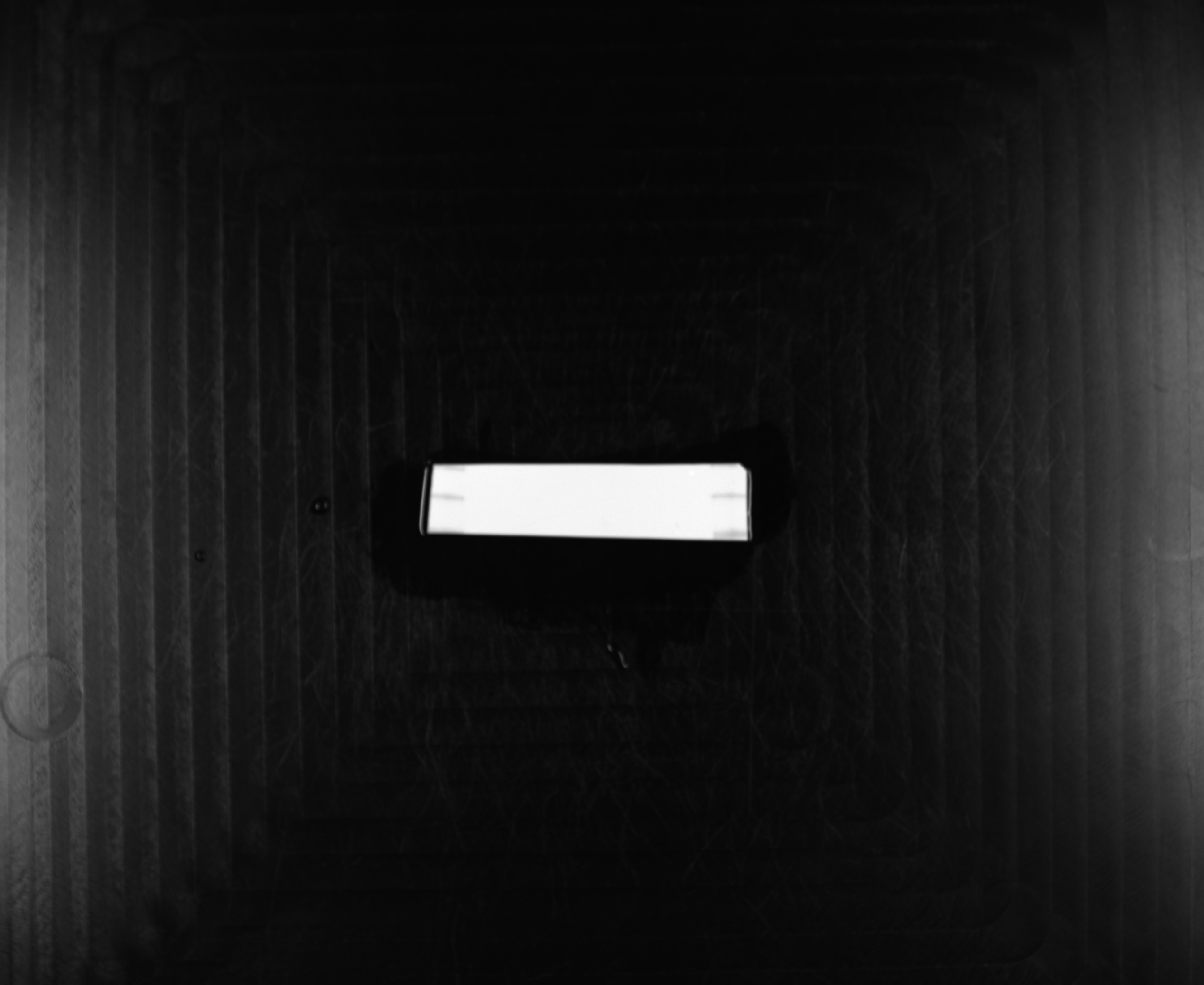

Supplement: Supplementary file 10 — Figure EV2 Source Data [file 44318_2025_502_MOESM10_ESM.zip › Figure EV2/Fig EV2C/Vinculin - marker.Tif]

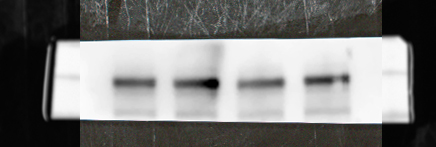

Supplement: Supplementary file 10 — Figure EV2 Source Data [file 44318_2025_502_MOESM10_ESM.zip › Figure EV2/Fig EV2C/Vinculin - merge.jpg]

Fig EV2C

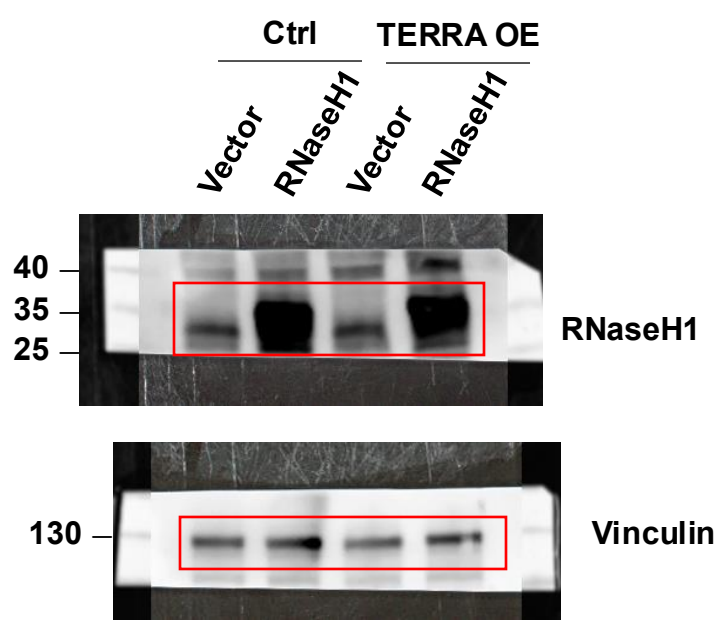

Supplement: Supplementary file 10 — Figure EV2 Source Data [file 44318_2025_502_MOESM10_ESM.zip › Figure EV2/Fig EV2C/Fig EV2C.pdf]

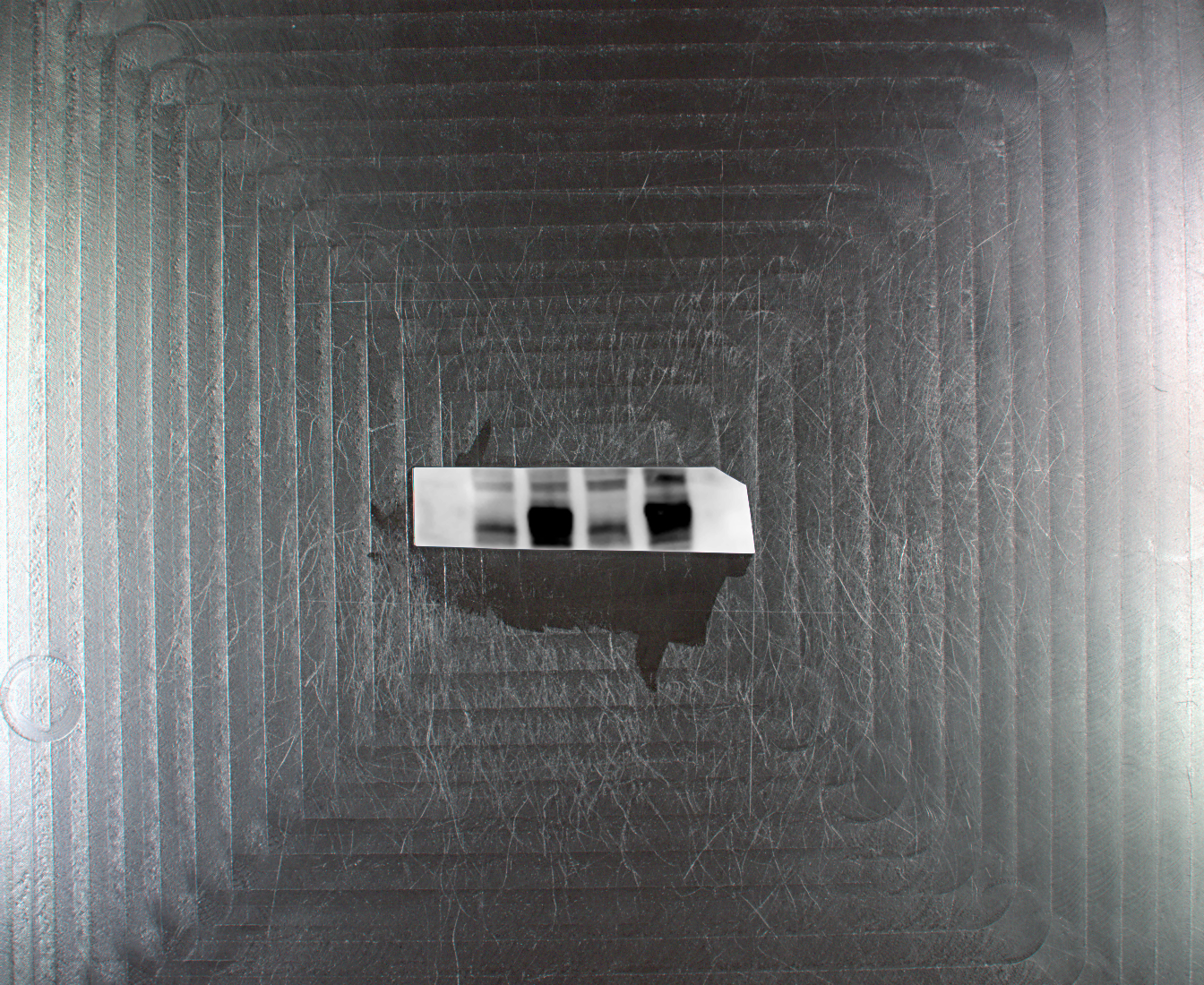

Supplement: Supplementary file 10 — Figure EV2 Source Data [file 44318_2025_502_MOESM10_ESM.zip › Figure EV2/Fig EV2C/RNaseH1.Tif]

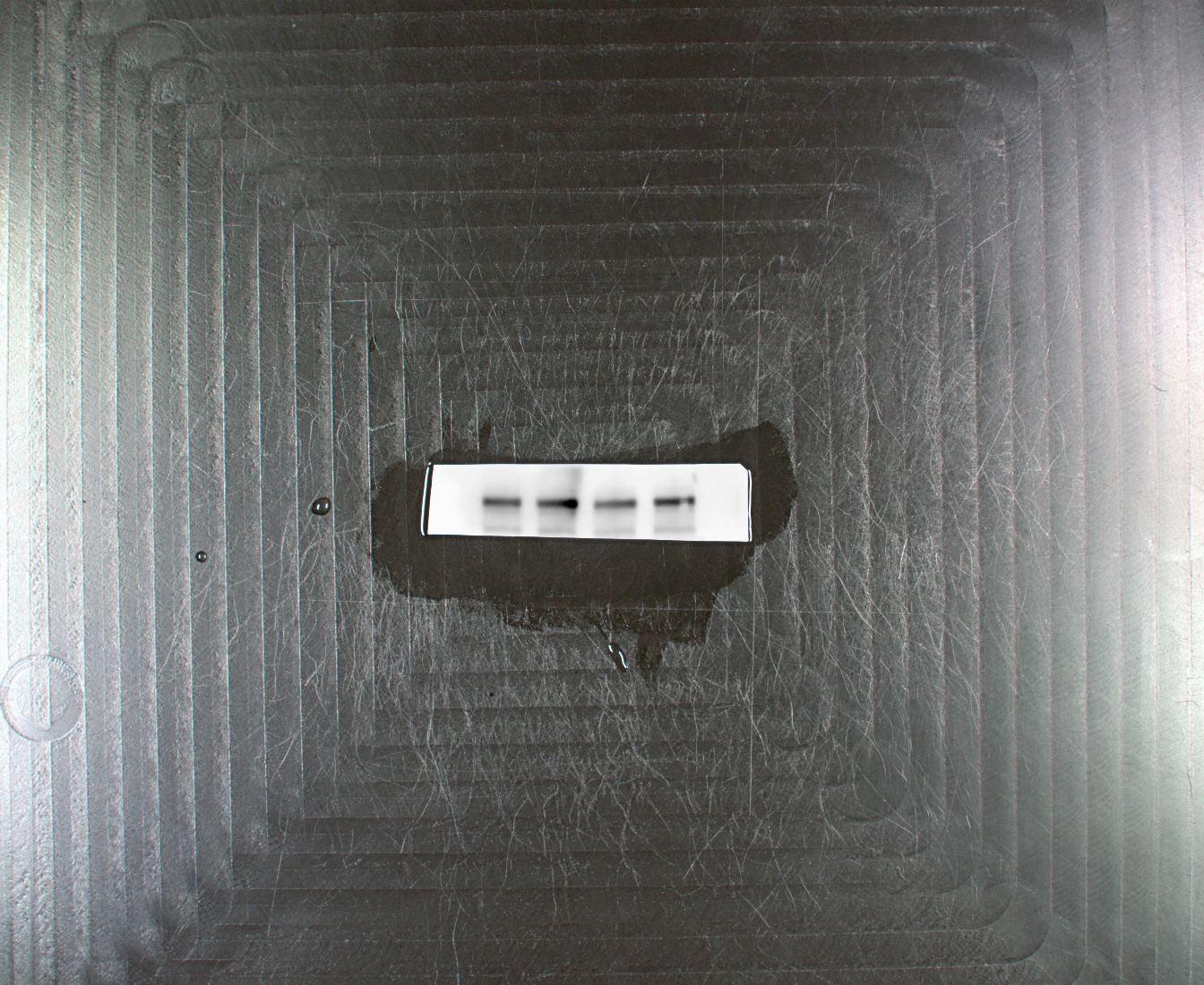

Supplement: Supplementary file 10 — Figure EV2 Source Data [file 44318_2025_502_MOESM10_ESM.zip › Figure EV2/Fig EV2C/Vinculin.Tif]

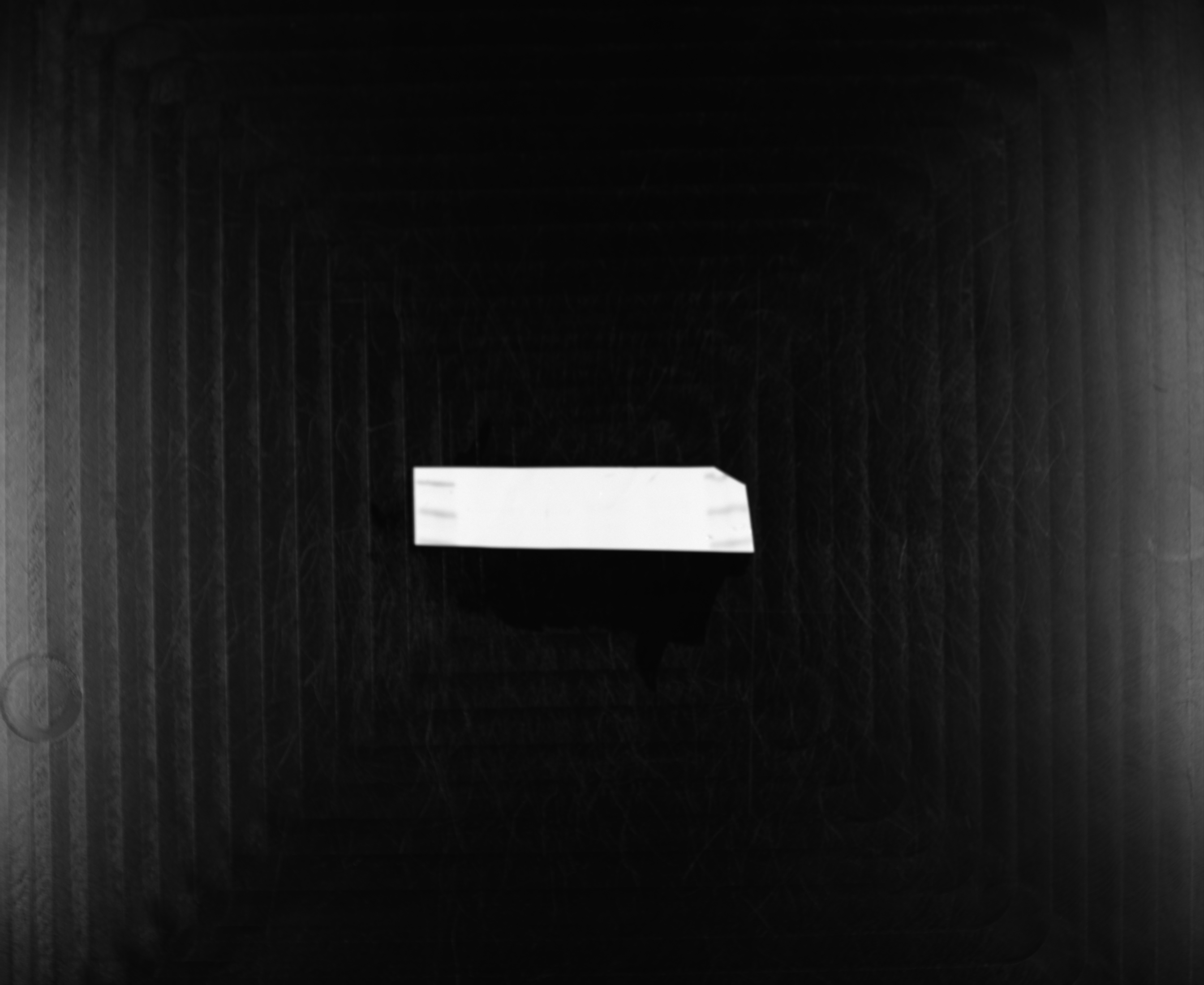

Supplement: Supplementary file 10 — Figure EV2 Source Data [file 44318_2025_502_MOESM10_ESM.zip › Figure EV2/Fig EV2C/RNaseH1 - marker.Tif]

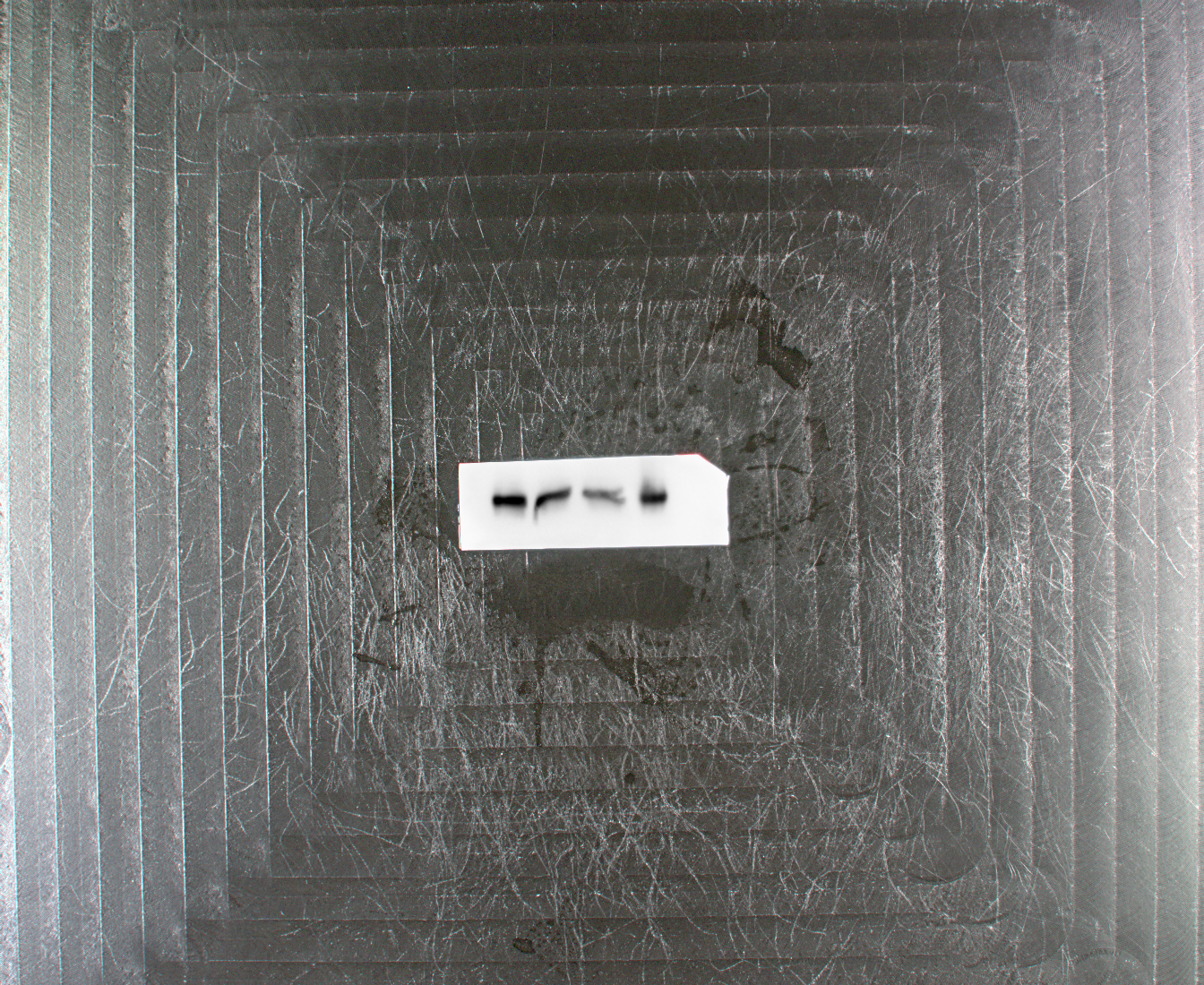

Supplement: Supplementary file 10 — Figure EV2 Source Data [file 44318_2025_502_MOESM10_ESM.zip › Figure EV2/Fig EV2C/Tubulin - 1 sec.Tif]

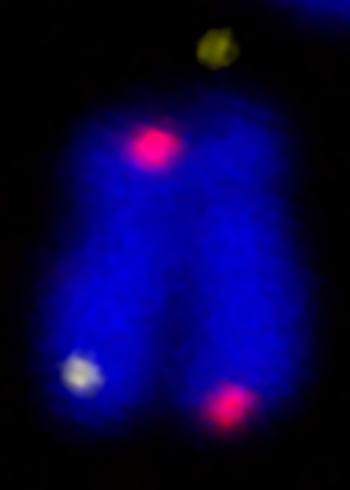

Supplement: Supplementary file 11 — Figure EV3 Source Data [file 44318_2025_502_MOESM11_ESM.zip › Figure EV3/Fig EV3F/Outsider (Leading).tif]

Fig EV3F

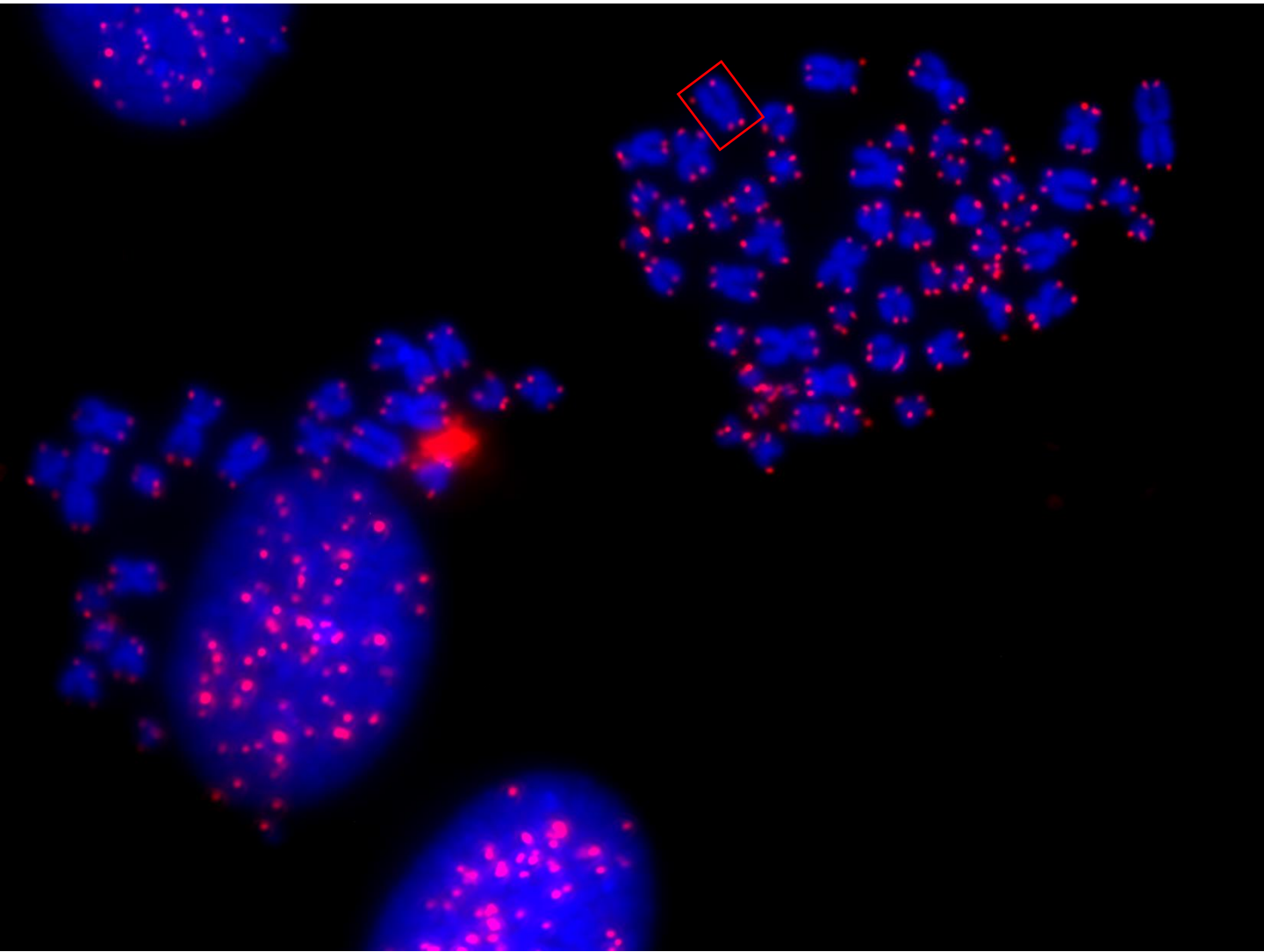

Fig EV3F

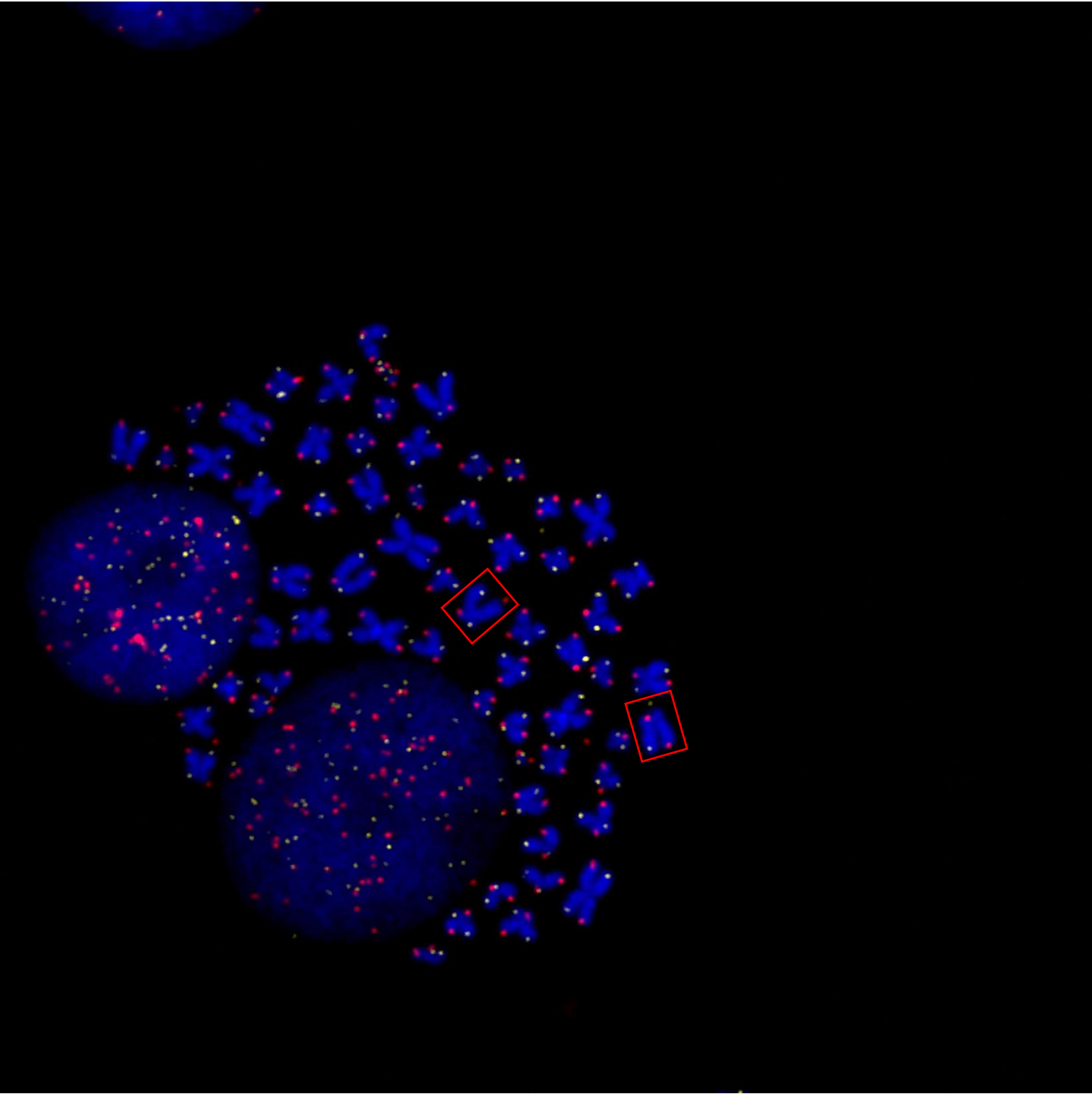

Supplement: Supplementary file 11 — Figure EV3 Source Data [file 44318_2025_502_MOESM11_ESM.zip › Figure EV3/Fig EV3F/Fig EV3F.pdf]

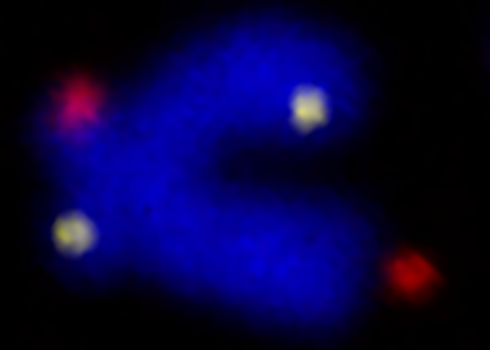

Supplement: Supplementary file 11 — Figure EV3 Source Data [file 44318_2025_502_MOESM11_ESM.zip › Figure EV3/Fig EV3F/Outsider (Lagging).tif]

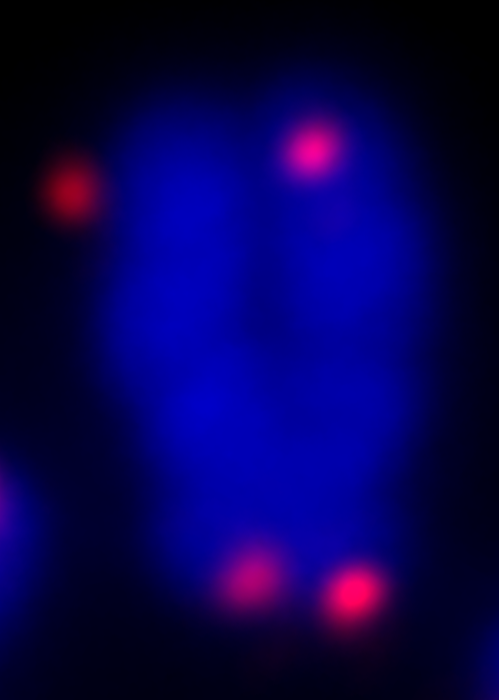

Supplement: Supplementary file 11 — Figure EV3 Source Data [file 44318_2025_502_MOESM11_ESM.zip › Figure EV3/Fig EV3F/Outsider (FISH).tif]

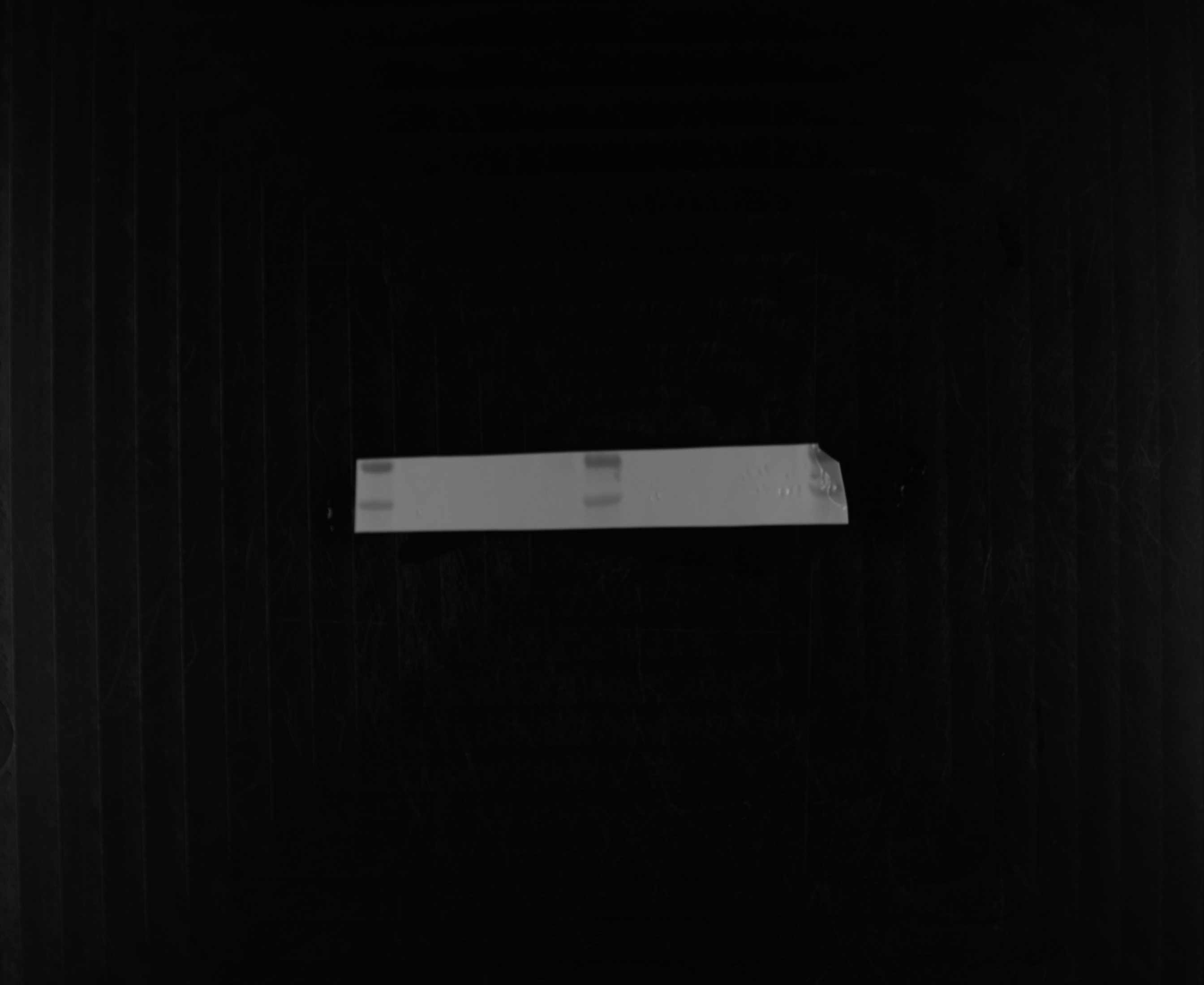

Supplement: Supplementary file 11 — Figure EV3 Source Data [file 44318_2025_502_MOESM11_ESM.zip › Figure EV3/Fig EV3A/TRF1 - marker.Tif]

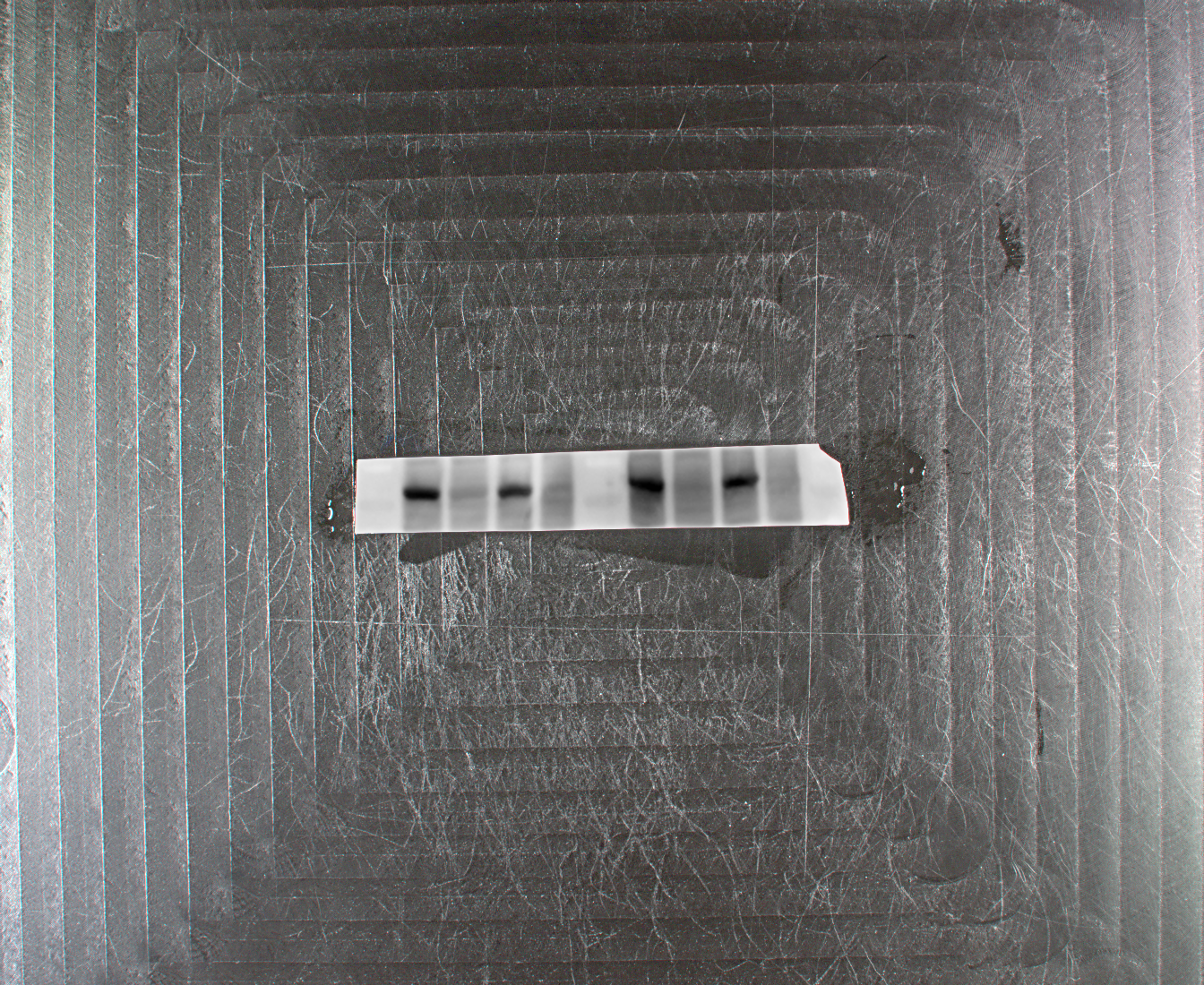

Supplement: Supplementary file 11 — Figure EV3 Source Data [file 44318_2025_502_MOESM11_ESM.zip › Figure EV3/Fig EV3A/TRF1.Tif]

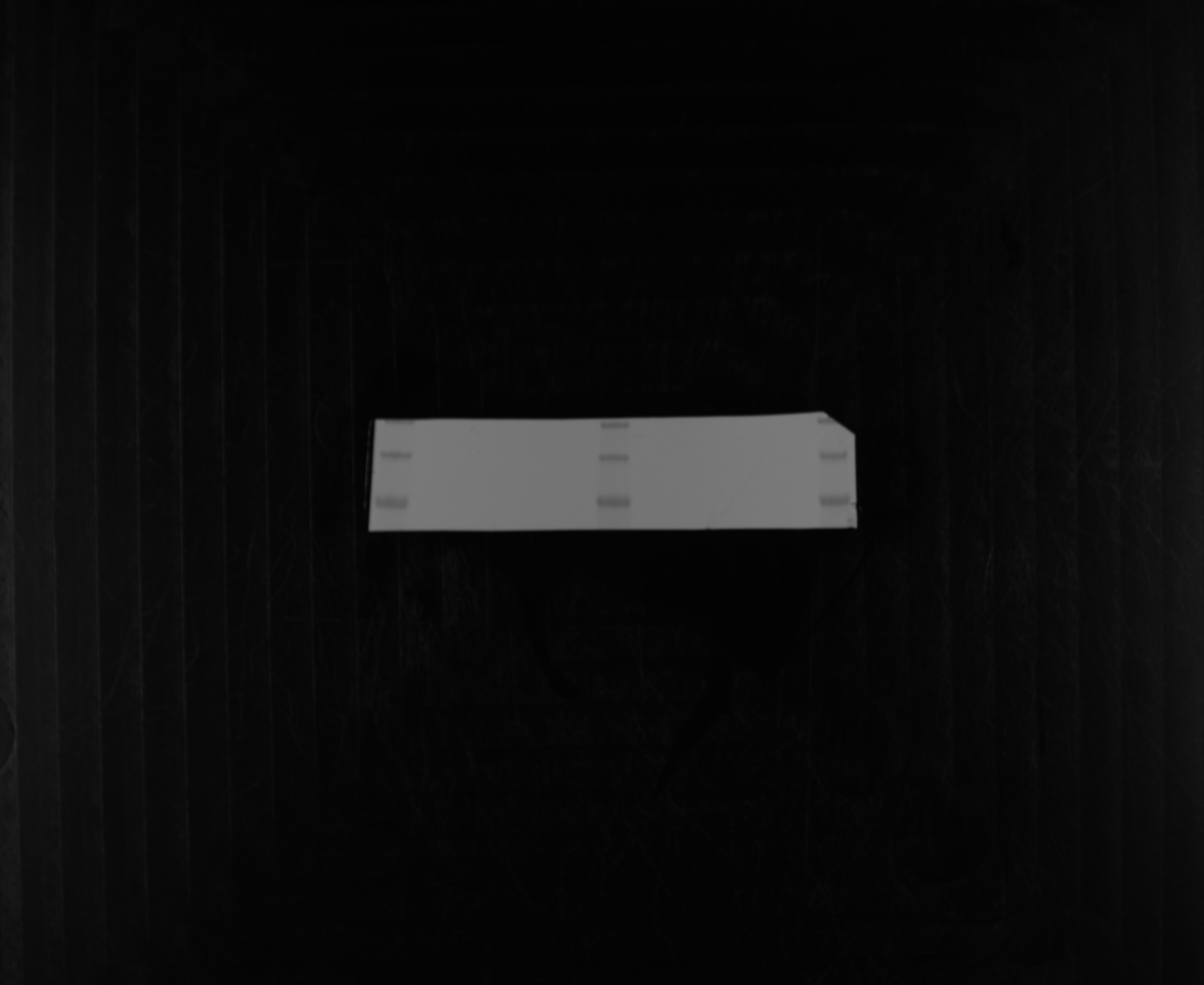

Supplement: Supplementary file 11 — Figure EV3 Source Data [file 44318_2025_502_MOESM11_ESM.zip › Figure EV3/Fig EV3A/Vinculin - marker.Tif]

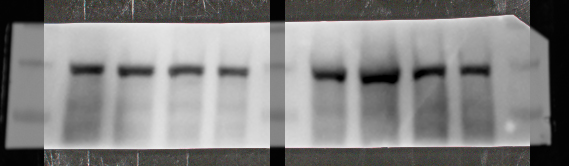

Supplement: Supplementary file 11 — Figure EV3 Source Data [file 44318_2025_502_MOESM11_ESM.zip › Figure EV3/Fig EV3A/Vinculin - merge.jpg]

Fig EV3A

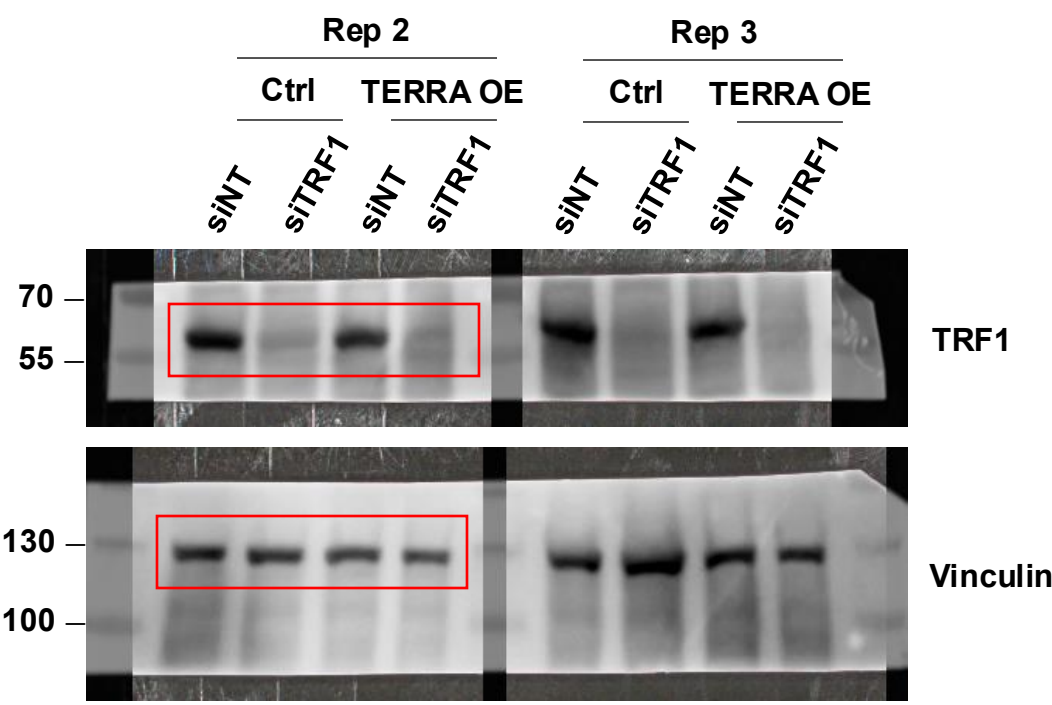

Supplement: Supplementary file 11 — Figure EV3 Source Data [file 44318_2025_502_MOESM11_ESM.zip › Figure EV3/Fig EV3A/Fig EV3A.pdf]

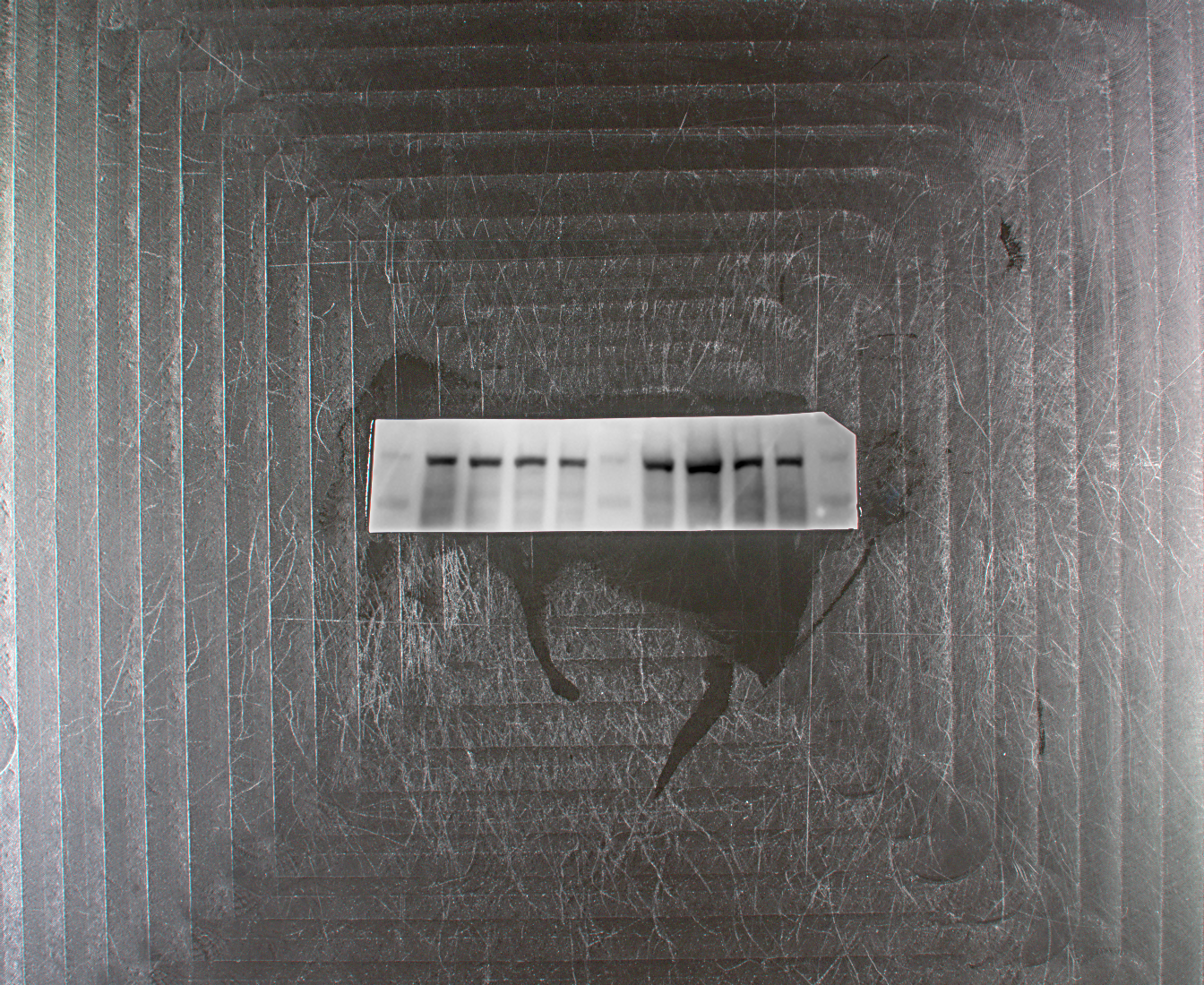

Supplement: Supplementary file 11 — Figure EV3 Source Data [file 44318_2025_502_MOESM11_ESM.zip › Figure EV3/Fig EV3A/Vinculin.Tif]

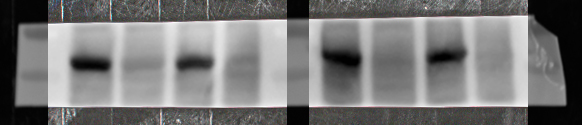

Supplement: Supplementary file 11 — Figure EV3 Source Data [file 44318_2025_502_MOESM11_ESM.zip › Figure EV3/Fig EV3A/TRF1 - merge.jpg]

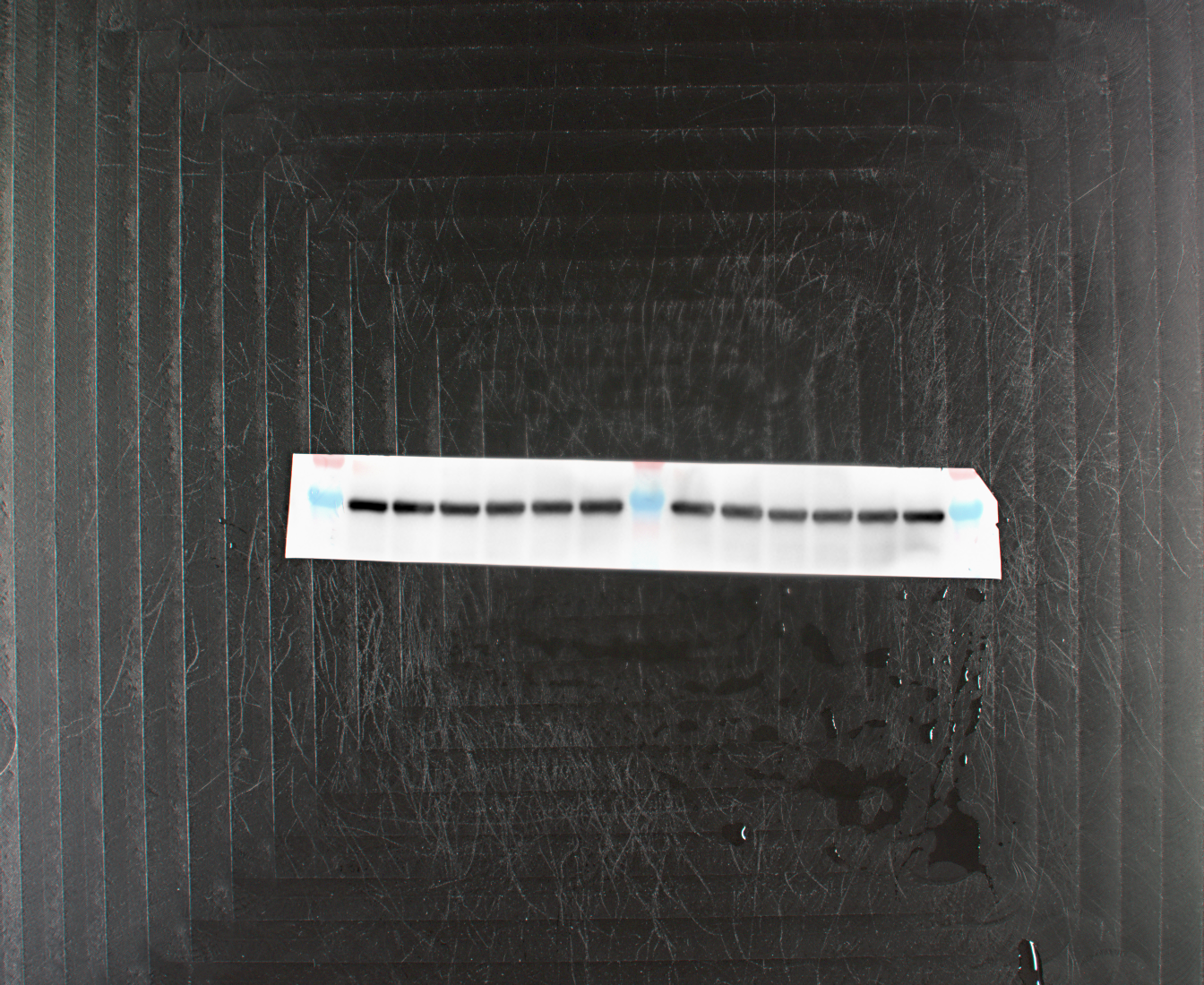

Supplement: Supplementary file 11 — Figure EV3 Source Data [file 44318_2025_502_MOESM11_ESM.zip › Figure EV3/Fig EV3B/Tubulin.Tif]

Fig EV3B

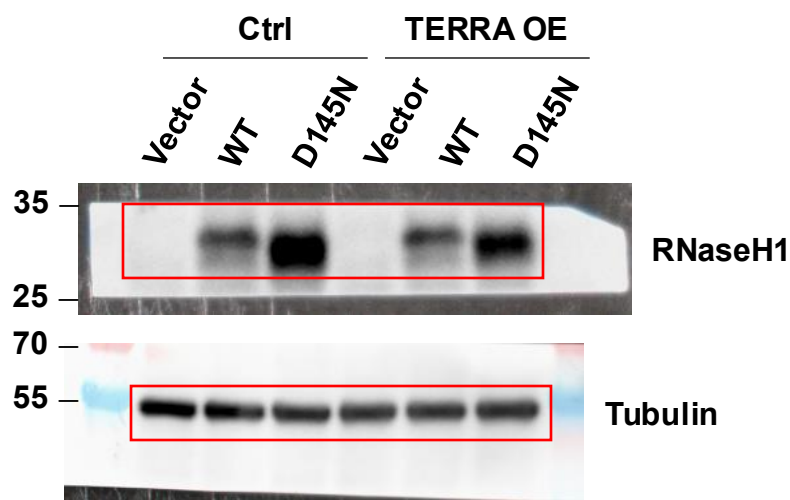

Supplement: Supplementary file 11 — Figure EV3 Source Data [file 44318_2025_502_MOESM11_ESM.zip › Figure EV3/Fig EV3B/Fig EV3B.pdf]

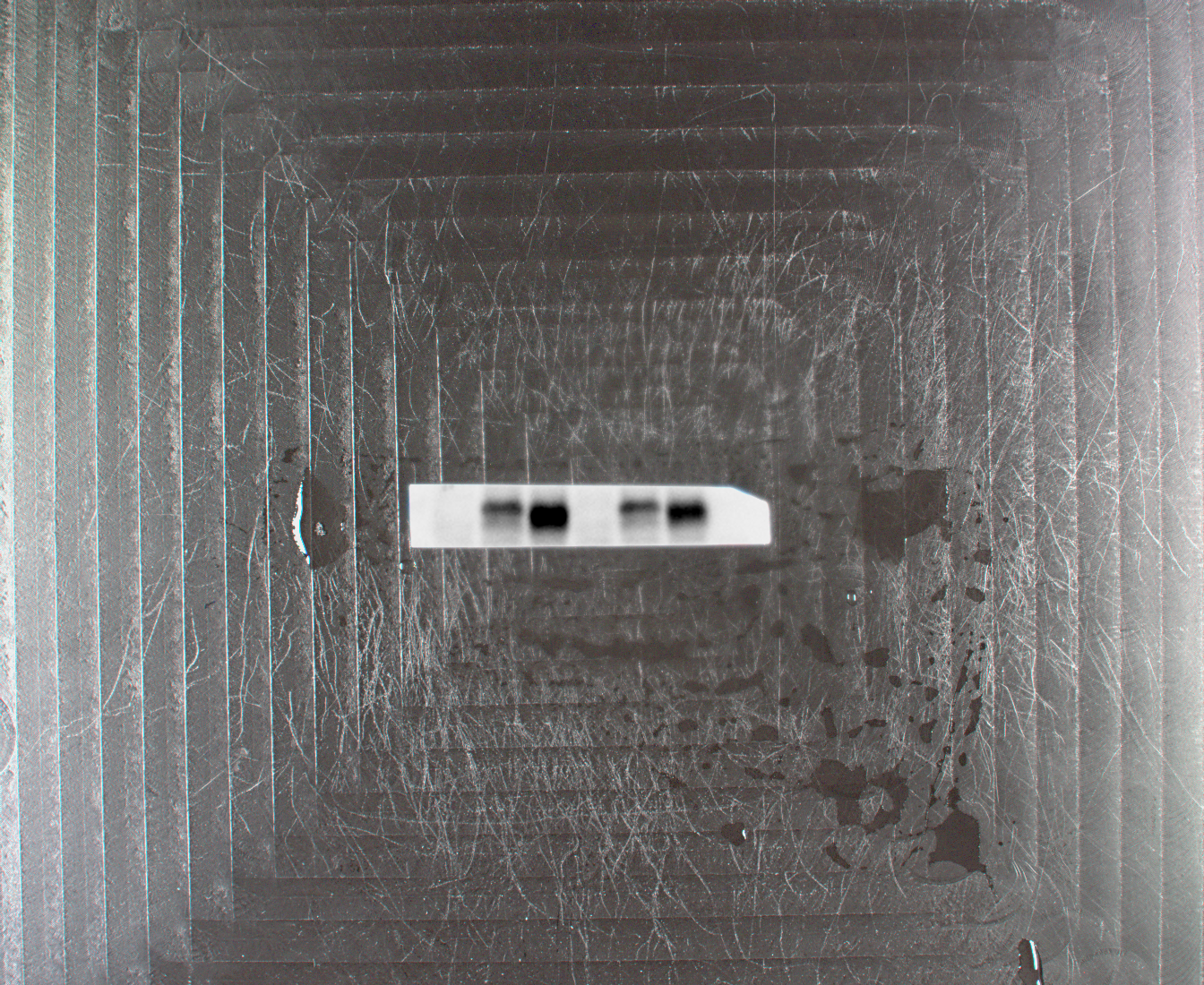

Supplement: Supplementary file 11 — Figure EV3 Source Data [file 44318_2025_502_MOESM11_ESM.zip › Figure EV3/Fig EV3B/RNaseH1.Tif]

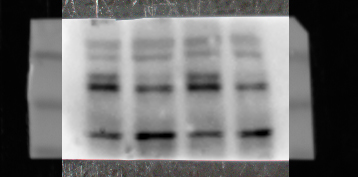

Supplement: Supplementary file 11 — Figure EV3 Source Data [file 44318_2025_502_MOESM11_ESM.zip › Figure EV3/Fig EV3C/RNaseH1 - merge.jpg]

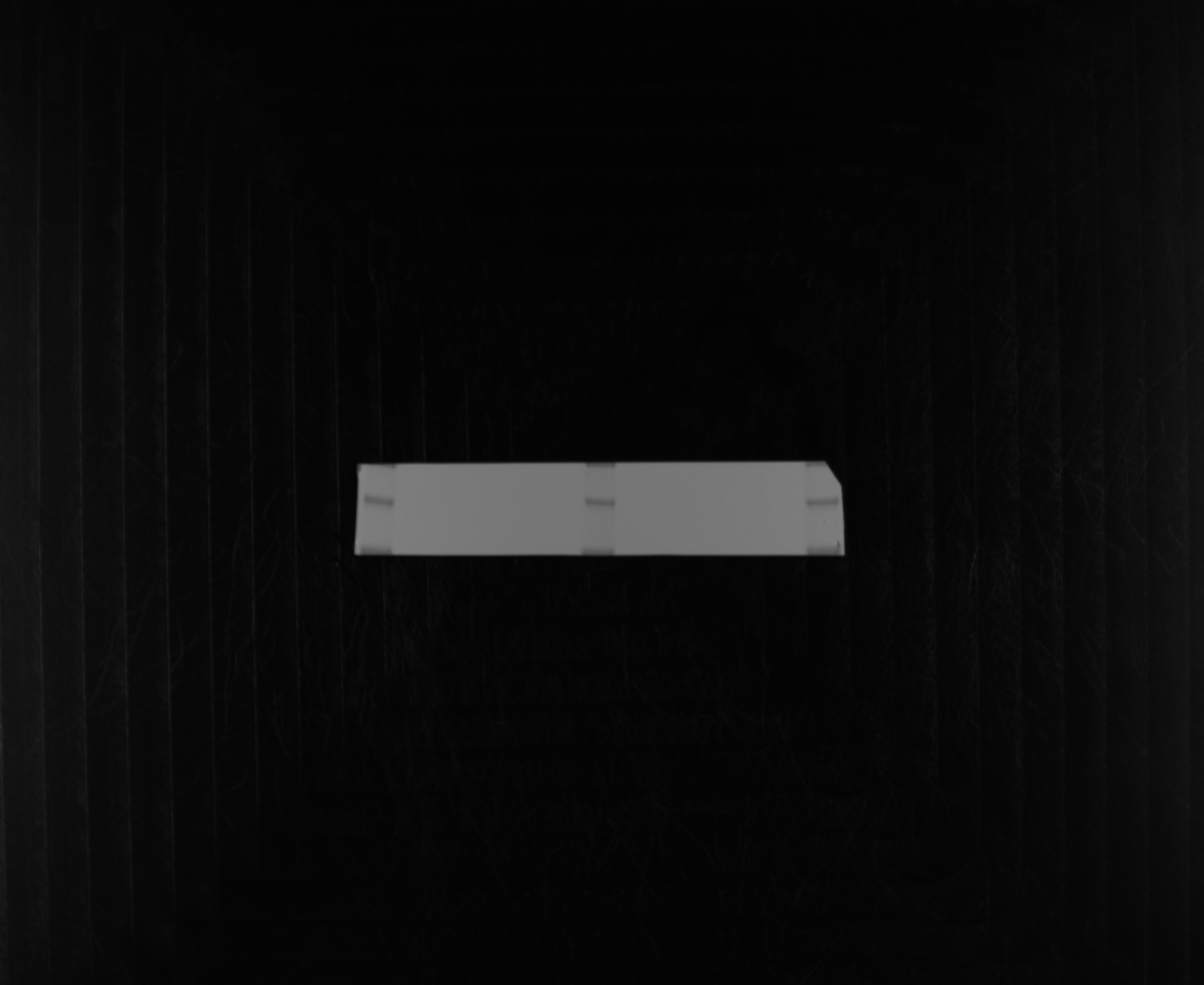

Supplement: Supplementary file 11 — Figure EV3 Source Data [file 44318_2025_502_MOESM11_ESM.zip › Figure EV3/Fig EV3C/Vinculin - marker.Tif]

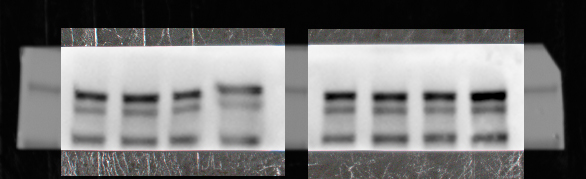

Supplement: Supplementary file 11 — Figure EV3 Source Data [file 44318_2025_502_MOESM11_ESM.zip › Figure EV3/Fig EV3C/Vinculin - merge.jpg]

Fig EV3C

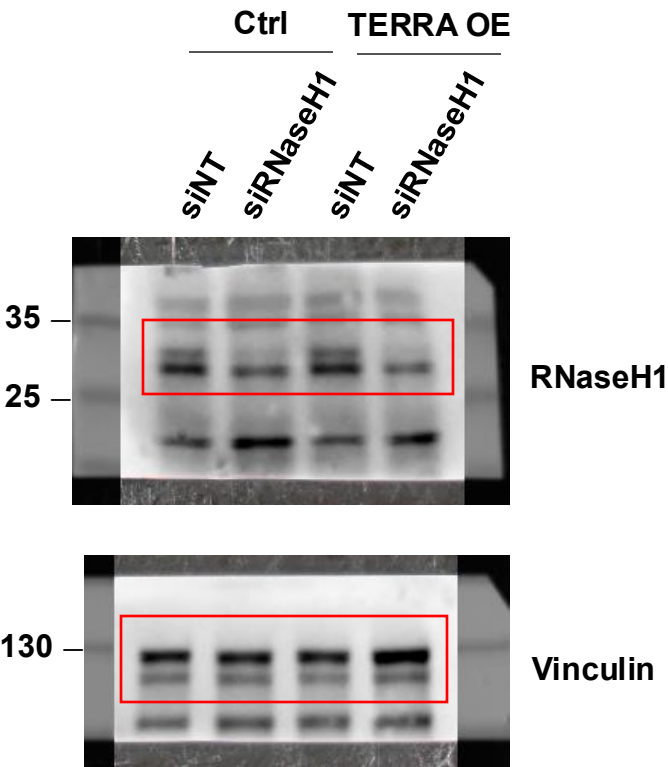

Supplement: Supplementary file 11 — Figure EV3 Source Data [file 44318_2025_502_MOESM11_ESM.zip › Figure EV3/Fig EV3C/Fig EV3C.pdf]

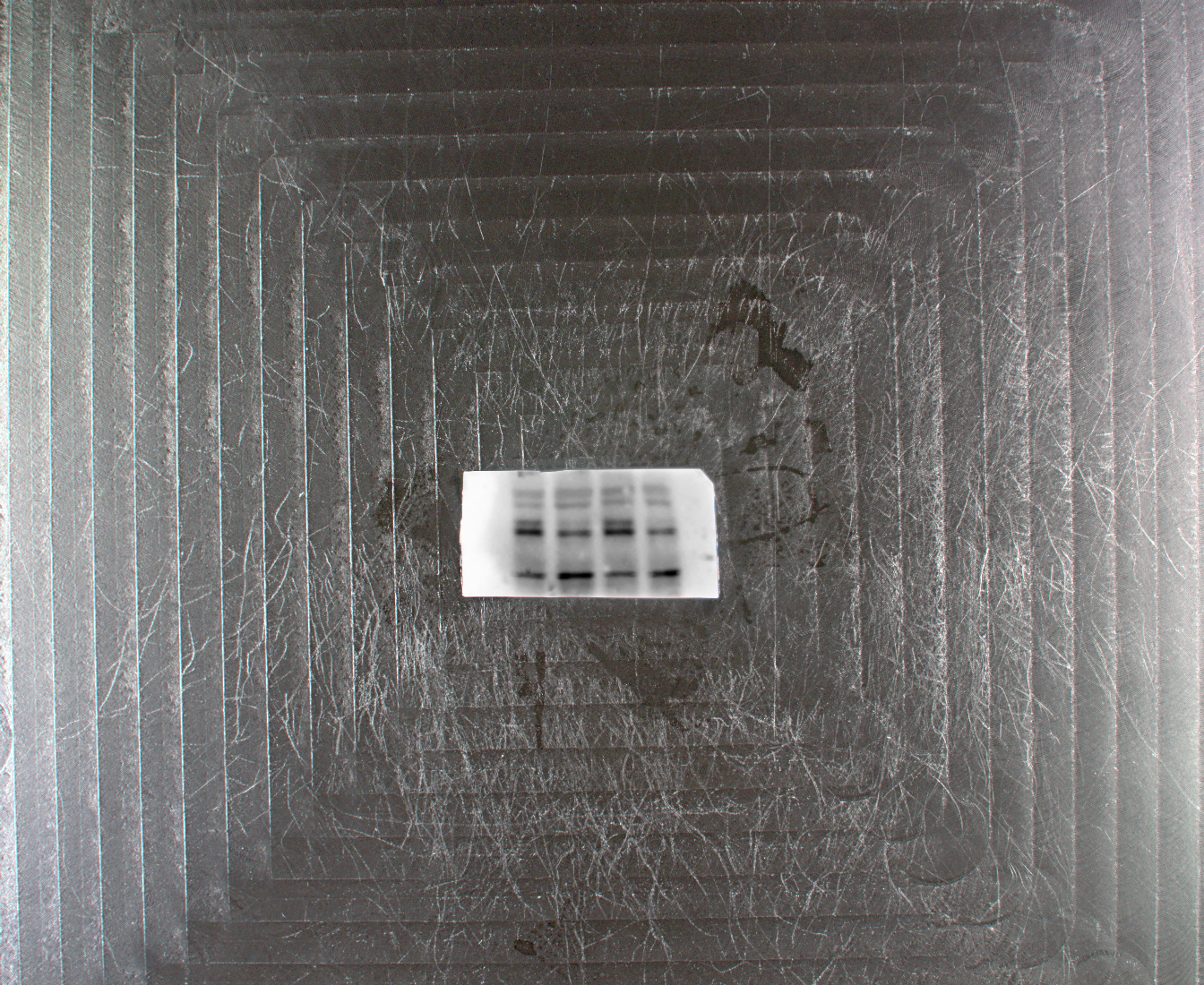

Supplement: Supplementary file 11 — Figure EV3 Source Data [file 44318_2025_502_MOESM11_ESM.zip › Figure EV3/Fig EV3C/RNaseH1.Tif]

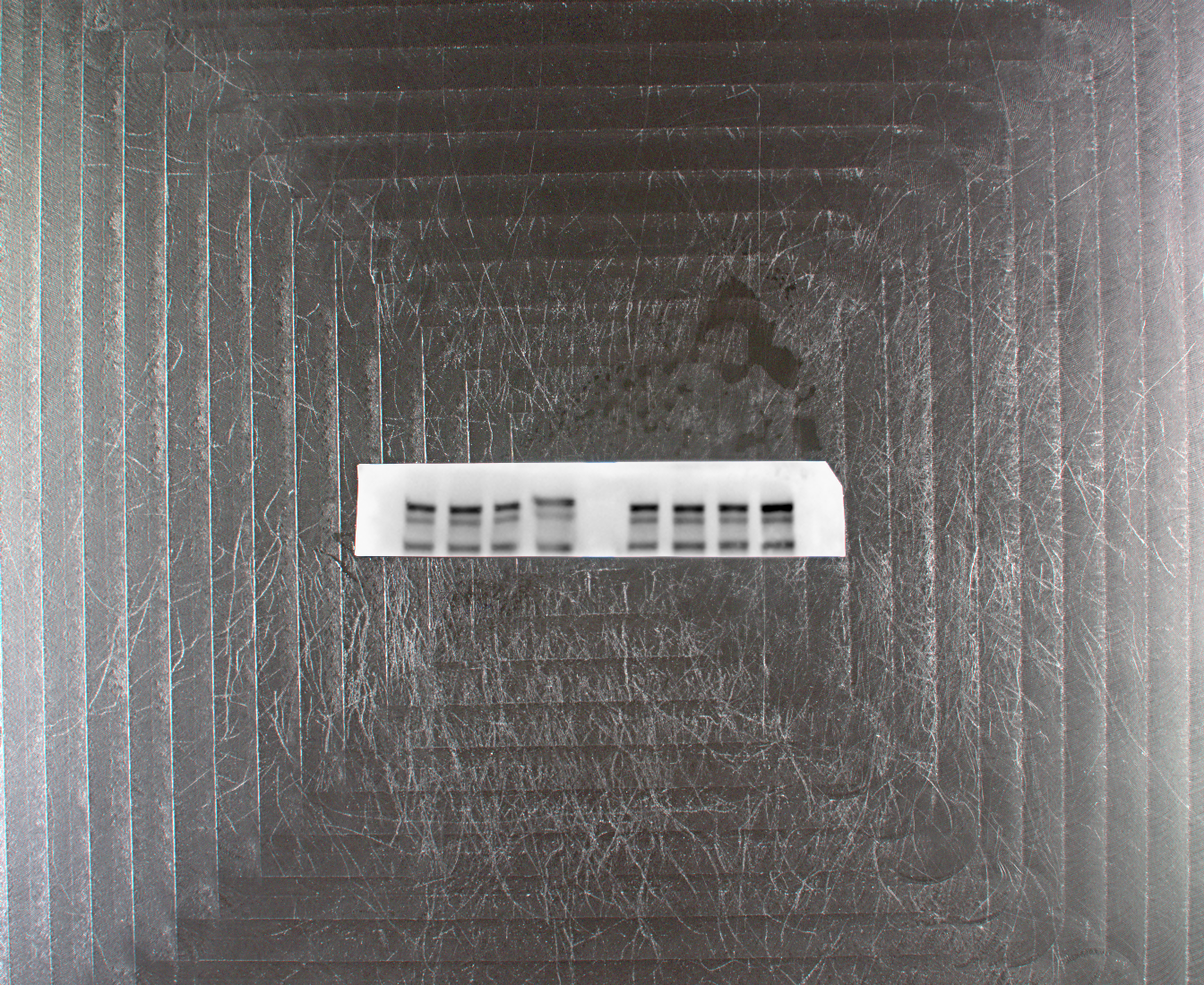

Supplement: Supplementary file 11 — Figure EV3 Source Data [file 44318_2025_502_MOESM11_ESM.zip › Figure EV3/Fig EV3C/Vinculin.Tif]

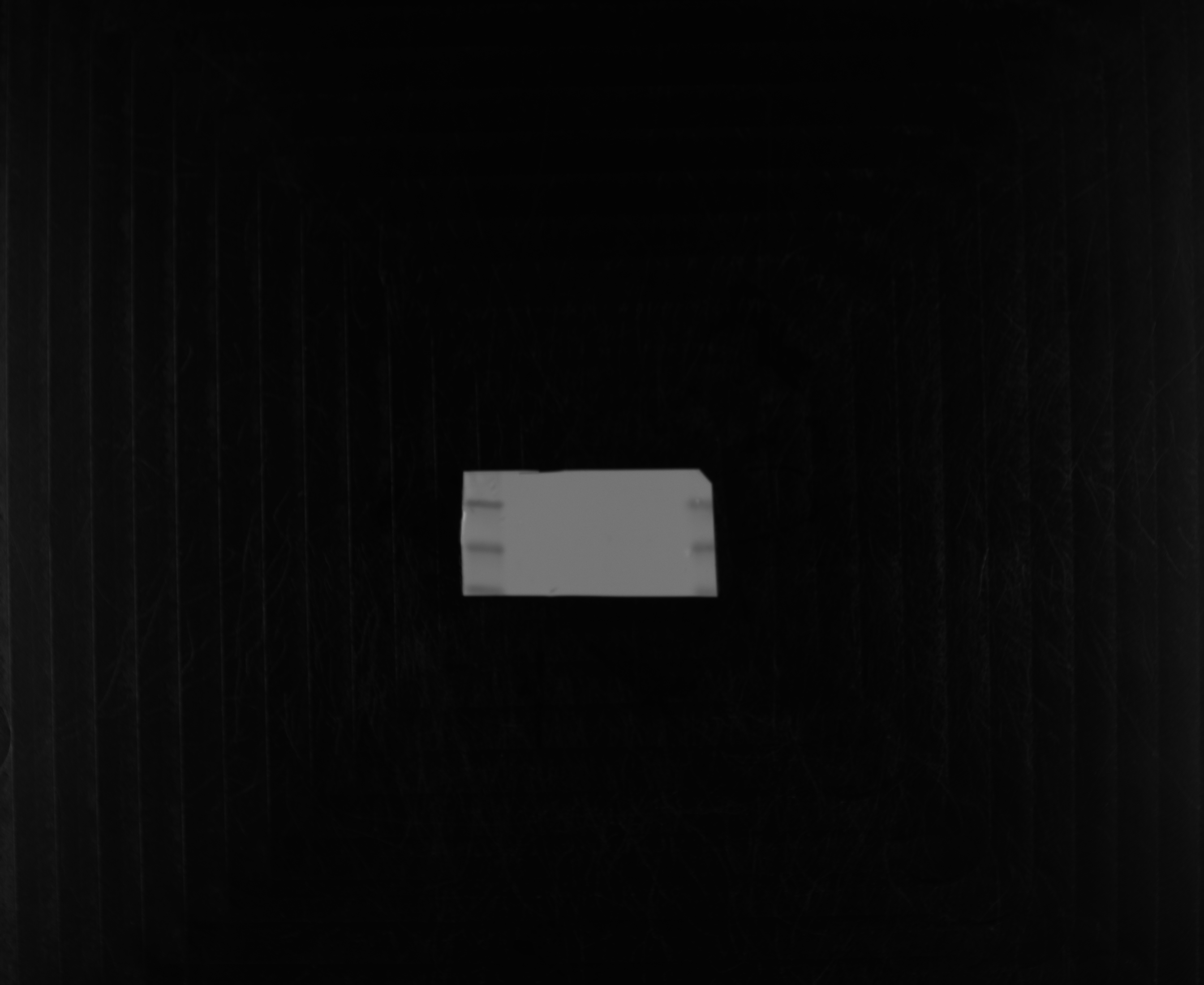

Supplement: Supplementary file 11 — Figure EV3 Source Data [file 44318_2025_502_MOESM11_ESM.zip › Figure EV3/Fig EV3C/RNaseH1 - marker.Tif]

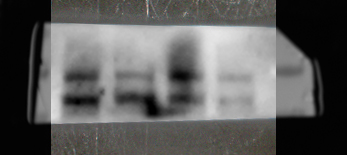

Supplement: Supplementary file 12 — Figure EV4 Source Data [file 44318_2025_502_MOESM12_ESM.zip › Figure EV4/Fig EV4A/PrimPol - merge.jpg]

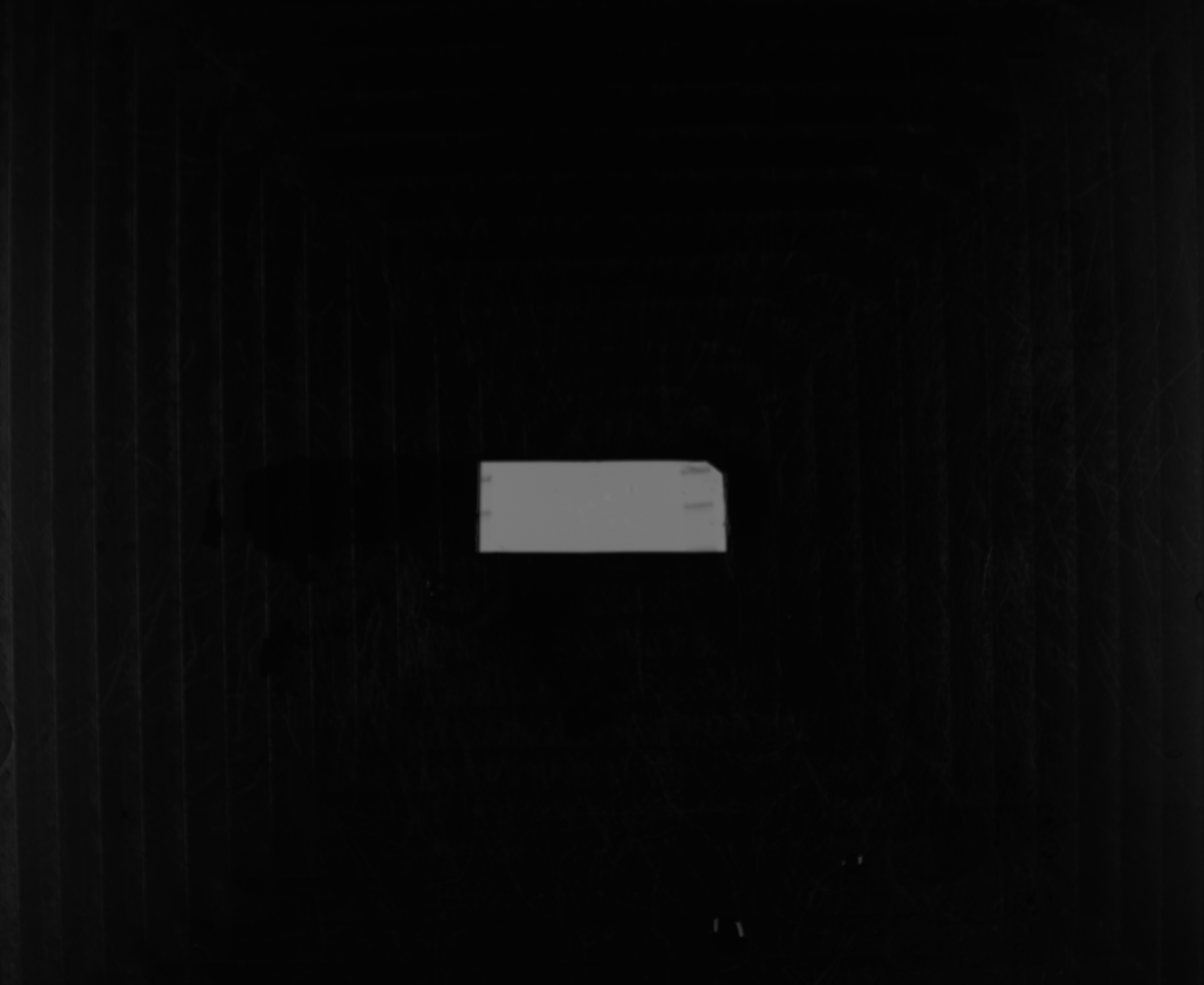

Supplement: Supplementary file 12 — Figure EV4 Source Data [file 44318_2025_502_MOESM12_ESM.zip › Figure EV4/Fig EV4A/Vinculin - marker.Tif]

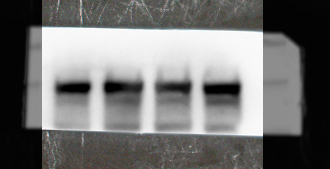

Supplement: Supplementary file 12 — Figure EV4 Source Data [file 44318_2025_502_MOESM12_ESM.zip › Figure EV4/Fig EV4A/Vinculin - merge.jpg]

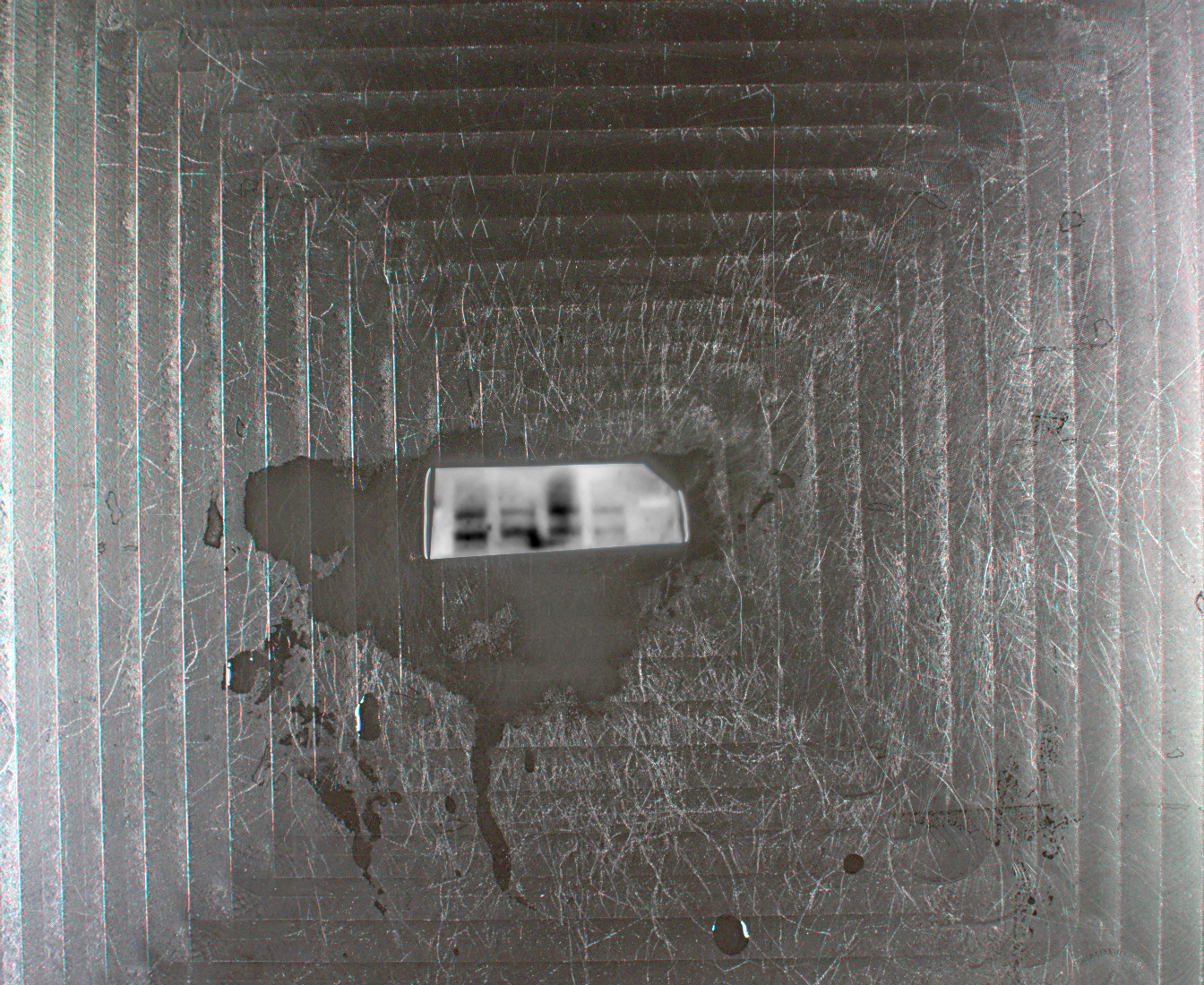

Supplement: Supplementary file 12 — Figure EV4 Source Data [file 44318_2025_502_MOESM12_ESM.zip › Figure EV4/Fig EV4A/PrimPol.Tif]

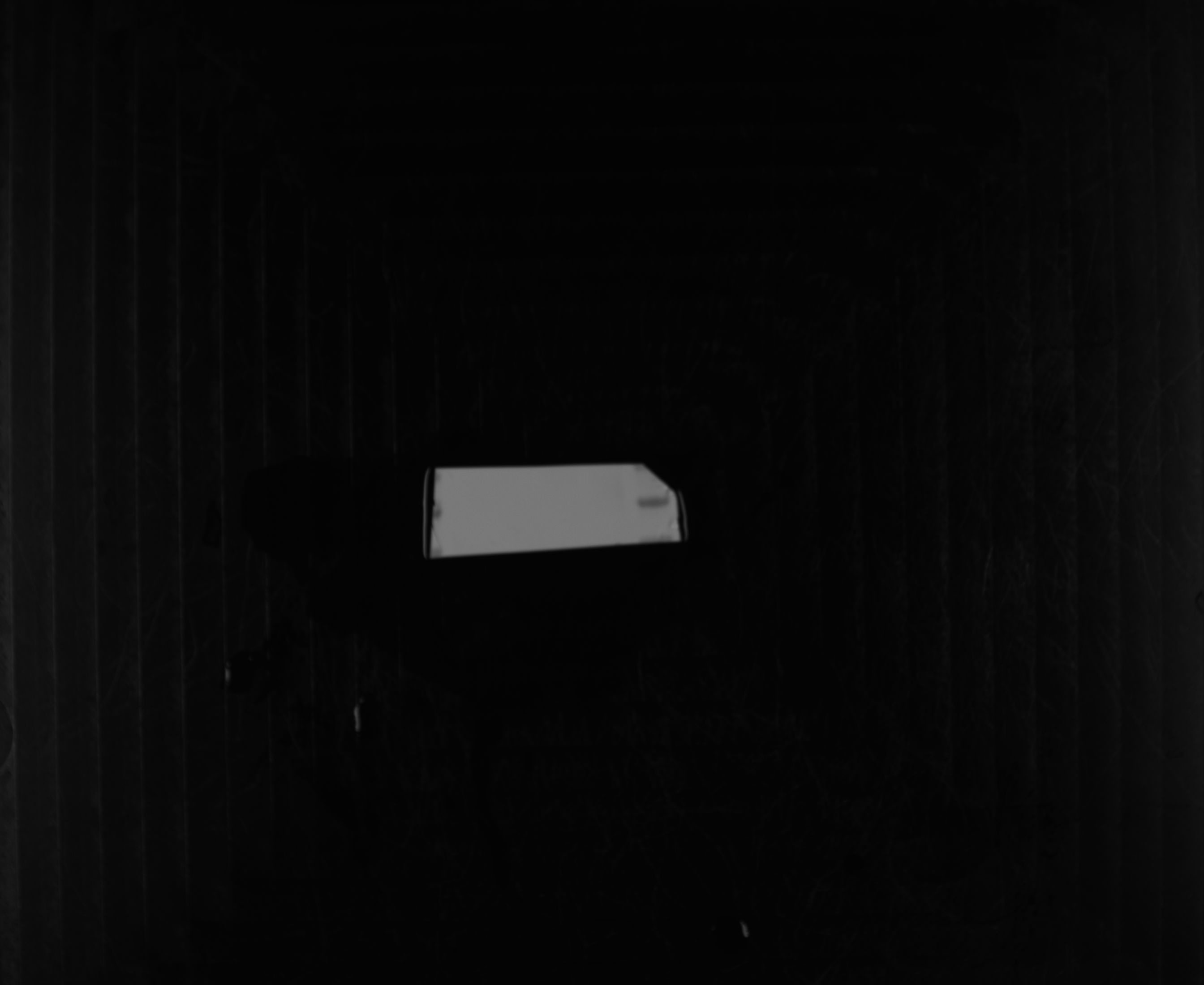

Supplement: Supplementary file 12 — Figure EV4 Source Data [file 44318_2025_502_MOESM12_ESM.zip › Figure EV4/Fig EV4A/PrimPol - marker.Tif]

Fig EV4A

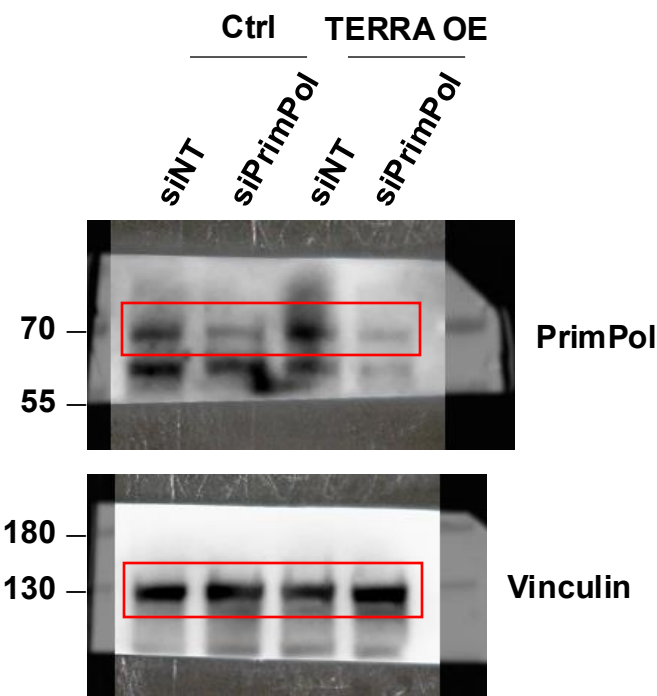

Supplement: Supplementary file 12 — Figure EV4 Source Data [file 44318_2025_502_MOESM12_ESM.zip › Figure EV4/Fig EV4A/Fig EV4A.pdf]

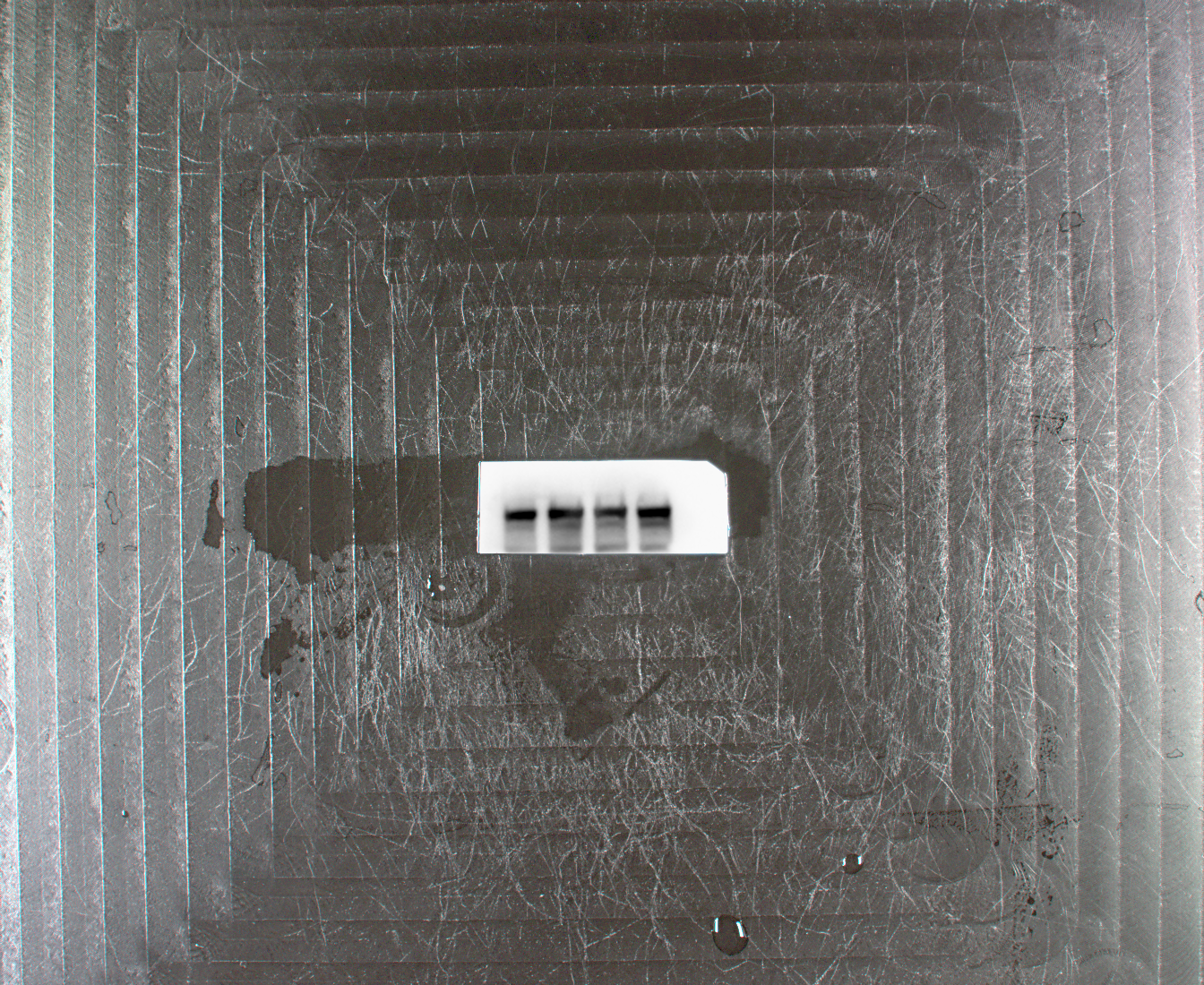

Supplement: Supplementary file 12 — Figure EV4 Source Data [file 44318_2025_502_MOESM12_ESM.zip › Figure EV4/Fig EV4A/Vinculin.Tif]

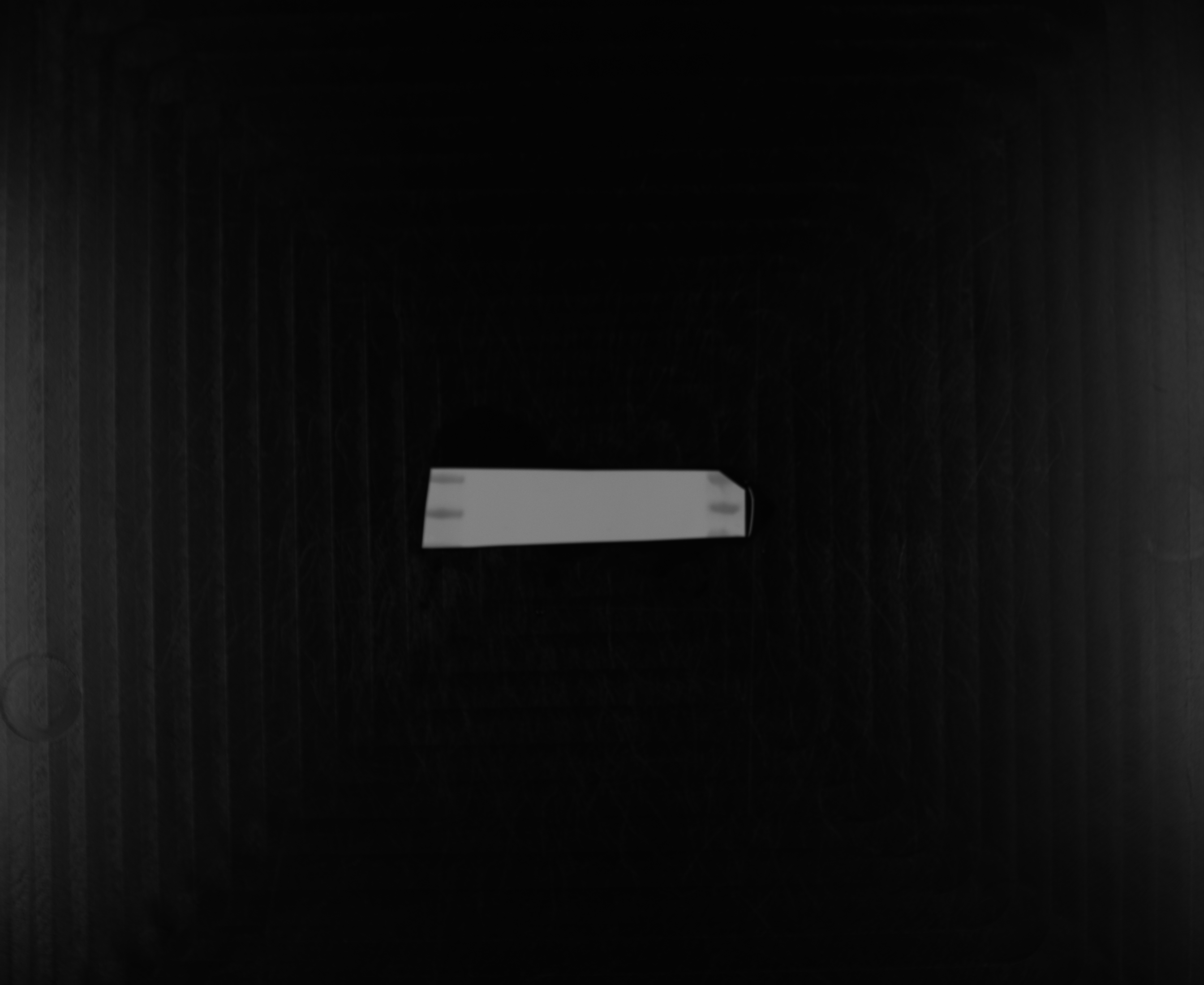

Supplement: Supplementary file 12 — Figure EV4 Source Data [file 44318_2025_502_MOESM12_ESM.zip › Figure EV4/Fig EV4B/POLD3 - marker.Tif]

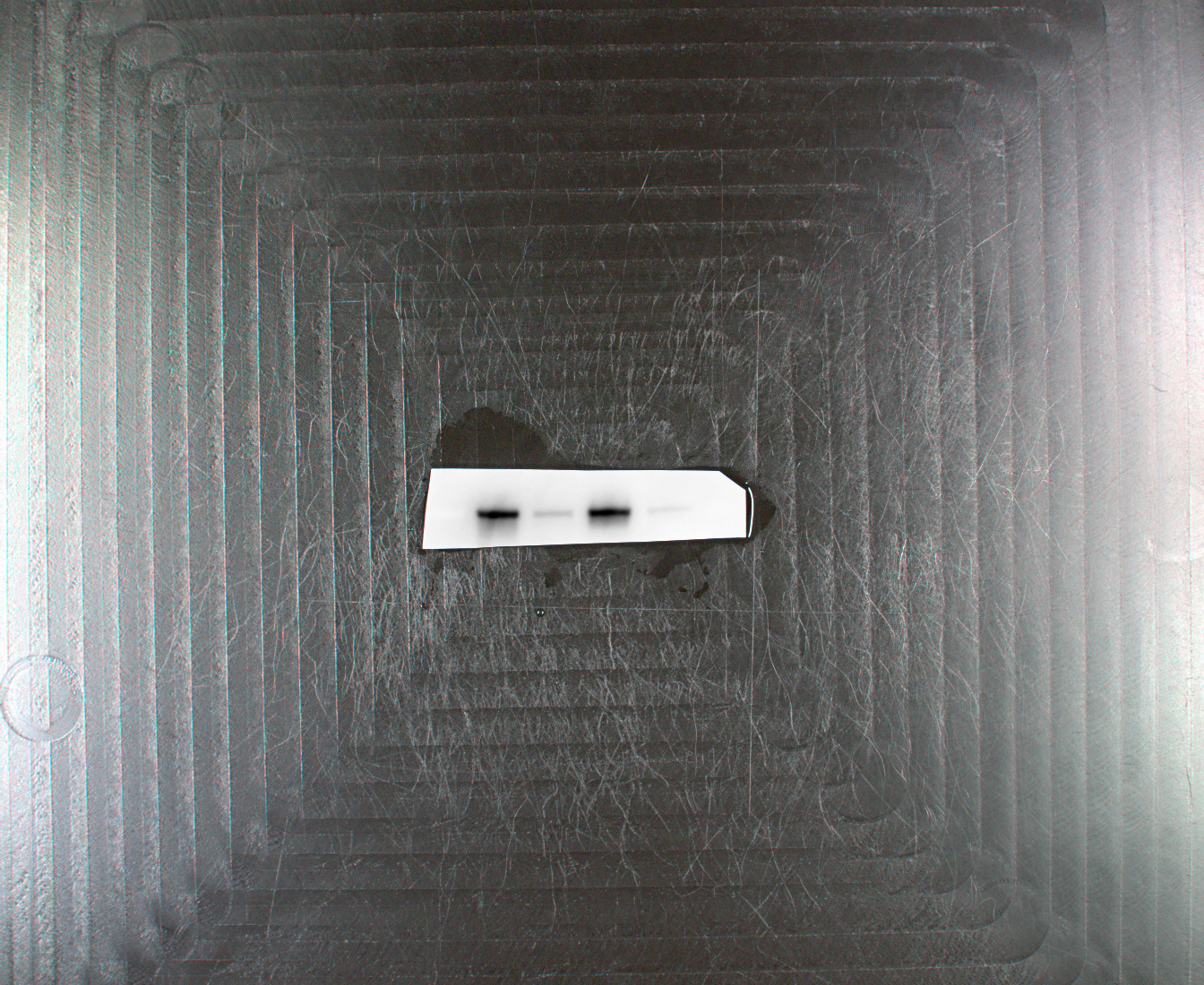

Supplement: Supplementary file 12 — Figure EV4 Source Data [file 44318_2025_502_MOESM12_ESM.zip › Figure EV4/Fig EV4B/POLD3.Tif]

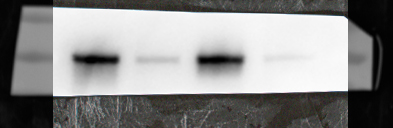

Supplement: Supplementary file 12 — Figure EV4 Source Data [file 44318_2025_502_MOESM12_ESM.zip › Figure EV4/Fig EV4B/POLD3 - merge.jpg]

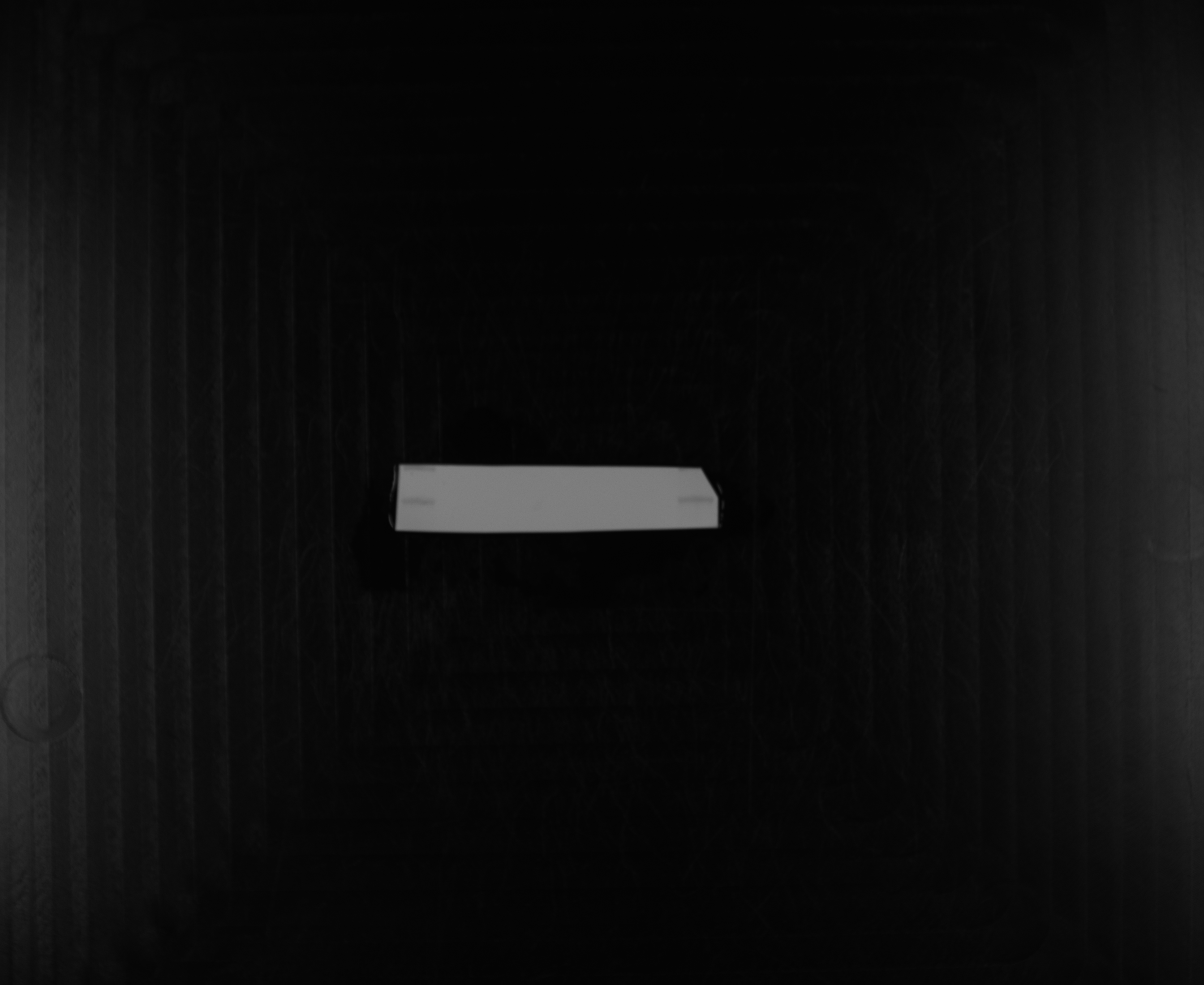

Supplement: Supplementary file 12 — Figure EV4 Source Data [file 44318_2025_502_MOESM12_ESM.zip › Figure EV4/Fig EV4B/Vinculin - marker.Tif]

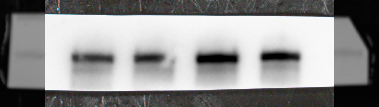

Supplement: Supplementary file 12 — Figure EV4 Source Data [file 44318_2025_502_MOESM12_ESM.zip › Figure EV4/Fig EV4B/Vinculin - merge.jpg]

Fig EV4B

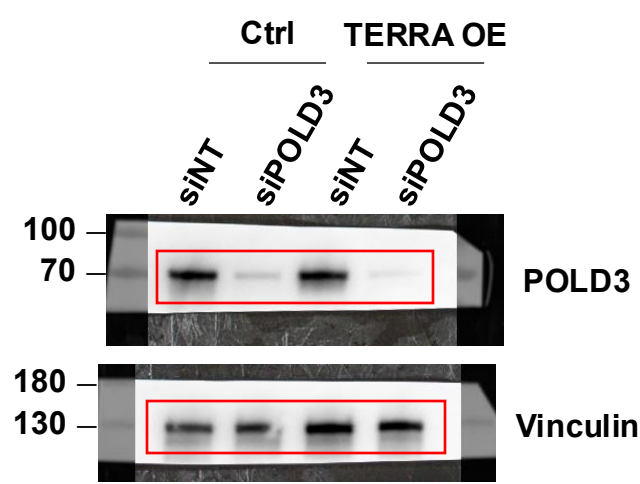

Supplement: Supplementary file 12 — Figure EV4 Source Data [file 44318_2025_502_MOESM12_ESM.zip › Figure EV4/Fig EV4B/Fig EV4B.pdf]

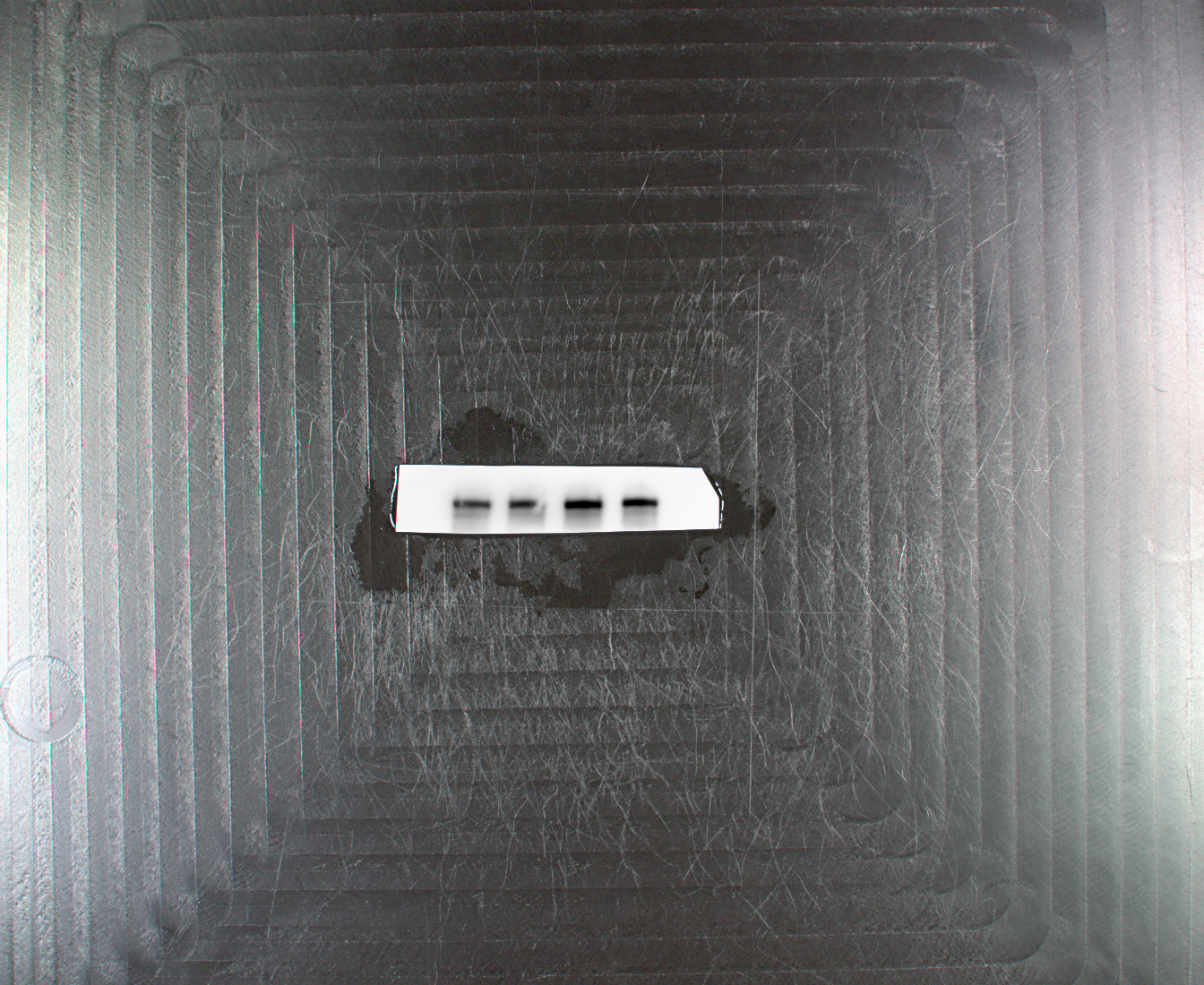

Supplement: Supplementary file 12 — Figure EV4 Source Data [file 44318_2025_502_MOESM12_ESM.zip › Figure EV4/Fig EV4B/Vinculin.Tif]

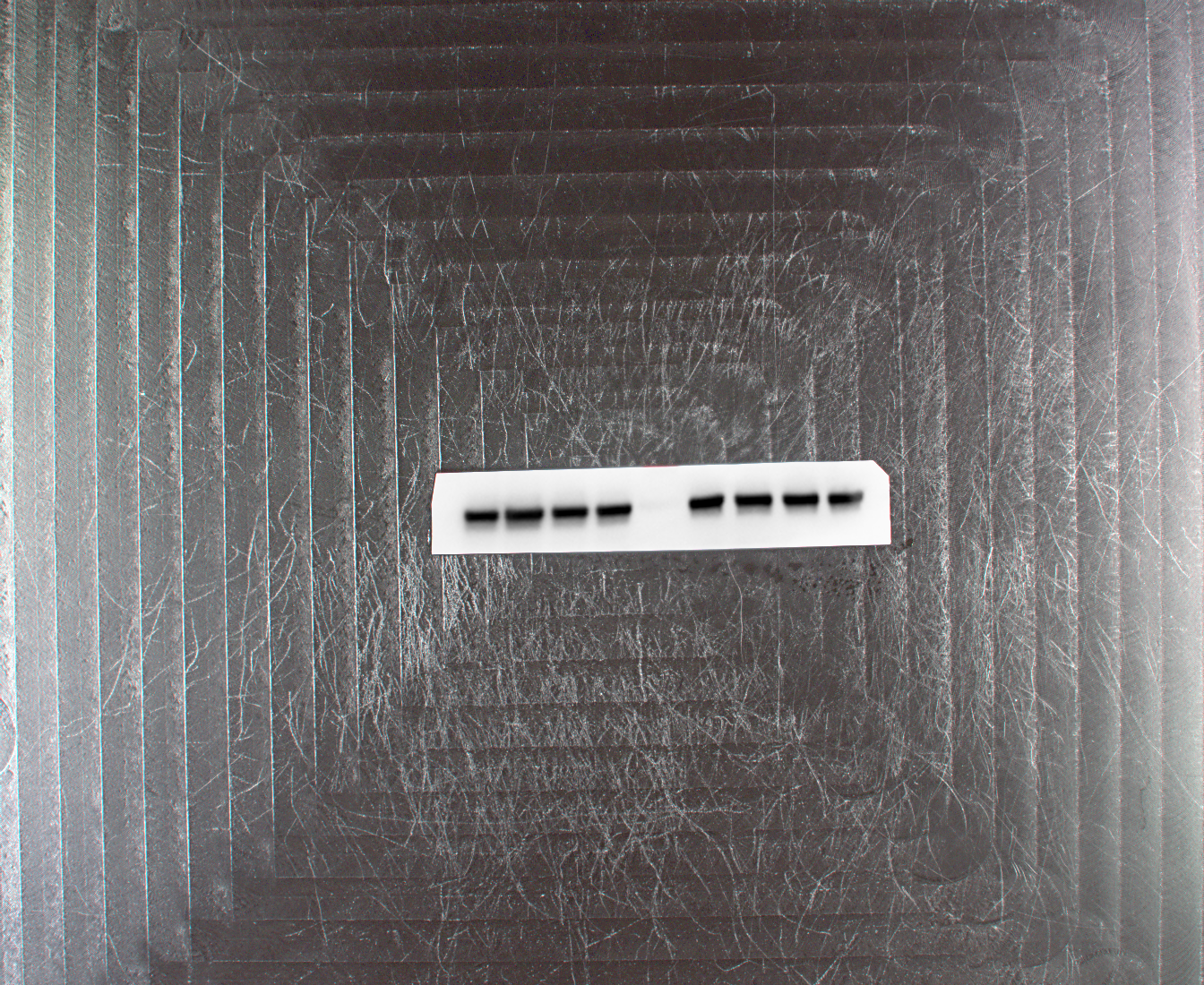

Supplement: Supplementary file 12 — Figure EV4 Source Data [file 44318_2025_502_MOESM12_ESM.zip › Figure EV4/Fig EV4E/Tubulin.Tif]

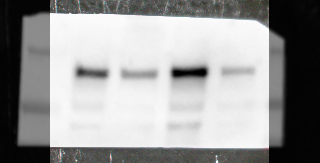

Supplement: Supplementary file 12 — Figure EV4 Source Data [file 44318_2025_502_MOESM12_ESM.zip › Figure EV4/Fig EV4E/SMARCAL1 - merge.jpg]

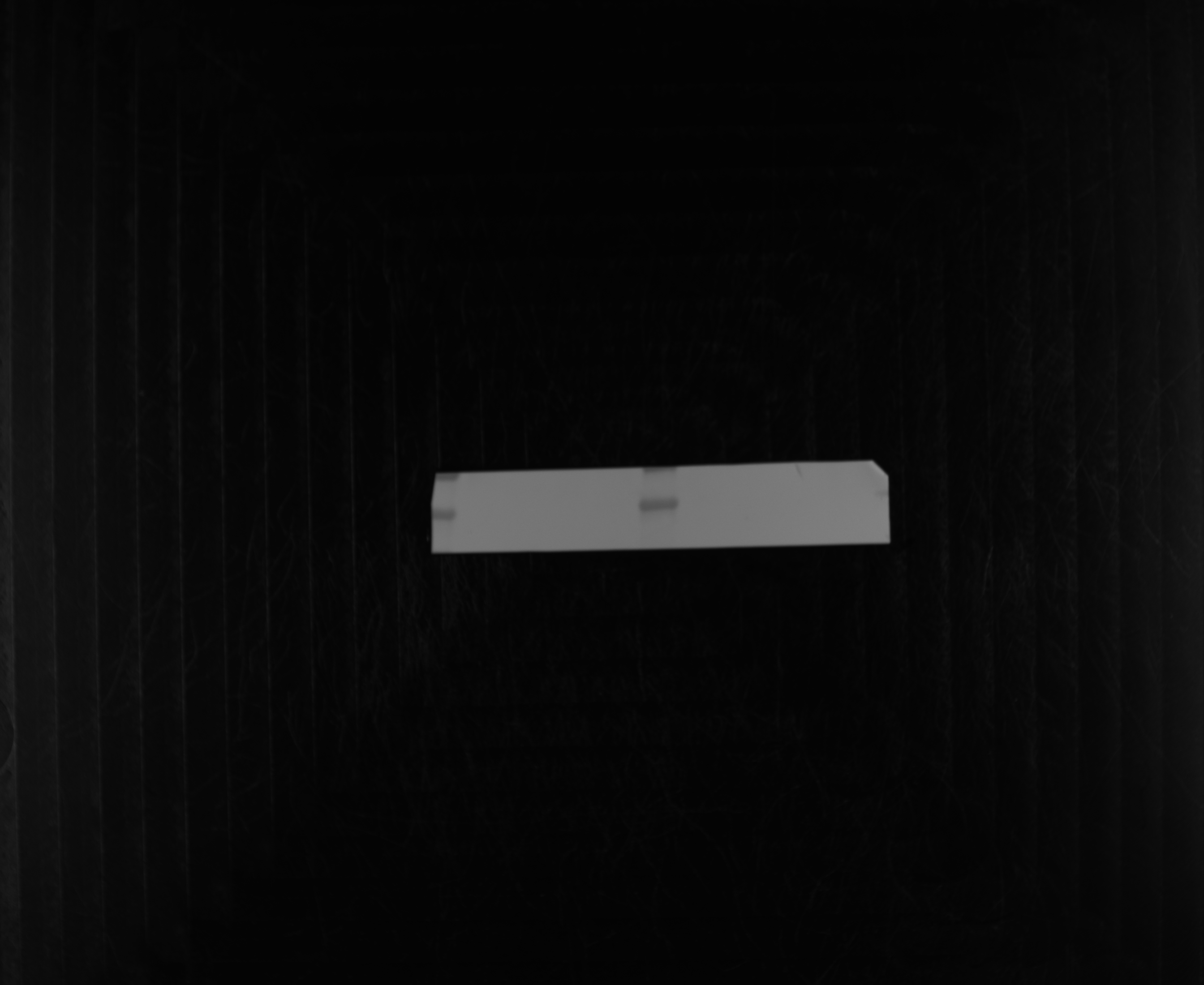

Supplement: Supplementary file 12 — Figure EV4 Source Data [file 44318_2025_502_MOESM12_ESM.zip › Figure EV4/Fig EV4E/Tubulin - marker.Tif]

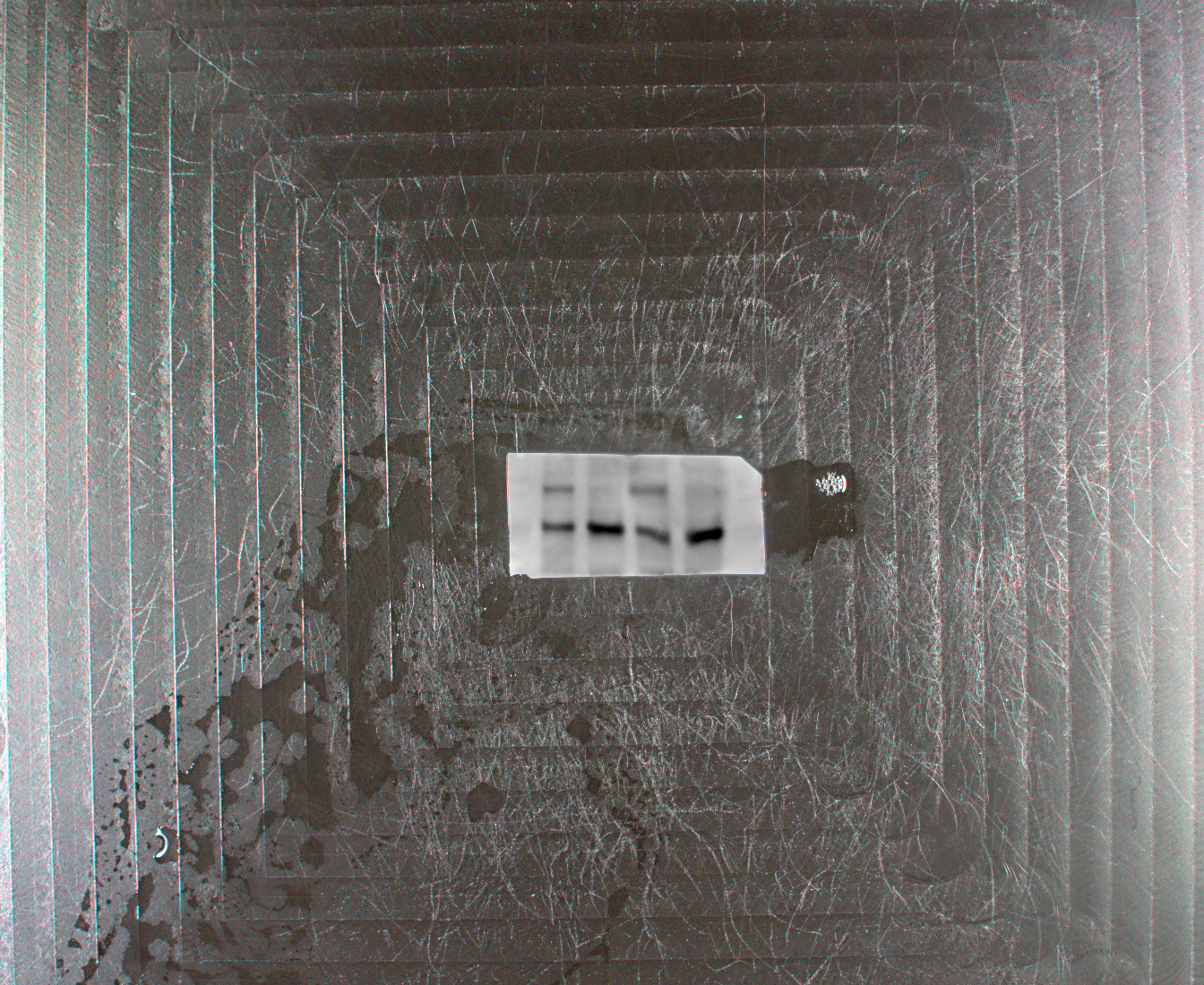

Supplement: Supplementary file 12 — Figure EV4 Source Data [file 44318_2025_502_MOESM12_ESM.zip › Figure EV4/Fig EV4E/MUS81.Tif]

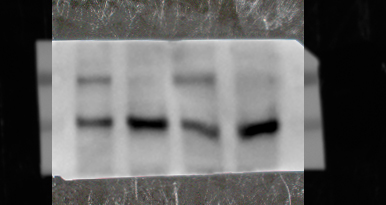

Supplement: Supplementary file 12 — Figure EV4 Source Data [file 44318_2025_502_MOESM12_ESM.zip › Figure EV4/Fig EV4E/MUS81 - merge.jpg]

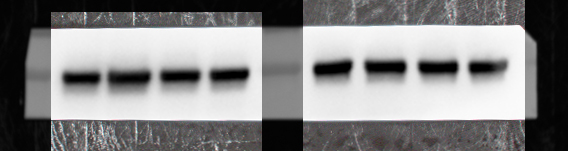

Supplement: Supplementary file 12 — Figure EV4 Source Data [file 44318_2025_502_MOESM12_ESM.zip › Figure EV4/Fig EV4E/Tubulin - merge.jpg]

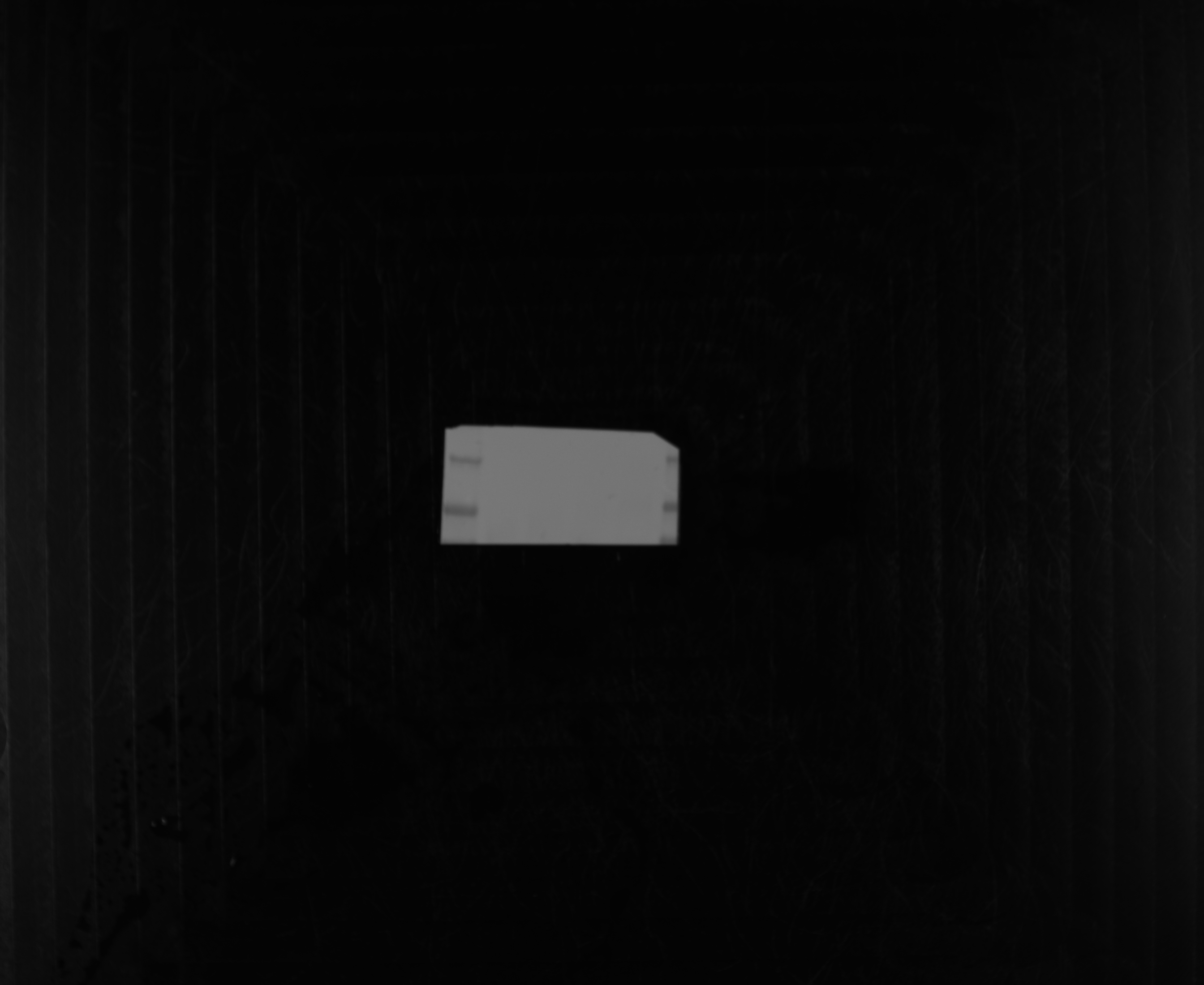

Supplement: Supplementary file 12 — Figure EV4 Source Data [file 44318_2025_502_MOESM12_ESM.zip › Figure EV4/Fig EV4E/SMARCAL1 - marker.Tif]

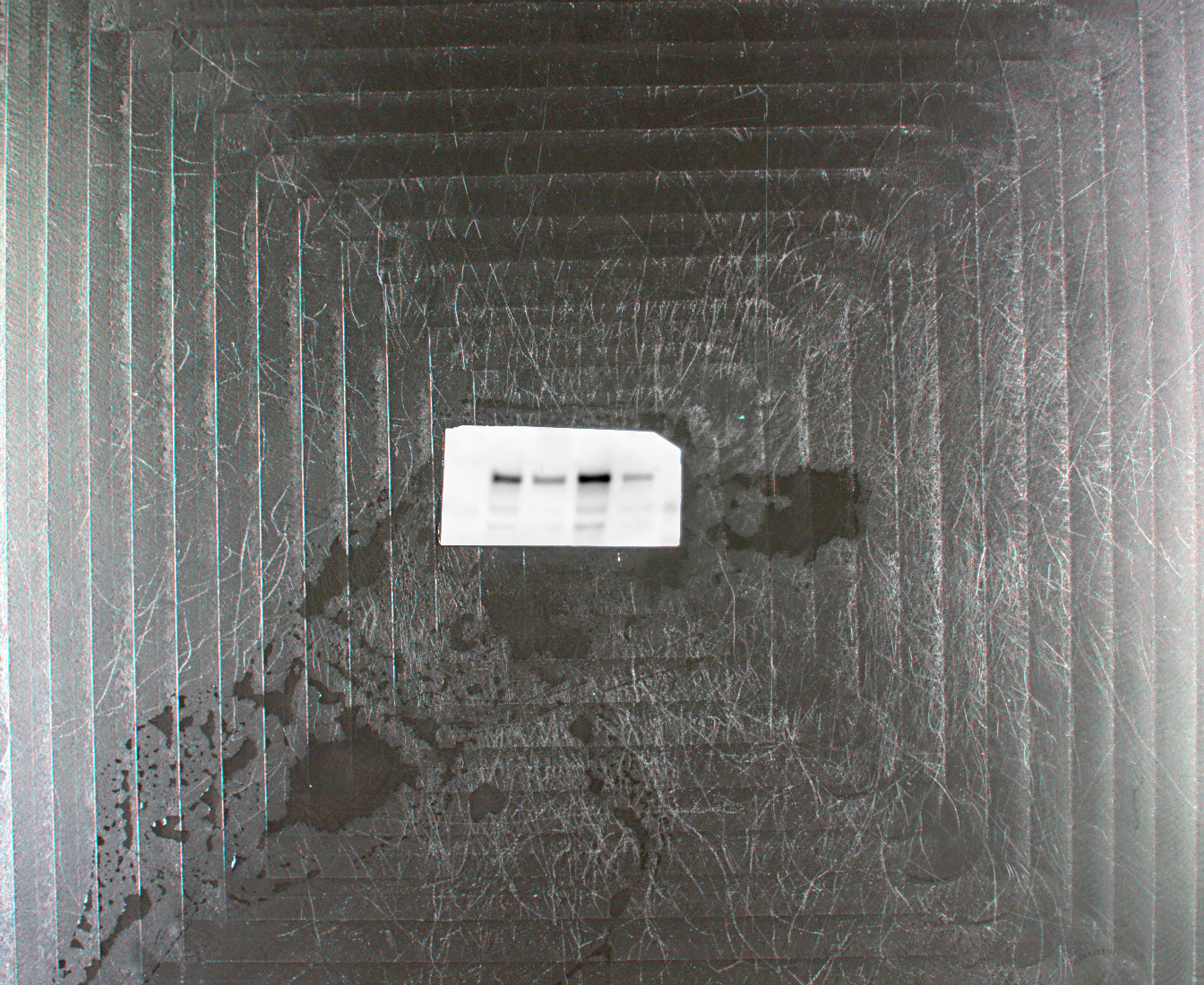

Supplement: Supplementary file 12 — Figure EV4 Source Data [file 44318_2025_502_MOESM12_ESM.zip › Figure EV4/Fig EV4E/SMARCAL1.Tif]

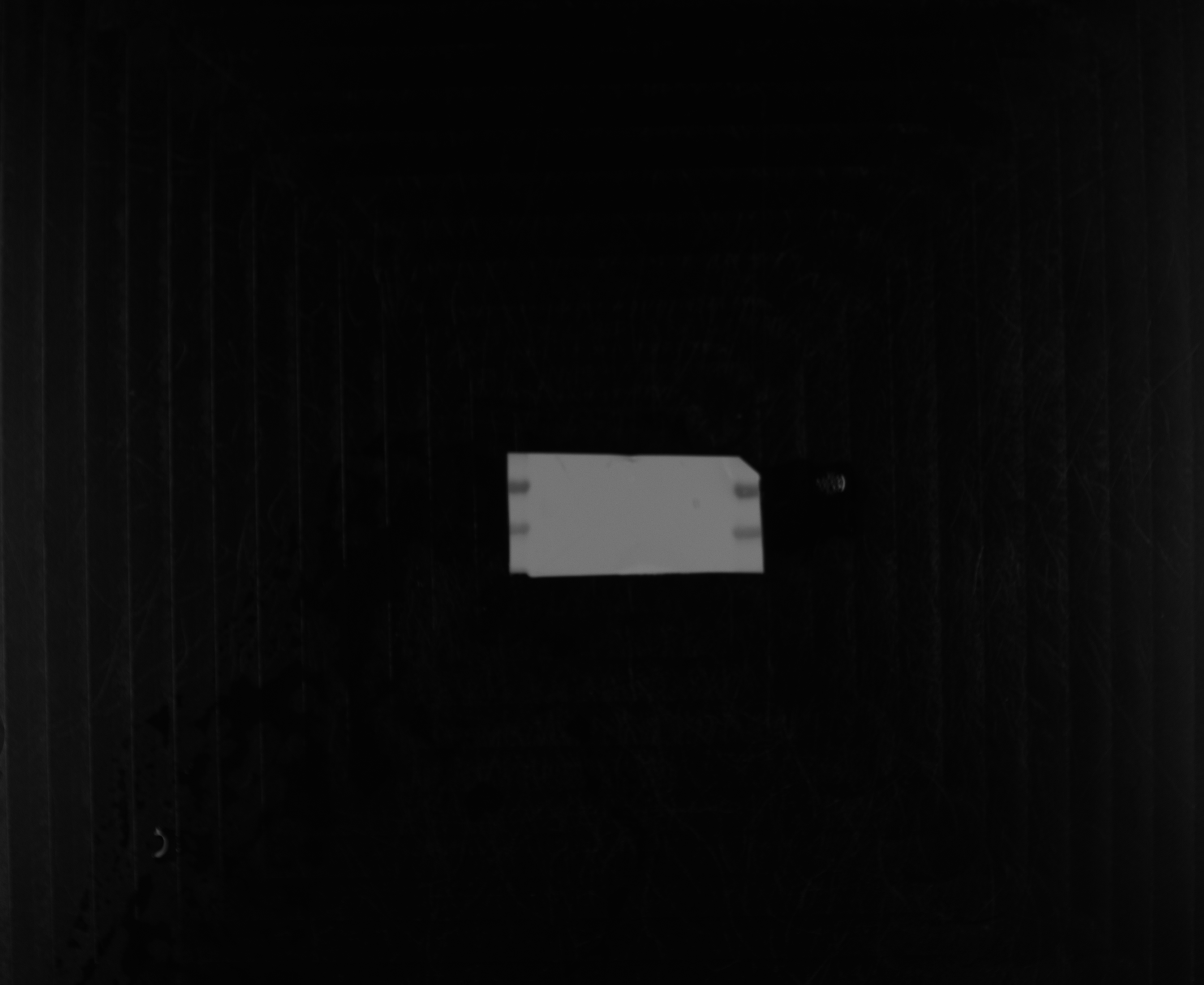

Supplement: Supplementary file 12 — Figure EV4 Source Data [file 44318_2025_502_MOESM12_ESM.zip › Figure EV4/Fig EV4E/MUS81 - marker.Tif]

Fig EV4E

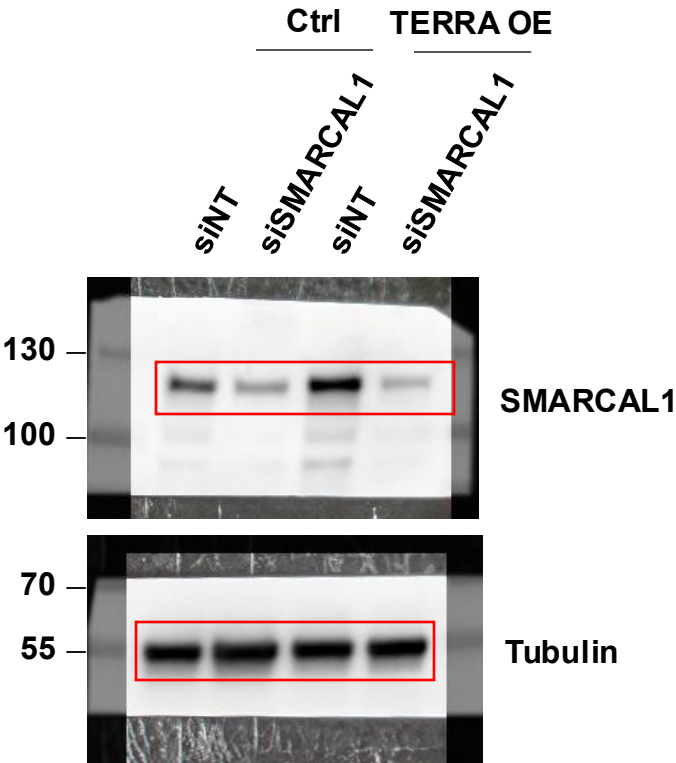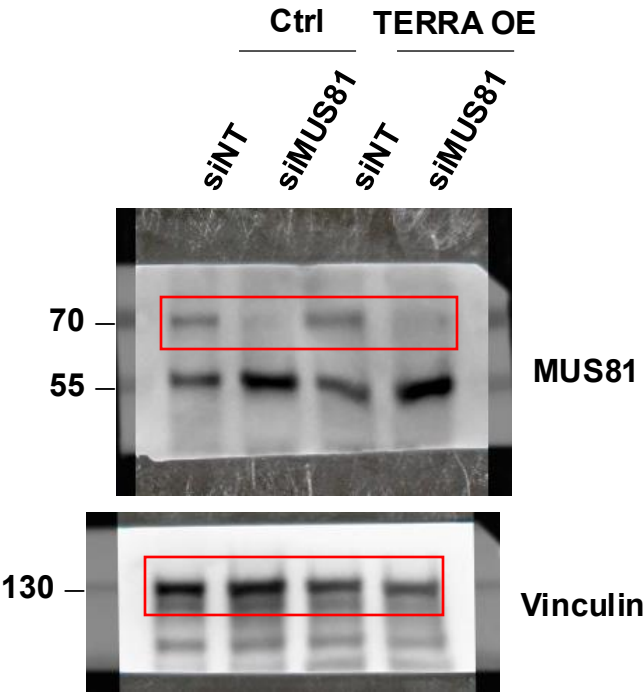

Supplement: Supplementary file 12 — Figure EV4 Source Data [file 44318_2025_502_MOESM12_ESM.zip › Figure EV4/Fig EV4E/Fig EV4E.pdf]

Fig EV4D

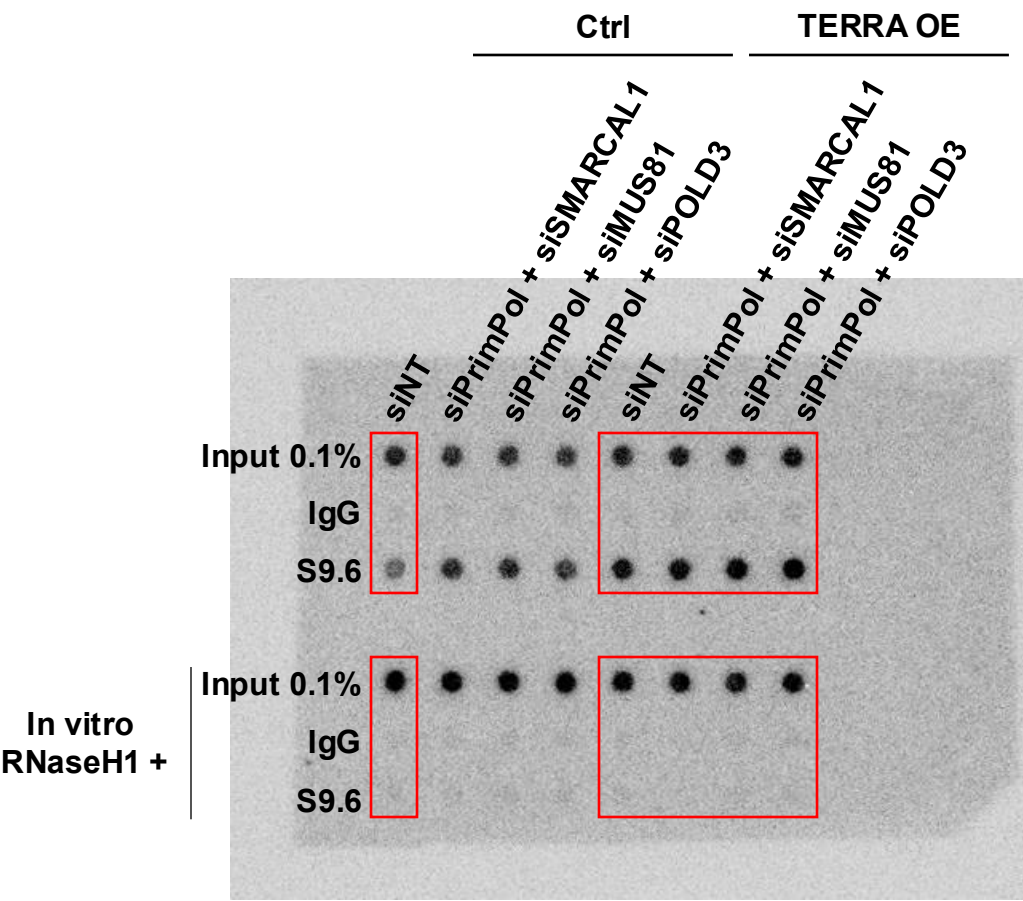

Supplement: Supplementary file 12 — Figure EV4 Source Data [file 44318_2025_502_MOESM12_ESM.zip › Figure EV4/Fig EV4D/Fig EV4D.pdf]

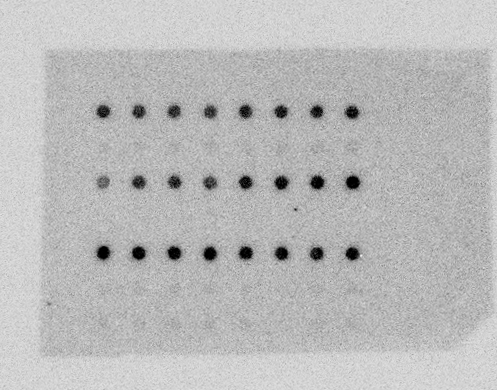

Supplement: Supplementary file 12 — Figure EV4 Source Data [file 44318_2025_502_MOESM12_ESM.zip › Figure EV4/Fig EV4D/Fig EV4D.jpg]

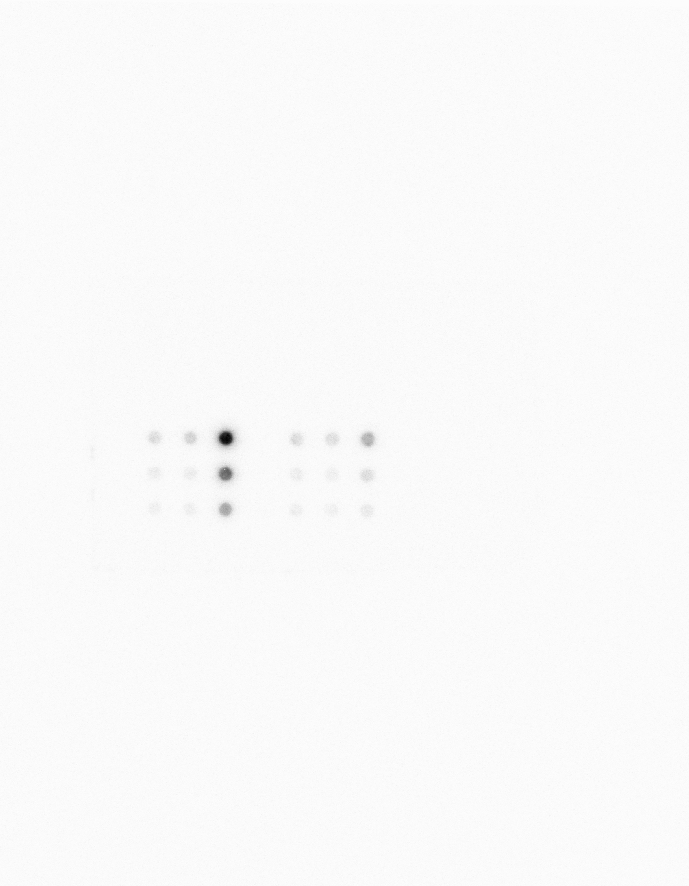

Supplement: Supplementary file 13 — Figure EV5 Source Data [file 44318_2025_502_MOESM13_ESM.zip › Figure EV5/Fig EV5D/Fig EV5D.jpg]

Fig EV5D

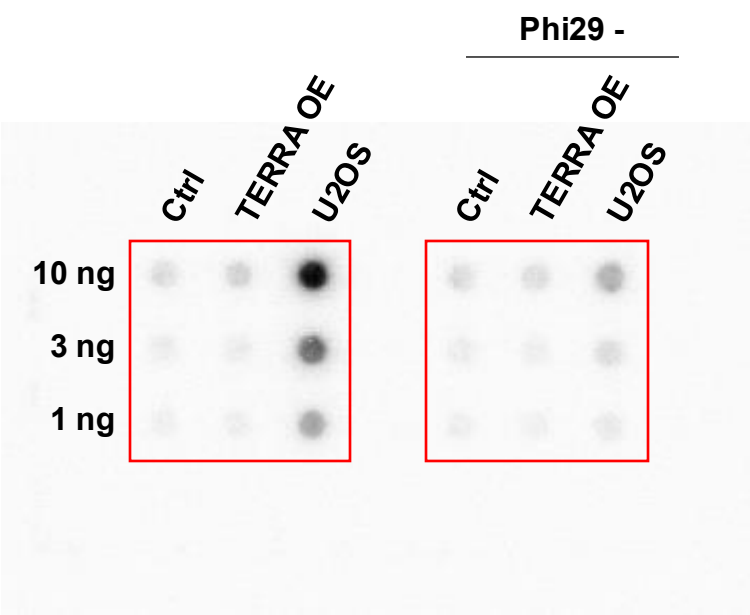

Supplement: Supplementary file 13 — Figure EV5 Source Data [file 44318_2025_502_MOESM13_ESM.zip › Figure EV5/Fig EV5D/Fig EV5D.pdf]

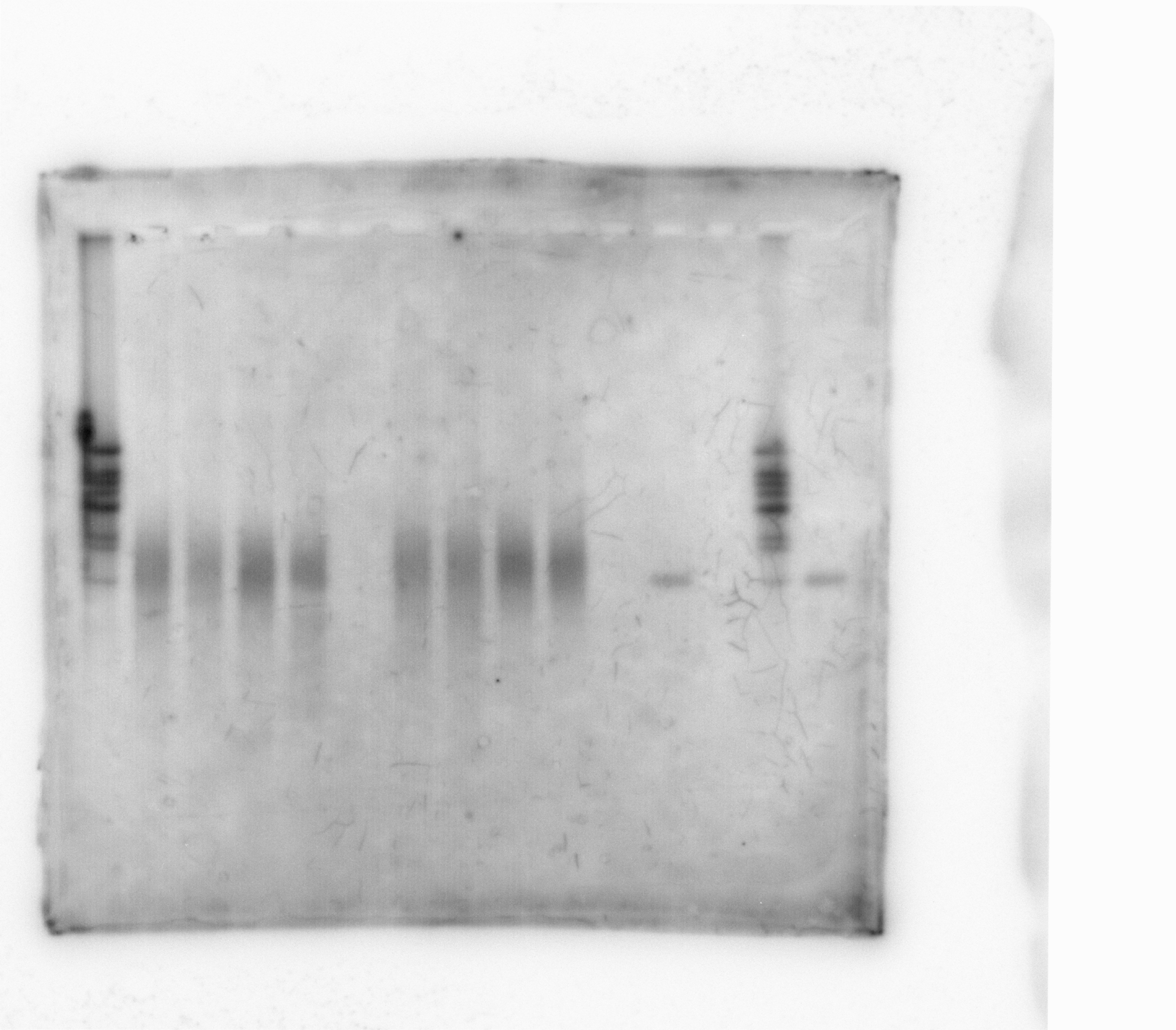

Supplement: Supplementary file 13 — Figure EV5 Source Data [file 44318_2025_502_MOESM13_ESM.zip › Figure EV5/Fig EV5C/Fig EV5C.tif]

**Fig EV5C**

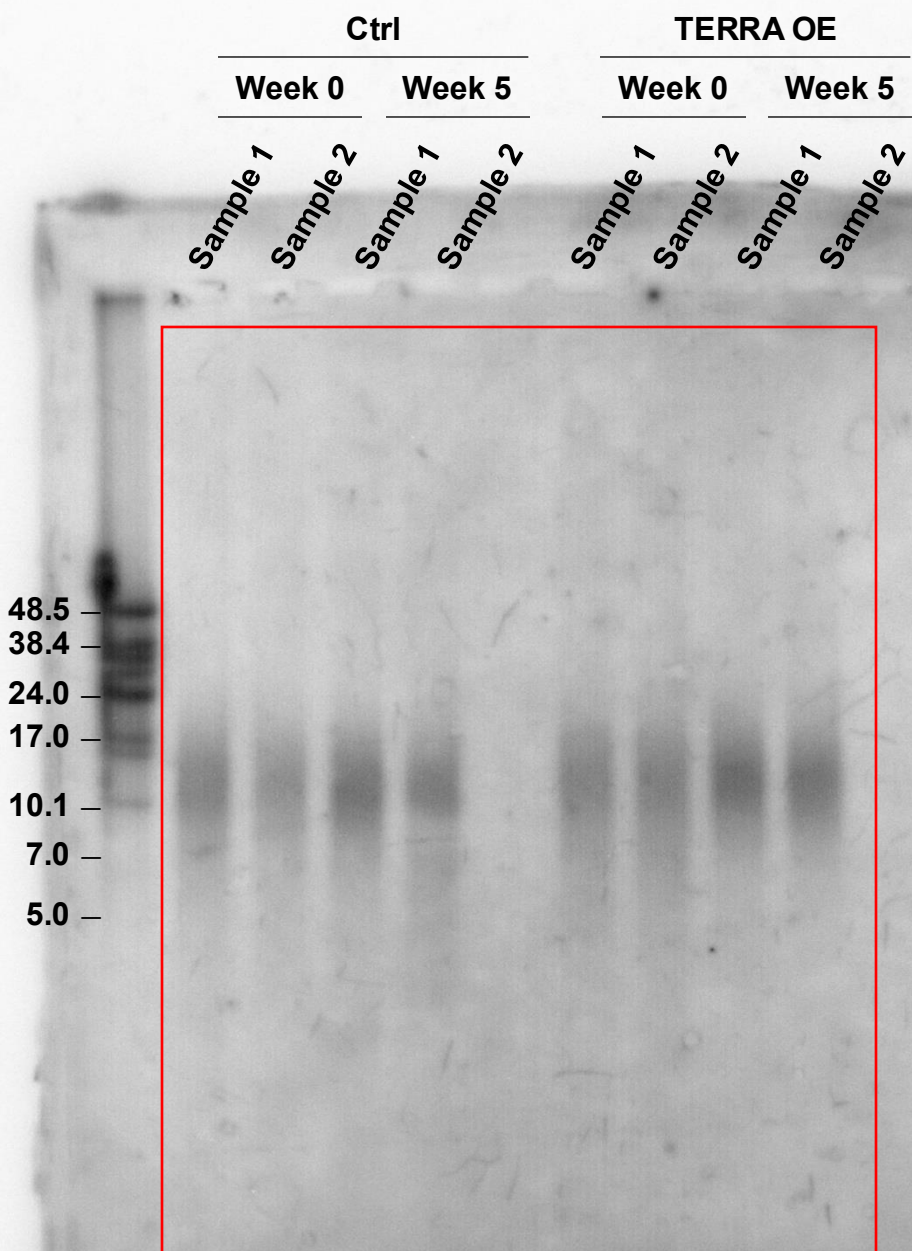

Supplement: Supplementary file 13 — Figure EV5 Source Data [file 44318_2025_502_MOESM13_ESM.zip › Figure EV5/Fig EV5C/Fig EV5C.pdf]
