# Supplementary material for: TERRA R-loops trigger a switch in telomere maintenance towards break-induced replication and PRIMPOL-dependent repair
Source: EMBO J. 2025 Jul 7;44(16):4525–51. doi: 10.1038/s44318-025-00502-4 (PMC12361433; doi:10.1038/s44318-025-00502-4)
Supplement: Supplementary file 14 — Expanded View Figures [file 44318_2025_502_MOESM14_ESM.pdf]

Expanded View Figures

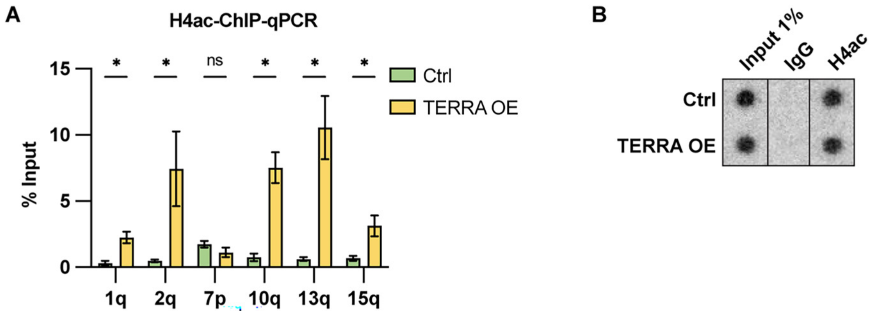

Figure EV1. Induction of endogenous TERRA by modified CRISPR-Cas9 system, related to Fig. 1.

(**A**, **B**) ChIP assay using H4 acetylation antibody. ChIP samples and inputs were treated with RNase (DNase-free) and analyzed by qPCR with indicated subtelomeric primers (**A**) or by DNA dot blot with a ^{32}P-radiolabeled telomeric probe (**B**). Multiple unpaired *t* test was applied. Data represent mean ± s.d. from three independent biological replicates. *P* values from left to right: $*P = 0.0166$, $*P = 0.0269$, ns $P = 0.3148$, $*P = 0.0290$, $*P = 0.0163$, $*P = 0.0271$. Source data are available online for this figure.

 © The Author(s)

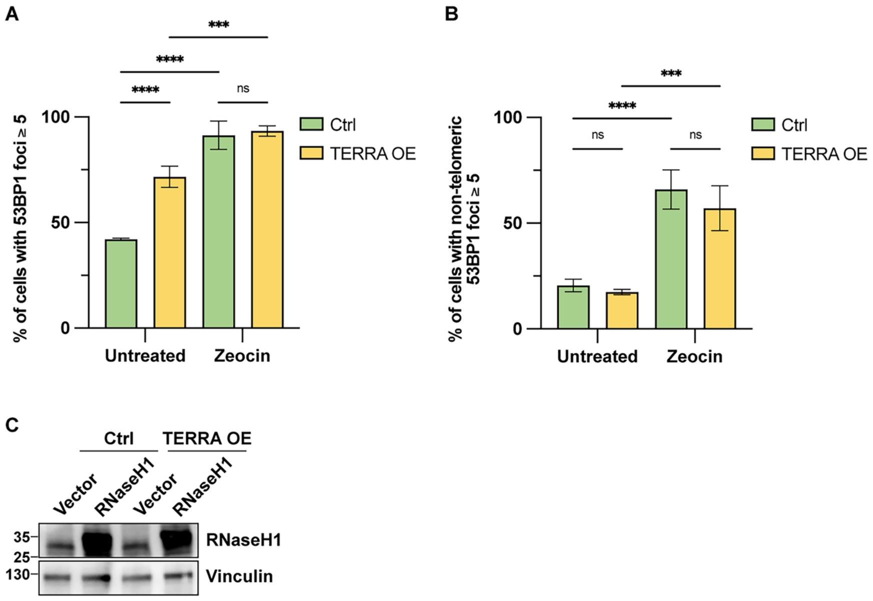

Figure EV2. DNA damage upon TERRA overexpression and zeocin treatment, related to Fig. 3.

(**A**) Quantification of the number of cells with ≥5 53BP1 foci. Data represent mean ± s.d. from three independent biological replicates. Two-way analysis of variance (ANOVA) with uncorrected Fisher's least significant difference (LSD) test was applied. *P* values from left to right: ****$P < 0.0001$, ****$P < 0.0001$, ***$P = 0.0003$, ns $P = 0.5932$. (**B**) Quantification of the number of cells with ≥5 53BP1 foci that are not colocalizing with telomeres. Data represent mean ± s.d. from three independent biological replicates. Two-way analysis of variance (ANOVA) with uncorrected Fisher's least significant difference (LSD) test was applied. *P* values from left to right: ns $P = 0.6179$, ****$P < 0.0001$, ***$P = 0.0002$, ns $P = 0.1704$. (**C**) Western blot analysis upon ectopic expression of RNaseH1 in control and TERRA-overexpressing HeLa cells. Source data are available online for this figure.

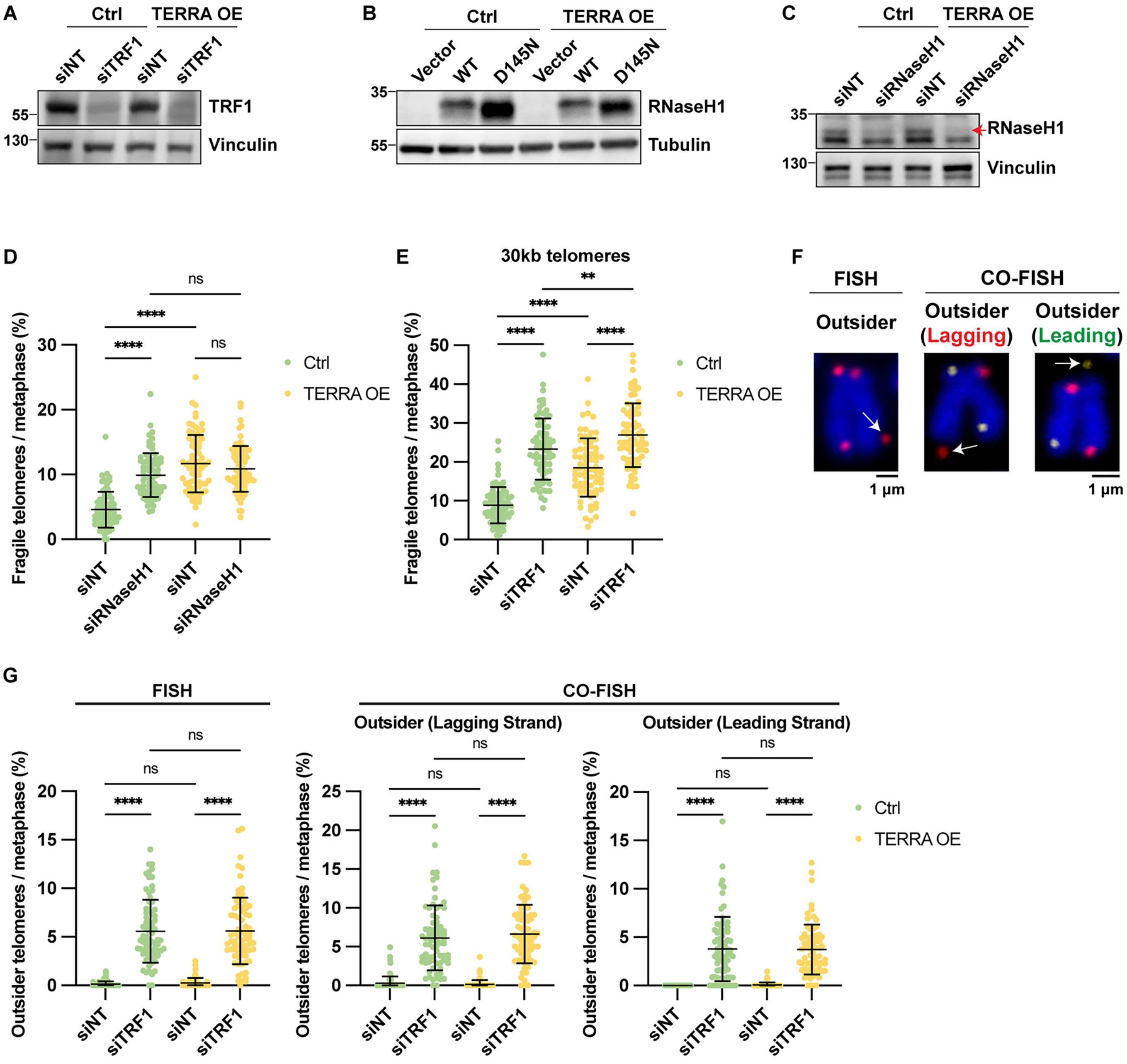

Figure EV3. Fragile and outsider telomeres, related to Fig. 4.

(**A**) Western blot analysis upon depletion of TRF1 in control and TERRA-overexpressing HeLa cells. (**B**) Western blot analysis upon ectopic expression of RNaseH1 WT or D145N mutant in control and TERRA overexpressing HeLa cells. (**C**) Western blot analysis upon depletion of RNaseH1 in control and TERRA-overexpressing HeLa cells. (**D**) Quantification of telomere fragility upon depletion of RNaseH1 in control and TERRA overexpressing HeLa cells. At least 25 metaphases were analyzed per condition per replicate, and three independent biological replicates were performed. Horizontal lines and error bars represent mean ± s.d. Two-way analysis of variance (ANOVA) with Tukey's multiple comparisons test was applied. *P* values from left to right: ****$P < 0.0001$, ****$P < 0.0001$, ns $P = 0.3221$, ns $P = 0.5391$. (**E**) Quantification of telomere fragility upon depletion of TRF1 in control and TERRA-overexpressing HeLa cells with 30 kb telomeres. At least 25 metaphases were analyzed per condition per replicate, and three independent biological replicates were performed. Horizontal lines and error bars represent mean ± s.d. Two-way analysis of variance (ANOVA) with Tukey's multiple comparisons test was applied. *P* values from left to right: ****$P < 0.0001$, ****$P < 0.0001$, ****$P < 0.0001$, **$P = 0.0012$. (**F**) Representative images of outsider telomeres by FISH (left) or CO-FISH (middle and right). Metaphase spreads were stained with telomeric $(CCCTAA)_3$-FISH probe (red) and DAPI (blue) (left), with TYE563-TeloC LNA probe (red), FAM-TeloG LNA probe (yellow), and DAPI (blue) (middle and right). (**G**) Quantification of outsider telomeres upon depletion of TRF1 in control and TERRA-overexpressing HeLa cells with 30 kb telomeres. Metaphases from the same samples were analyzed by FISH (left) and CO-FISH (middle and right). At least 25 metaphases were analyzed per condition per replicate, and three independent biological replicates were performed. Horizontal lines and error bars represent mean ± s.d. Two-way analysis of variance (ANOVA) with Tukey's multiple comparisons test was applied. *P* values from left to right: ****$P < 0.0001$, ns $P = 0.9852$, ns $P = 0.9997$, ****$P < 0.0001$ (left). ****$P < 0.0001$, ns $P = 0.9907$, ns $P = 0.7167$, ****$P < 0.0001$ (middle). ****$P < 0.0001$, ns $P = 0.9971$, ns $P = 0.9987$, ****$P < 0.0001$ (right). Source data are available online for this figure.

 © The Author(s)

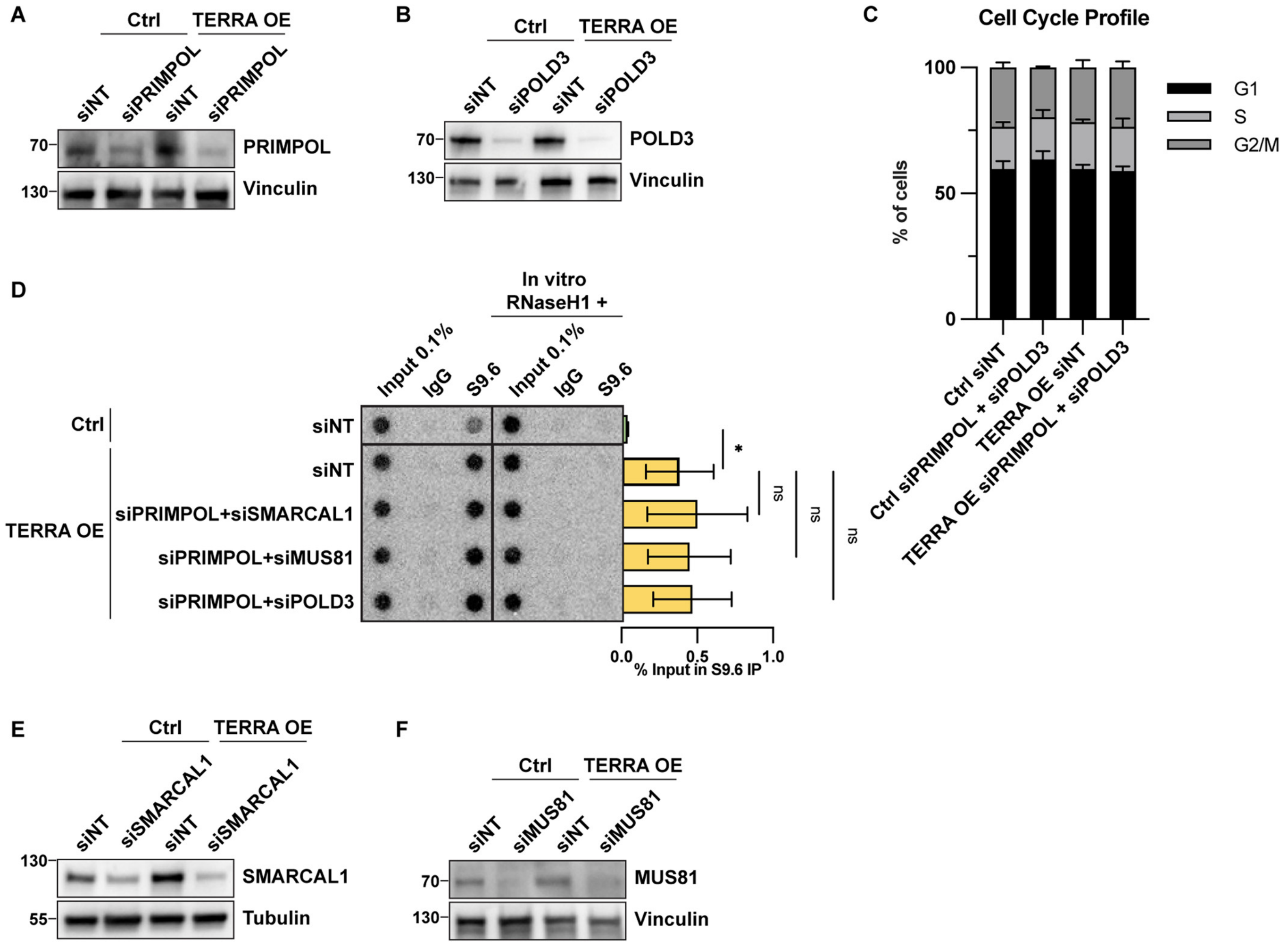

Figure EV4. Western blot analyses of depletion of proteins involved in telomere fragility, related to Fig. 5.

(**A**) Western blot analysis upon depletion of PRIMPOL in control and TERRA-overexpressing HeLa cells. (**B**) Western blot analysis upon depletion of POLD3 in control and TERRA-overexpressing HeLa cells. (**C**) Cell cycle profiles upon PRIMPOL and POLD3 depletion. DNA content was analyzed by flow cytometry analysis of fixed DAPI-stained cells at day 4 of the time course (last time point). Data represent mean ± s.d. from three independent biological replicates. (**D**) DRIP assay using S9.6 antibody. DRIP samples and inputs were treated with RNase (DNase-free) and analyzed by DNA dot blot with a ^{32}P-radiolabeled telomeric probe. As a negative control, samples were treated in vitro with RNaseH1 prior to immunoprecipitation and analyzed in parallel. Data represent mean ± s.d. from three independent biological replicates. One-way analysis of variance (ANOVA) with Šídák's multiple comparisons test was applied. *P* values from upper to lower: **P* = 0.0389, ns *P* = 0.9668, ns *P* = 0.9965, ns *P* = 0.9899. (**E**) Western blot analysis upon depletion of SMARCAL1 in control and TERRA-overexpressing HeLa cells. (**F**) Western blot analysis upon depletion of MUS81 in control and TERRA-overexpressing HeLa cells. Source data are available online for this figure.

© The Author(s)

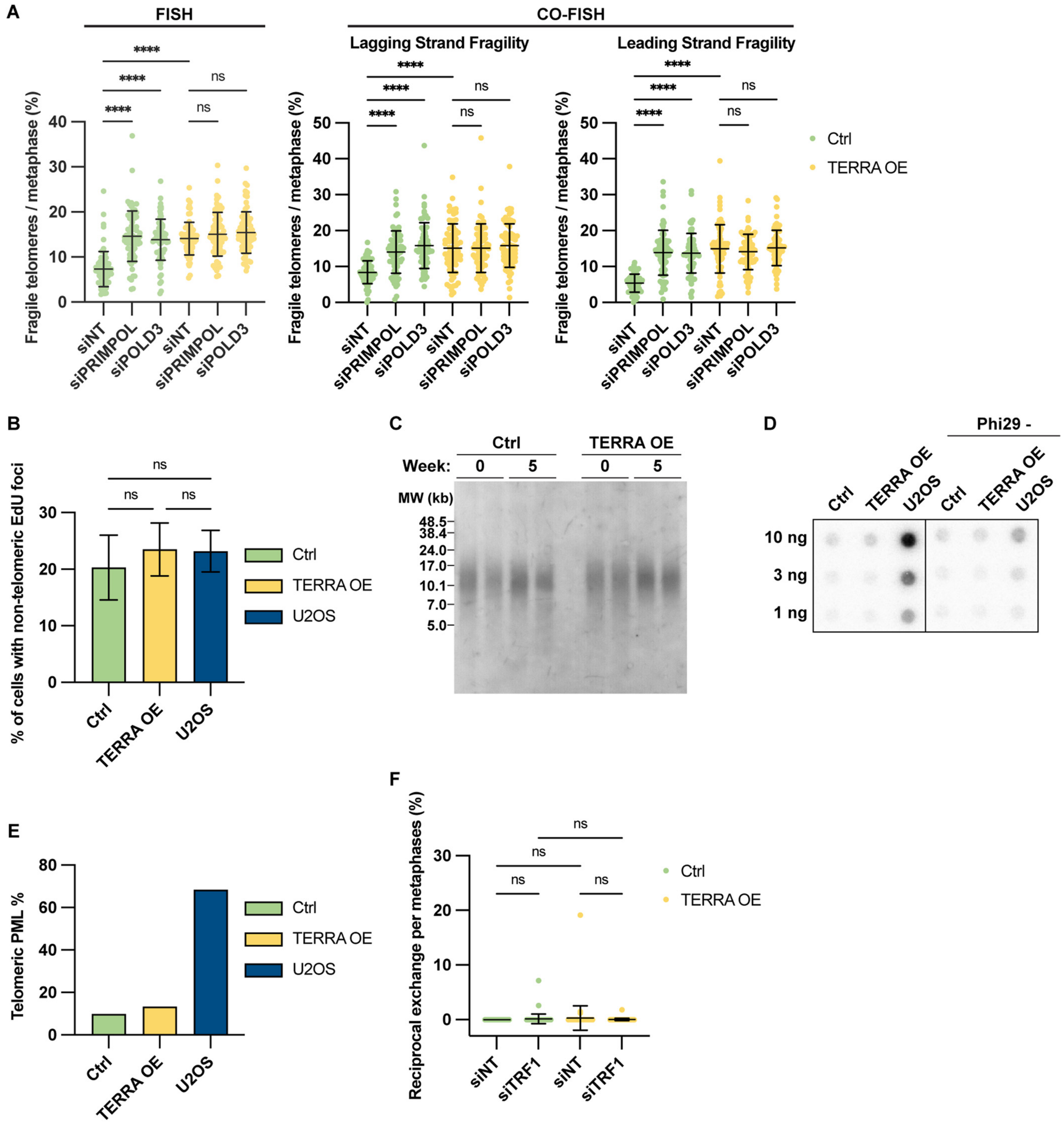
A
FISH
CO-FISH
Lagging Strand Fragility
Leading Strand Fragility
Fragile telomeres / metaphase (%)
****
ns
siNT
siPRIMPOL
siPOLD3
Ctrl
TERRA OE
B
% of cells with non-telomeric EdU foci
ns
Ctrl
TERRA OE
U2OS
C
Ctrl
TERRA OE
Week:
0
5
MW (kb)
48.5
38.4
24.0
17.0
10.1
7.0
5.0
D
Phi29 -
Ctrl
TERRA OE
U2OS
10 ng
3 ng
1 ng
E
Telomeric PML %
Ctrl
TERRA OE
U2OS
F
Reciprocal exchange per metaphases (%)
ns
siNT
siTRF1
Ctrl
TERRA OE

© The Author(s)

Figure EV5. Impacts of depletion of PRIMPOL, POLD3, SMARCAL1, and MUS81, related to Fig. 6.

(**A**) Quantification of telomere fragility upon depletion of PRIMPOL or POLD3 in control and TERRA-overexpressing HeLa cells with 30 kb telomeres. Metaphases from the same samples were analyzed by FISH (left) and CO-FISH (middle and right). At least 25 metaphases were analyzed per condition per replicate, and three independent biological replicates were performed. Horizontal lines and error bars represent mean ± s.d. Two-way analysis of variance (ANOVA) with Tukey's multiple comparisons test was applied. P values from left to right: ****$P < 0.0001$, ****$P < 0.0001$, ****$P < 0.0001$, ns $P = 0.3957$, ns $P = 0.1627$ (left). ****$P < 0.0001$, ****$P < 0.0001$, ****$P < 0.0001$, ns $P = 0.9998$, ns $P = 0.7558$ (middle). ****$P < 0.0001$, ****$P < 0.0001$, ****$P < 0.0001$, ns $P = 0.5791$, ns $P = 0.9580$ (right). (**B**) Quantification of cells exhibiting non-telomeric EdU foci. Data represent mean ± s.d. from three independent biological replicates. One-way analysis of variance (ANOVA) with Tukey's multiple comparisons test was applied. p values from left to right: ns $P = 0.7029$, ns $P = 0.7485$, ns $P = 0.9964$. (**C**) Telomere restriction fragment (TRF) analysis of control and TERRA-overexpressing HeLa cells at baseline ("Week 0," immediately before gRNA transduction) and after 5 weeks ("Week 5"). (**D**) Phi29 C-circle assay performed on 10 ng, 3 ng, and 1 ng of DNA from control and TERRA-overexpressing HeLa cells, as well as U2OS cells. Amplification products were analyzed by dot blot using a ^{32}P-labeled C-rich telomeric probe. (**E**) Quantification of PML bodies colocalizing with telomeres. Data represent one biological replicate. (**F**) Quantification of reciprocal telomeric sister chromatid exchange, as a percentage of events per metaphase spread. At least 25 metaphases were analyzed per condition per replicate, and three independent biological replicates were performed. Horizontal lines and error bars represent mean ± s.d. Two-way analysis of variance (ANOVA) with Tukey's multiple comparisons test was applied: ns indicates non-significance ($P > 0.05$). P values from left to right: ns $P = 0.9110$, ns $P = 0.4455$, ns $P = 0.9490$, ns $P = 0.5211$. Source data are available online for this figure.

© The Author(s)
